# Differential effects of SUMO1 and SUMO2 on circadian protein PER2 stability and function

**DOI:** 10.1038/s41598-021-93933-y

**Published:** 2021-07-13

**Authors:** Ling-Chih Chen, Yung-Lin Hsieh, Grace Y. T. Tan, Tai-Yun Kuo, Yu-Chi Chou, Pang-Hung Hsu, Wendy W. Hwang-Verslues

**Affiliations:** 1 Genomics Research Center, Academia Sinica, No. 128, Sec. 2, Academia Road, Taipei, 115 Taiwan, ROC; 2 Biomedical Translation Research Center (BioTReC), Academia Sinica, Taipei, 115 Taiwan, ROC; 3 Department of Bioscience and Biotechnology, National Taiwan Ocean University, Keelung City, 202 Taiwan, ROC

**Keywords:** Circadian rhythms, Post-translational modifications, Proteins

## Abstract

Posttranslational modification (PTM) of core circadian clock proteins, including Period2 (PER2), is required for proper circadian regulation. PER2 function is regulated by casein kinase 1 (CK1)-mediated phosphorylation and ubiquitination but little is known about other PER2 PTMs or their interaction with PER2 phosphorylation. We found that PER2 can be SUMOylated by both SUMO1 and SUMO2; however, SUMO1 versus SUMO2 conjugation had different effects on PER2 turnover and transcriptional suppressor function. SUMO2 conjugation facilitated PER2 interaction with β-TrCP leading to PER2 proteasomal degradation. In contrast, SUMO1 conjugation, mediated by E3 SUMO-protein ligase RanBP2, enhanced CK1-mediated PER2^S662^ phosphorylation, inhibited PER2 degradation and increased PER2 transcriptional suppressor function. PER2 K736 was critical for both SUMO1- and SUMO2-conjugation. A PER2^K736R^ mutation was sufficient to alter PER2 protein oscillation and reduce PER2-mediated transcriptional suppression. Together, our data revealed that SUMO1 versus SUMO2 conjugation acts as a determinant of PER2 stability and function and thereby affects the circadian regulatory system and the expression of clock-controlled genes.

## Introduction

The circadian clock controls many rhythmic physiological processes such as hormonal oscillation, metabolism and immune function that are essential to maintain homeostasis^1,2^. At the molecular level, the circadian clock establishes these biological rhythms through interconnected transcription-translation feedback loops (TTFLs). The core loop is composed of *BMAL*, *CLOCK*, Period (*PER1*, *PER2*) and Cryptochrome (*CRY1*, *CRY2*). BMAL-CLOCK heterodimers transcriptionally activate the expression of PER, CRY and other circadian output genes. In turn, PER and CRY suppress their own expression and suppress expression of other circadian output genes controlled by the BMAL-CLOCK complex^3,4^. PER2 suppresses BMAL/CLOCK mediated transcription by displacing BMAL/CLOCK/CRY complexes from their target promoters. This displacement is not only important for inhibition of circadian gene expression, but also essential for reactivation of the TTFL^5^. This core loop regulates expression of retinoic acid receptor–related orphan receptor (ROR) and REV-ERBα/β (NR1D1, NR1D2) which form a secondary loop that stabilizes the core loop by regulating BMAL1 and CRY1 transcription^6–8^.

The robustness and stability of circadian rhythm is also reinforced by post-translational modifications (PTMs) that determine the level, activity and subcellular localization of core clock proteins^9–14^. Although the core clock proteins are known to be regulated by several types of PTMs, it is likely that additional layers of clock-related PTM regulation remain to be discovered. Among the known PER2 PTMs, phosphorylation at two important serine residues (S662, S480) has been most intensively studied^13^. Phosphorylation at S662 affects PER2 subcellular localization and therefore influences transcription of PER2 itself and its downstream genes^15,16^. Phospho-null mutation of PER2 S662 enhances PER2 repressor function but increases PER2 turnover in the nucleus. Loss of PER2 S662 phosphorylation (typically the result of a PER2 S662G substitution) causes human Familial Advanced Sleep Phase Syndrome (FASPS) which is characterized by a dramatically shortened circadian period with a 4-h advance of sleep, temperature, and melatonin rhythms^16,17^. Phosphorylation at S480 facilitates PER2 interaction with β-TrCP thereby leading to PER2 ubiquitination and proteasomal degradation (β-TrCP phosphodegron)^18^. Conversely, acetylation of PER2 at lysine (K) residues protects PER2 from ubiquitination; whereas deacetylation of PER2 by SIRT1 leads to degradation^19^.

Recent research has shown that a phosphoswitch where CK1δ/ε can phosphorylate either of the two competing PER2 phosphorylation sites, S662 and S480, determines PER2 stability^20–22^. However, the factors that control this switch and determine which site CK1 will phosphorylate remain unknown. This illustrates how, even for a relatively well studied protein like PER2, interaction and cross regulation between PTMs that control protein function can be complex and not well understood. It is known that PER2 phosphorylation can be influenced by other PTMs. For example, O-GlcNAcylation in a cluster of serines around S662 competes with phosphorylation to regulate PER2 repressor activity^23^. Also, acetylation status of PER2 affects CK1 activity on PER2 S662^24^. Whether other PTMs are also part of this interactive regulation of PER2 that influences the speed of circadian clock^25^ remains unknown.

Despite these many lines of evidence showing the important of PER2 PTMs, little is known about PER2 SUMOylation. SUMOylation can alter protein–protein interactions, subcellular localization or protein activity and can participate in transcriptional regulation^26,27^. Usually, SUMOylation does not promote protein degradation. However, for a subset of proteins, conjugation with multiple SUMOs is critical for recognition by SUMO-targeted ubiquitin ligases (STUbLs), leading to proteasomal degradation^28^. At least three major SUMO proteins have been identified in higher eukaryotes: SUMO1 and SUMO2/3. SUMO2 and SUMO3 are 97% identical, but they share less than 50% sequence identity with SUMO1. SUMO2/3 often form SUMO chains on the substrates, whereas SUMO1 typically appears as monomers or acts as a chain terminator on SUMO2/3 polymers^29^. However, SUMO1 and SUMO2/3 may still be able to act redundantly as SUMOylation events which typically use SUMO1 can be compensated by SUMO2 or SUMO3 in SUMO1-deficient mice^30^. Conversely, conjugation by SUMO1 or SUMO2/3 can impart different fates to the SUMOylated protein. SUMO1 conjugation typically affects cellular processes including nuclear transport, cell cycle control and response to virus infection^31^, antagonizes ubiquitination^32^ and modulates protein–protein interaction^33^. In contrast, SUMO2/3 is thought to mainly participate in cellular response to stress^34^ and can facilitate targeted protein ubiquitin-mediated degradation^35^. Recently, SUMO1 and SUMO2/3 have been found to have opposite functions in mouse lens cells at different stages of development by differentially regulating the transcriptional activity of Specificity Protein 1 (SP1)^36^. Whether or not other transcriptional regulators are similarly affected by SUMO1 versus SUMO2/3 conjugation is unknown.

We found that PER2 K736 is critical for both SUMO1 and SUMO2 conjugation. However, SUMO1 versus SUMO2 conjugation had dramatically different effects on PER2 protein turnover and transcriptional suppressor function. PER2 SUMO1 conjugation may contribute to CK1-mediated S662 phosphorylation and PER2 transcriptional repressor activity. In contrast, SUMO2 promoted PER2 degradation. These data show that SUMOylation is an important layer of PER2 regulation which controls PER2 function and coordination of the circadian regulatory system.

## Results

### PER2 can be SUMOylated

**Figure 1:**
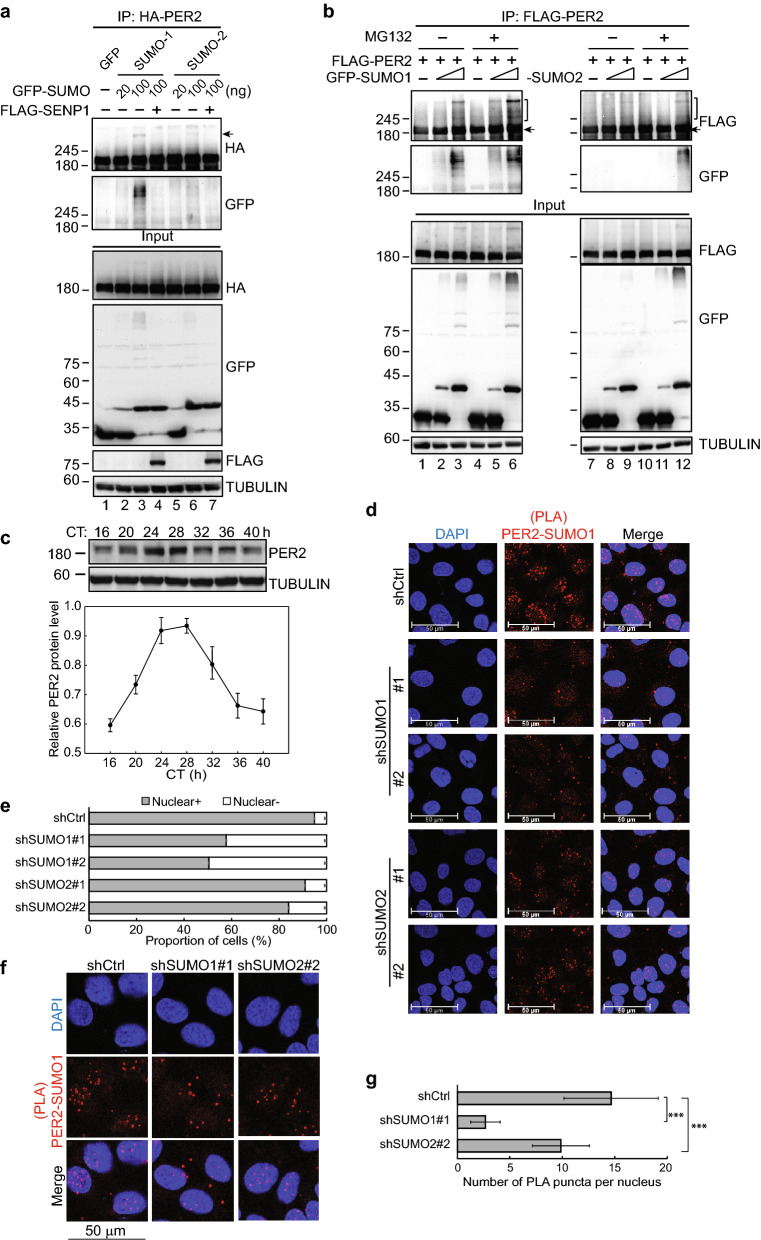
PER2 can be SUMOylated. (**a**) Co-IP assay using HEK-293 T cells co-transfected with HA-PER2, GFP-SUMOs and FLAG-SENP1. Cell lysates were immunoprecipitated (IP) with anti-HA antibody. Immunoprecipitates and input lysates were analyzed by immunoblotting (IB). Arrow indicates the PER2-SUMO conjugates. (**b**) Co-IP assay using HEK-293 T cells co-transfected with FLAG-PER2 and GFP-SUMO1 or GFP-SUMO2. Cells were also treated with the proteasome inhibitor MG132. Cell lysates were IP with anti-FLAG antibody and analyzed by IB. Brackets indicate the PER2-SUMO conjugates. (**c**) Top: time course assay using synchronized U2OS cells. After 50% serum shock, total protein was extracted at the indicated circadian time (CT). The level of endogenous PER2 was determined using immunoblotting (IB) analysis. TUBULIN was used as a loading control. Bottom: quantification of relative PER2 protein levels from six time course analyses. Data are means ± SD. (**d**) Representative confocal microscopy images of Proximity ligation assay (PLA) using U2OS cells transduced with shCtrl, shSUMO1 (clone #1 and #2) or shSUMO2 (clone #1 and #2) lentivirus and fixed at CT 28 after serum shock. PLA was performed using anti-PER2 and anti-SUMO1 antibodies against endogenous PER2 and SUMO1 proteins. The specificity of anti-SUMO1 antibody was tested using SUMO1-depleted U2OS cells (Fig. S1a, middle panel). The experiment was performed two times. Scale bar, 50 µm. (**e**) The proportion of cells with PLA puncta detected in nucleus (nucleus +) or not detected (nuclear −) was calculated from ten non-overlapping confocal images for each group (326, 320, 304, 333 and 353 cells were counted for shCtrl, shSUMO1#1, shSUMO1#2, shSUMO2#1 and shSUMO2#2, respectively). Data are proportions of cell numbers ± 95% confidence intervals. (**f**) Representative confocal microscopy images are shown for the indicated conditions. Scale bar, 50 µm. (**g**) PLA puncta per nucleus was calculated from one hundred cells for each group. Data are means ± SD. ***p < 0.001 (Student’s t-test). For (**a**–**c**), the membranes were cut prior to hybridization with antibodies. The original blots and additional experiments with similar results are shown in the “Supplementary information” file.

As an initial test of PER2 SUMOylation, we detected SUMO-conjugated PER2 in HEK-293 T cells ectopically expressing HA-taggedPER2 and GFP-SUMO1 or GFP-SUMO2 (Fig. 1a, lane 3 and 6). Co-expression of SUMO1 or SUMO2 with SENP1, a SUMO protease, eliminated the PER2 band shift (Fig. 1a, lane 4 and 7), indicating that PER2 could be SUMOylated by both SUMO1 and SUMO2. While these data indicated that PER2 could be conjugated to both SUMO1 and SUMO2, the signal of PER2-SUMO2 modification was less prominent than that of PER2-SUMO1 (Fig. 1a, lane 6 vs. lane 3). One possible explanation is that SUMO2 modification leads to PER2 ubiquitination and proteasomal degradation. Consistent with this hypothesis, treatment with the proteasome inhibitor MG132 resulted in accumulation of PER2-SUMO2 conjugates (Fig. 1b, lane 12 vs. lane 9).

Serum shock to synchronize and induce circadian gene expression^37^ showed that PER2 had a peak and trough of expression at circadian times (CT) of 28 and 40 h, respectively, in U2OS cells (Fig. 1c). We therefore further tested whether PER2 SUMOylation could occur at substantial rates in cells with endogenous PER2 and SUMO expression by fixing serum-synchronized U2OS cells at CT 28 and performing proximity ligation assays (PLA) to examine whether PER2 co-localizes with endogenous SUMO. PLA showed that ~ 95% of the cells had SUMO1-PER2 interaction. The interaction was predominantly in the nucleus with a low level of signal in the cytoplasm (Fig. 1d,e). SUMO1 knockdown significantly reduced the proportion of cells with nuclear SUMO1-PER2 interaction signals (57.8% and 50.5% for shSUMO1#1 and shSUMO1#2 transduced cells, respectively; Figs. 1d,e; S1a). The numbers of the PLA puncta in SUMO1 knockdown cells was also significantly decreased compared to control cells (2.7 puncta per nucleus for shSUMO1 versus 14.7 for shCtrl) (Fig. 1f,g). Consistent with the results in HEK293T cells, when SUMO2 was knocked down, the change in PLA signal was less extensive (Figs. 1d,e; S1a). SUMO2 knockdown decreased the number of PLA puncta (9.9 puncta per nucleus; Fig. 1f,g), but had little effect on the proportion of nuclei with positive PLA signal (90.9% and 84% for shSUMO2#1 and shSUMO2#2 transduced cells, respectively Fig. 1d,e). The observation that SUMO2 depletion also reduced PLA puncta formed between SUMO1 and PER2 indicated that PER2 may be SUMOylated at multiple sites. Some sites may be SUMOylated by SUMO2 which form SUMO2 polymers. Since SUMO1 acts as a chain terminator on SUMO2 polymers, depletion of SUMO2 can also result in reduced SUMO1‐PER2 puncta (9.9 puncta per nucleus for SUMO2‐depleted cells versus 14.7 for shCtrl cells). Together, these results indicated that PER2 may be SUMOylated and that SUMOylation, particularly by SUMO1, could be important for PER2 function.

### SUMO2 promotes PER2 protein degradation by promoting its interaction with β-TrCP

**Figure 2:**
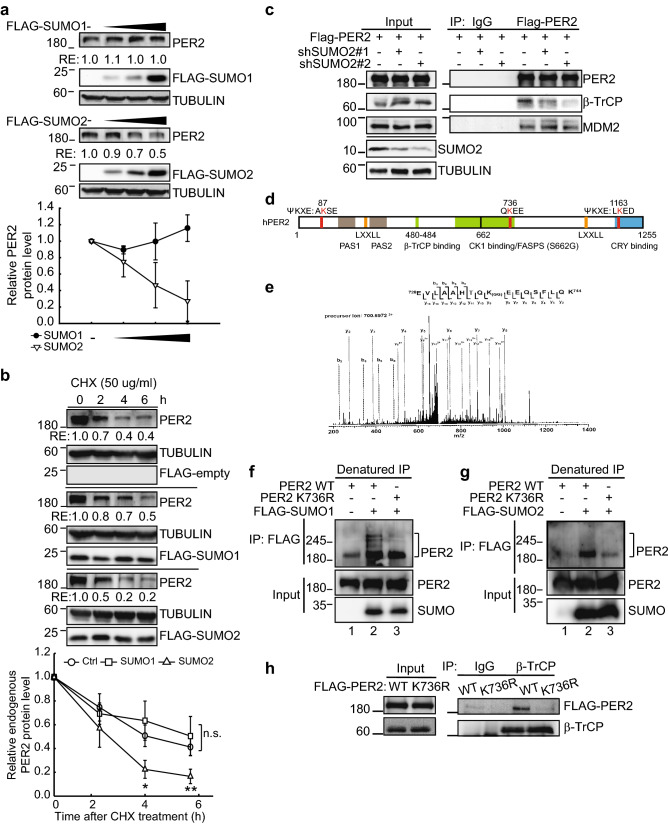
Lysine (K) 736 is important for PER2 SUMOylation and SUMO2 conjugation on K736 facilitates PER2 protein degradation via promotion of PER2-βTrCP interaction. (**a**) IB assay of endogenous PER2 and FLAG-SUMOs using U2OS cells transfected with increasing amount of FLAG-SUMOs. TUBULIN was used as a loading control. RE: relative expression. Relative PER2 protein levels from three IB analyses were quantified. Data are means ± SD. (**b**) Time course assay using cycloheximide (CHX) treated U2OS cells transfected with FLAG-SUMOs or FLAG-empty control. The levels of endogenous PER2 and FLAG-SUMOs were determined using IB analysis. TUBULIN was used as a loading control. RE: relative expression. Right panel: Quantification of relative endogenous PER2 protein levels from four IB analyses. Data are means ± SD. n.s., non-significant; *p < 0.05; **p < 0.01 (Student’s t-test). (**c**) Co-IP assay of PER2 and β-TrCP or MDM2 using U2OS cells transduced with shSUMO2 lentiviral vectors. Cell lysates were IP with indicated antibodies and analyzed by IB assay. Normal IgG was used as an IP control. An additional experiment using shSUMO2#1 and the control cells was performed and similar result was observed. (**d**) Schematic representation of human PER2 protein. Three predicted SUMOylation sites (K87, K736, K1163) are indicated (red). Coactivator LXXLL nuclear receptor recognition motifs (orange), CK1 targeted serine regions (S480, S662, green), the familial advanced sleep phase syndrome (FASPS) mutation site (S662G, black), β-TrCP binding, Per-Arnt-Sim (PAS) domains (brown) and a CRY binding domain (blue) are also indicated. (**e**) The MS/MS spectrum of the tryptic peptide m/z 700.6972 from PER2 with SUMOylated K736. All b and y product ions from the SUMOylated peptide with di-glycine modification on K736 are labelled in the spectrum. The experiment was repeated twice. (**f**) Denatured IP of PER2 and SUMO1 using HEK-293 T cells co-transfected with MYC-PER2 and FLAG-SUMOs. Cell lysates were IP with anti-FLAG antibody and analyzed by IB analysis. Brackets indicate the PER2-SUMO conjugates. The experiment was repeated twice. (**g**) Denatured IP of PER2 and SUMO2 using HEK-293 T cells co-transfected with MYC-PER2 and FLAG-SUMOs. Data presentation are as described for (**f**). (**h**) Co-IP assay of PER2 and β-TrCP using HEK-293 T cells transfected with FLAG-PER2^WT^ or -PER2^K736R^. Cell lysates were IP with indicated antibodies and analyzed by IB. Normal IgG was used as an IP control. The experiment was performed two times. For (**a**–**c**, **f**, **h**), the membranes were cut prior to hybridization with antibodies. The original blots are shown in the “Supplementary information” file.

Further experiments validated our hypothesis that SUMO2 conjugation promotes PER2 degradation. PER2 protein levels decreased in a dose-dependent manner upon SUMO2 expression but were unaffected by SUMO1 (Figs. 2a; S1b). Immunoblot (IB) assay using whole cell lysates from cycloheximide (CHX)-treated cells further showed that SUMO2 facilitated PER2 protein degradation (Figs. 2b; S1c). To demonstrate that SUMO2-conjugation promoted PER2 degradation via ubiquitination, Co-IP assay was performed using HEK-293 T cells ectopically expressing FLAG-PER2 and GFP-SUMO2. Addition of MG132 to inhibit proteasomal degradation resulted in accumulation of not only PER2-SUMO2 conjugates, but also ubiquitinated-PER2 species (Fig. S1d, lane 5, 6 vs. lane 2, 3).

PER2 can be degraded via S480 phosphodegron recognized by β-TrCP^18,38^ as well as phosphorylation-independent interaction with MDM2^39^. Therefore we performed Co-IP assay using SUMO2-depleted U2OS cells to determine which ubiquitin E3 ligase is responsible for SUMO2-mediated PER2 degradation (Fig. 2c). Consistent with previous observations, we noticed that both full length β‐TrCP (68 kD) and the short isoform (missing amino acid residue 17‐52, 65 kDa; UniProtKB—Q9Y297) was detected in U2OS cells^40^. The full length isoform (upper band) was more prominent in the IP samples, while the short isoform (lower band) was more prominent in the input. The different patterns between the input and IP indicated that SUMO2 conjugation facilitates PER2 interaction with full length β‐TrCP. Upon SUMO2 depletion, PER2 interaction with β-TrCP decreased; whereas the interaction between PER2 and MDM2 was not affected (Fig. 2c) indicating that SUMO2 conjugation is important for β-TrCP-mediated PER2 degradation.

### Lysine 736 is important for PER2 SUMOylation

The majority of known SUMO substrates are SUMOylated at a lysine in the consensus motif *ψ*KxD/E (where *ψ* is a large hydrophobic residue)^41^. However, SUMOylation can also occur at lysine residues outside this motif. Two putative PER2 SUMOylation sites at K87 and K1163 were identified using the GPS-SUMO bioinformatics database^42^ (Fig. 2d). An additional SUMOylation motif, QKEE^43^, was also identified at K736 by manual inspection of the PER2 sequence (Fig. 2d).

To determine which of these putative PER2 SUMOylation sites are functionally important, a series of K to Arginine (R) mutations at K87, K736 and/or K1163 were created. The K736R mutation clearly inhibited SUMO2-mediated PER2 degradation while K87R and K1163R had no effect (Fig. S2a). We further tested PER2 double and triple mutants and found that whenever K736 was mutated, PER2 protein became more stable regardless of whether K87 or K1163 were also mutated (Fig. S2b). These data indicated that K736 could be an important SUMOylated site of PER2. Consistent with this, liquid chromatography–tandem mass spectrometry (LC–MS/MS) using lysates prepared from HEK-293 T cells transiently expressing FLAG–PER2 and EGFP‐SUMO1 identified a SUMO peptide branch at K736 of PER2 (Fig. 2e). In addition, denatured-IP assays confirmed that the PER2 K736R mutation significantly decreased both SUMO1 and SUMO2 conjugation (Fig. 2f,g). Moreover, the PER2 K736R mutation eliminated the band shift (Fig. S2c, lane 4 vs. lane 3) that was at the same position as the eliminated shift resulting from co-expression of SUMO1 with the SUMO protease SENP1 (Fig. S2c, lane 2 vs. lane 3). This further confirmed that PER2 could be SUMOylated at K736. Also consistent with the critical importance of PER2 K736, Co-IP showed a decrease in PER2^K736R^ interaction with β-TrCP (Fig. 2h). While the mass spectrometry data demonstrated that K736 is itself SUMOylated, we do not rule out the possibility that mutation of K736 also affects SUMOylation at other unidentified sites. Together these results demonstrated that PER2 K736 is critical for PER2 SUMOylation.

### PER2 K736 is critical for maintenance of correct PER2 oscillation

**Figure 3:**
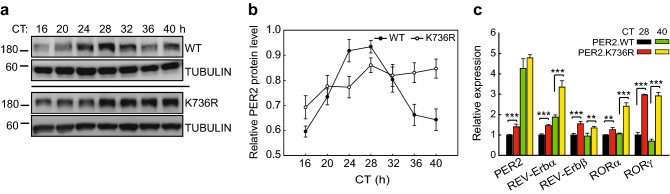
K736-dependent SUMO conjugation is required to maintain correct PER2 oscillation, PER2 nuclear localization, PER2-mediated transcriptional suppression and correct circadian period. (**a**) Time course assay using synchronized U2OS cells with or without CRISPR/CAS-edited PER2^K736R^ knock-in. After 50% serum shock, total protein was extracted at the indicated circadian time (CT). The levels of endogenous PER2 and SUMOs were determined using IB analysis. TUBULIN was used as a loading control. The membranes were cut prior to hybridization with antibodies and the original blots are shown in the “Supplementary information” file. (**b**) Quantification of relative PER2 protein levels from six time course analyses. Data are means ± SD. PER2^WT^ quantification data is acquired from the same IB analyses as in Fig. 1c. (**c**) Expression of PER2 and genes in auxiliary circadian loops analyzed by real-time quantitative (q)-PCR using PER2^K736R^ knock-in or PER2^WT^ control U2OS cells at CT 28 and CT 40 after serum shock. GAPDH was used as an internal control. All data points were performed in at least triplicates and all experiments were performed at least twice with similar results. Data are presented as mean ± SD, n = 3. **p < 0.01; ***p < 0.001 (Student’s t-test).

Stable clones of U2OS cells harboring the PER2^K736R^ mutation were generated by CRISPR/CAS editing (Fig. S3). This endogenous PER2^K736R^ mutant was more stable and did not show rhythmic oscillation after serum shock compared to PER2^WT^ (Fig. 3a,b). PER2^WT^ protein reached peak levels at CT24 and CT28 and reduced to the trough levels at CT16 and CT40. In contrast, PER2^K736R^ protein level gradually increased without reduction throughout the 24 h time course (Fig. 3a,b). These observations demonstrated that PER2 K736, and K736-dependent SUMOylation, were required for proper PER2 oscillation over a 24 h period.

### SUMOylation is critical for PER2 mediated transcriptional suppression

As PER2 is a key transcriptional suppressor in the core circadian regulation, changes in its subcellular localization and protein stability are expected to affect circadian-regulated gene expression, particularly genes in auxiliary feedback loops such as nuclear receptors REV-ERBs and RORs^6,44^. Therefore, we examined the expression of REV-ERBα, REV-ERB β, RORα and RORγ in U2OS cells expressing PER2^WT^ or endogenous PER2^K736R^ and found that PER2^K736R^ altered the expression of all these PER2-regulated genes (Fig. 3c).

In PER2^WT^ cells, PER2 and REV-ERBα were de-repressed at CT40 when PER2^WT^ expression decreased to the trough level (Fig. 3c, green vs. black). This result was consistent with the transcriptional suppressor function of PER2 in the TTFL^45^. In contrast, expression of PER2 and REV-ERBα was higher in PER2^K736R^ mutant cells compared to the PER2^WT^ cells at CT28 (Fig. 3c, red vs. black), and it reached an even higher level in PER2^K736R^ mutant cells at CT40 when the level of PER2^K736R^ remained high (Fig. 3a–c). The higher level of PER2 and REV-ERBα at CT40 could be due to an increase in BMAL1 expression since BMAL1 and PER2 express in antiphase to each other^46^. Also, PER2^K736R^ cells had higher expression of REV-ERB β, RORγ and RORα than PER2^WT^ at both CT28 and CT40 (Fig. 3c). These results confirmed that K736 modification was essential for PER2 transcriptional repression.

### PER2-SUMO1 conjugation promotes CK1 phosphorylation of PER2 S662 required for PER2 nuclear retention

PER2 must enter the nucleus to act as a transcriptional repressor and phosphorylation affects PER2 localization^15,16^. Moreover, rhythmic changes in PER2 phosphorylation are crucial for modulating circadian rhythm^18,47–50^. Two functional CK1 phosphorylation sites have been identified in human PER2, S480 (S478 in mPer2) and S662 (S659 in mPer2)^15,18^. Comparing our data with previous results, it was intriguing to note that PER2 K736-SUMO1 conjugation and S662 phosphorylation both led to increased PER2 nuclear retention (Fig. 1b–g) as lower amount of PER2^K736R^ in the soluble nuclear and chromatin-bound fractions compared to the PER2^WT^ was observed (Fig. S4a)^15^. Conversely, K736-SUMO2 conjugation and S480 phosphorylation both promoted PER2 ubiquitination and proteasomal degradation (Figs. 1g, 2a; S1b,c)^18,38^. Inhibition of CK1 using the CK1δ/ε inhibitor PF670462 led to reduced PER2 S662 phosphorylation but did not affect PER2 SUMOylation (Fig. S4b), indicating that PER2 SUMOylation does not depend upon PER2 phosphorylation. Conversely, it has been shown that SUMOylation could induce phosphorylation^51^. We therefore checked whether SUMOylation affects CK1-mediated PER2 phosphorylation and found that PER2^K736R^ showed reduced S662 phosphorylation signal compared to PER2^WT^ (Fig. S4c). This occurred despite the fact that PER2^WT^ and PER2^K736R^ had similar protein–protein interaction with CK1 (Fig. S4c). Taken together, these data indicate that K736-mediated SUMO1 conjugation may serve as a signal to promote both CK1 phosphorylation of S662 for PER2 retention in the nucleus.

### PER2 SUMO1 conjugation is mediated by RanBP2

**Figure 4:**
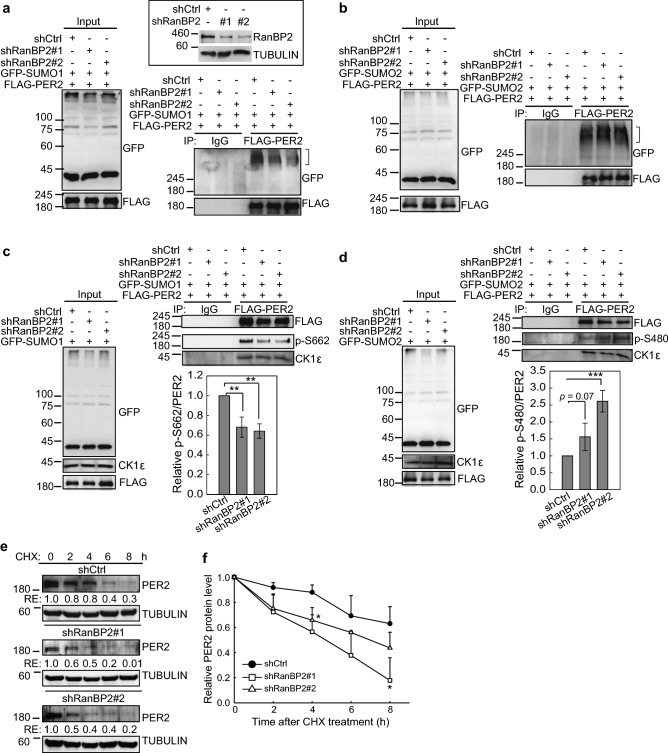
PER2 SUMO1 conjugation is mediated by RanBP2. (**a**) Co-IP assay using U2OS cells without or with RanBP2 depletion co-transfected with FLAG-PER2 and GFP-SUMO1. Cell lysates were IP with anti-FLAG antibody and analyzed by IB to detect PER2-SUMO1 conjugates indicated by brackets (lower right panel). The depletion efficiency of RanBP2 protein was determined using IB analysis (upper right panel). (**b**) Co-IP assay using U2OS cells without or with RanBP2 depletion co-transfected with FLAG-PER2 and GFP-SUMO2. Cell lysates were IP with anti-FLAG antibody and analyzed by IB to detect PER2-SUMO2 conjugates indicated by brackets. (**c**) Co-IP assay using U2OS cells without or with RanBP2 depletion co-transfected with FLAG-PER2 and GFP-SUMO1. Cell lysates were IP with anti-FLAG antibody and analyzed by IB to detect CK1 and S662-phosphorylated PER2. Quantification of relative S662-phosphorylated PER2 protein levels (p-S662/FLAG-PER2) is from three Co-IP analyses. Data are means ± SD. (**d**) Co-IP assay using U2OS cells without or with RanBP2 depletion co-transfected with FLAG-PER2 and GFP-SUMO2. Cell lysates were IP with anti-FLAG antibody and analyzed by IB to detect CK1 and S480-phosphorylated PER2. Quantification of relative S480-phosphorylated PER2 protein levels (p-S480/FLAG-PER2) from three Co-IP analyses. Data are means ± SD. (**e**) Time course assay using CHX treated U2OS cells without or with RanBP2 depletion. The levels of endogenous PER2 was determined using IB analysis. TUBULIN was used as a loading control. RE: relative expression. (**f**) Quantification of relative endogenous PER2 protein levels from three IB analyses as described in e. Data are means ± SD. *p < 0.05 (Student’s t-test). For (**a**–**e**), the membranes were cut prior to hybridization with antibodies. The original blots and additional experiments with similar results are shown in the “Supplementary information” file.

Unlike ubiquitination, E3 ligases are not required for protein SUMOylation as the SUMO E2-conjugating enzyme UBC9 is able to conjugate SUMO onto target proteins^52,53^. In the case of PER2, however, both SUMO1 and SUMO2 can be conjugated on the same K736 site. Therefore, a specific SUMO E3‐ligating enzyme may be required to mediate SUMO1 versus SUMO2 conjugation and thereby determine PER2 protein fate. As SUMO1 conjugation promoted PER2 nuclear entry, we hypothesized that the nucleoporin RanBP2, an E3 enzyme known to mediate SUMO1 conjugation to target proteins for nuclear import^26,54^, could be responsible for PER2-SUMO1 conjugation. Reduction of PER2-SUMO1 conjugation, but not PER2-SUMO2 conjugation, was observed in U2OS cells upon RanBP2 depletion (Fig. 4a,b). Consistent with this, depletion of RanBP2 in HEK-293 T cells (Fig. S4d) ectopically expressing MYC-PER2 and GFP-SUMO1 resulted in a significant decrease in PER2-SUMO1 conjugation (Fig. S4e, right panel). RanBP2 depletion significantly decreased PER2 S662-phosphorylation but did not affect interaction between PER2 and CK1 (Fig. 4c). Consistent with the “phosphoswitch” phenomenon, RanBP2 depletion in U2OS cells resulted in an increase in PER2 S480-phosphoryltaion (Fig. 4d). Also, IB assay using whole cell lysates from CHX-treated U2OS cells further showed a faster PER2 degradation upon RanBP2 depletion (Fig. 4e,f). Together, these results indicate that RanBP2-mediated PER2-SUMO1 conjugation contributes to CK1-mediated PER2 phosphorylation at S662 to prevent PER2 degradation.

## Discussion

**Figure 5:**
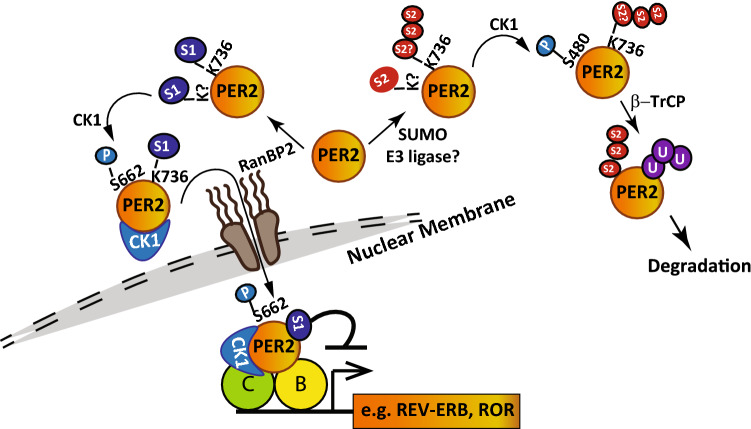
Diagram of the proposed mechanism by which SUMOylation regulates PER2 protein stability and transcriptional suppression function. PER2 K736 is important for PER2 SUMOylation by multiple SUMO isoforms. SUMO2 facilitates PER2 interaction with β-TrCP, ubiquitination and degradation. In contrast, SUMO1 modification enhances PER2 S662-phosphorylation resulting in an increase in nuclear PER2. Such modification is critical for PER2-mediated repression of circadian genes including REV-ERBα in auxiliary loops. The SUMO E3 ligase RanBP2 facilitates PER2-SUMO1 conjugation which is critical for CK1-mediated PER2 S662 phosphorylation. Identification of SUMO E3 ligases involved in SUMO2 modification of PER2 and analysis of whether SUMOylated K736 affects SUMOylation at other K sites is of interest for further experiments.

Our data indicate that SUMOylation has an important role in determining PER2 function and in maintaining PER2 protein oscillation. Consistent with previous findings that S662 phosphorylation mainly promotes PER2 protein nuclear retention and stabilization^15^, our data suggested that RanBP2-mediated PER2-SUMO1 conjugation enhances CK1-mediated PER2 phosphorylation at S662 (Fig. 4) and promotes PER2 transcriptional suppression function (Fig. 3). Conversely, PER2-SUMO2 conjugation, in line with CK1-mediated PER2 S480-phosphorylation and the β-TrCP-phosphodegron^18,21^, facilitated PER2 interaction with β-TrCP as well as PER2 ubiquitination and degradation (Fig. 2). The PER2 circadian phosphoswitch between S662 and S480 sites is determined by CK1^21^. Our data demonstrated that reducing SUMO1-conjugation by depleting RanBP2 or introduction of the PER2 K736R mutation did not affect CK1 binding to PER2 protein. However, S662 phosphorylation was reduced while S480 phosphorylation was increased (Figs. 4; S4). This indicates that SUMO1 modification on K736 may serve as a signal for CK1 to phosphorylate S662. Likewise, SUMO2 modification on K736 may prompt CK1 to phosphorylate S480. However, S480 is more distal to K736 than S662 and it is not known how SUMO2 conjugation at K736 may affect S480 phosphorylation (or vice versa). Taken together, our data indicate that PER2 SUMOylation may serve as a primary PTM that contributes to CK1-mediated PER2 phosphorylation and ubiquitination to determine PER2 function and stability (Fig. 5).

Recent evidence indicates that many SUMO targets can be modified by both SUMO1 and SUMO2/3^55^. However, the mechanisms balancing SUMO1 versus SUMO2/3 conjugation are unclear. One possibility is that differential SUMOylation is primarily due to the relative expression level of SUMO isoforms^36^. Another possibility is that SUMO1 and SUMO2/3 may have different conjugation site preferences^55,56^. In the case of PER2, K736 is important for both SUMO1 and SUMO2 conjugation (Fig. 2). Whether PER2-SUMO2 conjugation requires a specific SUMO E3 ligase or can be carried out by the SUMO-conjugating enzyme UBC9 is of interest for future research.

It should also be mentioned that PER2 SUMOylation at K736 is specific to primates. Sequence alignment from NCBI protein database showed that while primate PER2 sequence is QKEE in this region (amino acids 735/736–738/739) the corresponding region in rodents is QREE (Fig. S5). It will be interesting to further investigate whether this PER2 sequence divergence between primates and rodents is responsible for the shorter circadian period of rodents (~ 23.7 h for wild-type mice in constant darkness) versus humans (24.3–25.1 h)^57^, and whether PER2 SUMOylation is equally important for CK1-mediated PER2 phosphorylation in rodent.

In addition to its circadian role, PER2 exhibits tumor suppressive functions as a transcriptional suppressor in many cancers^58–60^. Suppression of PER2 at both gene expression and protein levels facilitates tumor growth, invasion and cancer malignancy^60–63^. Since PER2 protein level oscillates and PER2 shuttles between nucleus and cytoplasm, its protein stability and subcellular localization may also determine its tumor suppression function. Importantly, SUMO pathways are often dysregulated in cancers^64^. Such dysregulation could alter PER2-SUMO conjugation and subsequently inhibit PER2 tumor suppression function by either facilitating its degradation or inhibiting its nuclear entry. Thus, SUMO1 versus SUMO2 conjugation forms a critical crossroads controlling PER2 fate and influencing PER2 activities which extend beyond core circadian regulation. Discovery of this crossroads determining PER2 function provides new insights into the regulation of PER2 specifically as well as the function of SUMOylation in circadian regulation and health more broadly.

## Methods

### Plasmids and reagents

pcDNA3.1Myc-hPER2 plasmid was generously provided by Dr. Randal Tibbetts (University of Wisconsin, USA)^47^. pEGFP.C3.SUMO.GG plasmids were kindly provided by Dr. Mary Dasso (NIH, USA). pcDNA3.1.HA.Ub plasmid was kindly provided by Dr. Hsiu-Ming Shih (Academia Sinica, Taiwan). A series of mutant PER2 plasmids including S480A, S480D, K83R, K1163R, K736R, K83/1163R, K83/736R, K736/1163R, K83/736/1163R were made using site-directed mutagenesis (Agilent, Santa Clara, CA) with primer sets listed in Table S1. Depending on the experiments, these PER2 cDNAs were sub-cloned into various tagged vectors including pcDNA3.0.HA (Thermo Fisher Scientific, Waltham, MA), 3XFLAG-CMV-7.1-2 (Sigma-Aldrich, St. Louis, MO) and pcDNA3.1.Myc.HisA/pcDNA3.1.Myc.HisC (Thermo Fisher Scientific) for transient expression in mammalian cells. pGL3.PER2.Luc promoter (− 948 ~  + 424) was generated as previously described^63^. All cloned and mutated genes were verified by sequencing. The lentiviral shCtrl (TRC1.Scramble, ASN0000000004; TRC2.Scramble, ASN0000000003), shSUMO1#1 (TRCN0000147057), shSUMO1#2 (TRCN0000147601), shSUMO2#1 (TRCN0000007653), shSUMO2#2 (TRCN0000007655), shRANBP2#1 (TRCN0000272800) and shRANBP2#2 (TRCN0000272801) were purchased from the National RNAi Core Facility (Taipei, Taiwan). Transient transfection was done by Mirus TransIT-LT1 Reagent (Mirus Bio, Madison, WI) according to the manufacturer’s instructions or by electroporation. Subcellular protein fractionation was performed using a kit for cultured cells from Thermo Fisher Scientific (78840). Proteasome inhibitors MG132, SUMO protease inhibitor N-ethylmaleimide (NEM), cycloheximide and d-luciferin were from Sigma-Aldrich. CK1δ/ε inhibitor PF 670462 was purchased from TOCRIS (Minneapolis, MN).

### Cell line

Immortalized human bone osteosarcoma cell U2OS was obtained from the Bioresource Collection and Research Center (BCRC, Taiwan) and embryonic kidney cell HEK-293 T was obtained from the American Type Culture Collection (ATCC). Cells were maintained in DMEM supplemented with 10% fetal bovine serum and antibiotics in a humidified 37 °C incubator supplemented with 5% CO_2_. Serum shock was performed with 50% horse serum (Thermo Fisher Scientific) for 2 h when cells reached ~ 90% confluence. The medium was then replaced with medium without FBS. At the time indicated, the cells were washed twice with ice-cold PBS and whole-cell lysates prepared using RIPA lysis buffer (50 mM Tris–HCl (pH7.4), 150 mM NaCl, 1% NP40, 0.25% sodium deoxycholate, 0.05% SDS, 1 mM PMSF, 1 × protease inhibitor, with or without 10 mM NEM).

### Generation of PER2^K736R^ knock-in mutant

An all-in-one CRISPR vector, pAll-Cas9.Ppuro (RNAi core facility, Academia Sinica, Taiwan), was digested with BsmBI and ligated with annealed oligonucleotides (GGCTGCACACACACAGAAGG) for sgRNA expression to target the PER2 coding region at the K736 residue. The 5′ mismatched G of PER2 sgRNA was created for optimal U6 transcriptional initiation. A single-stranded oligodeoxynucleotide (ssODN) (5′-AAGGAGGTACTCGCTGCACACACACAGCGCGAGGAGCAGAGCTTCCTGCAGAAGTTCAAA-3′) was used to generate the K736R mutation. The underlined bases indicate the mutated codon encoding Arginine (R). Cells were transfected with all-in-one CRISPR plasmid and ssODNs donor template in the presence of NHEJ inhibitor. After selection with puromycin, monoclonal cell populations were isolated by limiting dilution method. After cell expansion, genomic DNA from selected cells was sequenced using a target-specific sequencing primer (5′-GCACTAGCCTGGGACTATTC-3′) to validate the knock-in mutation. For generation of PER2^K736R^ knock-in U2OS cells, eleven individual cell clones were analyzed and one of them was confirmed to be K736R homozygote. No heterozygote was found.

### RNA isolation, reverse transcription and real-time (q)PCR

Total RNA from cell culture was isolated using Trizol reagent or RNeasy kit (Qiagen, Hilden, Germany) and reverse-transcribed with Quant iNova Reverse Transcription kit (Qiagen). qPCR was performed using Quant iNova SYBR Green PCR kit (Qiagen) and primer sets for gene expression and analyzed on a Applied Biosystems 7300 Real-Time PCR System (Thermo Fisher Scientific). Primers used in this study were listed in Table S2. Glyceraldehyde 3-phosphate dehydrogenase (GAPDH) mRNA was used as an internal control for mRNA expression. Expression levels were calculated according to the relative ΔCt method.

### Co-immunoprecipitation (Co-IP)

Whole-cell lysates were prepared using a TNE lysis buffer (10 mM Tris–Cl (pH 7.5), 150 mM NaCl, 0.5 mM EDTA, 1% NP-40, 10 µM MG132, 1 mM PMSF, 10 mM NEM and 1 × protease inhibitors) followed by sonication and centrifugation at 12,000×*g* at 4 °C. 1 ml of the crude whole-cell extract was incubated with 1–2 μg of antibody as indicated or control IgG antibodies at 4 °C overnight. Then, 50 μl prewashed protein A/G agarose was added to the mixture and incubated at 4 °C for 4 h with gentle agitation. After extensive washing with diluted NP-40 lysis buffer (0.1–0.5% NP-40), PER2 interacting proteins were eluted with SDS buffer and analyzed by immunoblot. Antibodies and agarose used for PER2 IP were listed in Table S3.

### Denatured-IP

Denatured whole-cell lysates were prepared using a lysate buffer containing 1% SDS, 20 mM Tris–HCl (pH 8.0), 10% glycerol, 1 mM DTT, 15 mM NEM and 1 × protease inhibitors, followed by boiling at 95 °C for 8 min and sonication. After centrifugation at maximum speed, Flag-SUMO conjugated proteins were immunoprecipitated over night at 4 °C. The samples were washed using high salt RIPA buffer (20 mM Tris–HCl (pH 8.0), 0.5 mM EDTA, 250 mM NaCl, 0.5% NP40, 10% glycerol, 10 mM NEM, 1xprotease inhibitors), low salt RIPA buffer (20 mM Tris–HCl (pH 8.0), 0.5 mM EDTA, 150 mM NaCl, 0.5% NP40, 10% glycerol, 10 mM NEM, 1xprotease inhibitors), and PBS before being boiled in 2xsample buffer (with 100 mM DTT) and processed for SDS-PAGE followed by immunoblot.

### In situ proximity ligation assay (PLA)

U2OS cells were seeded at a density of 1.2 × 10^5^ cells on 12-mm glass cover slips (Hecht Assistent) 24 h before serum shock. Cells were fixed at CT 28 in 4% paraformaldehyde for 10 min and then permeabilized in 4% paraformaldehyde containing 0.15% Triton for 15 min. Immunofluorescence staining was carried out using rabbit-anti-PER2 (1:250 dilution, Santa cruz, sc-25363) and mouse-anti-SUMO1 (1:350 dilution, Sigma, SAB1402954) antibodies. The corresponding Duolink^®^ PLA Probes anti-rabbit PLA PLUS and anti-mouse PLA MINUS (1∶5 dilution) were used. Probe incubation, ligation and amplification reaction were performed using Duolink^®^ PLA Reagents (Sigma) following the manufacturer’s instruction. Cells were examined with a Leica confocal SP8 microscope (objective × 63). For each cover slip, 10–11 non-overlapping images were taken. Cells with positive signals in nucleus or nucleus and cytoplasm versus signals in cytoplasm only were counted for each condition. Numbers of PLA puncta per nucleus of one hundred cells for each condition were also counted. At least two independent experiments were performed.

### Immunoblotting (IB)

Whole-cell lysates were prepared using RIPA lysis buffer (50 mM Tris–HCl (pH7.4), 150 mM NaCl, 1% NP40, 0.25% sodium deoxycholate, 0.05% SDS, 1 mM PMSF, 1 × protease inhibitor, 10 mM NEM). IB analysis was performed after 6–7.5% SDS-PAGE or 4–20% Mini-PROTEAN TGX Precast Protein Gels (BioRad, Hercules, CA), with overnight incubation with a 1:1000 dilution of primary antibody and followed by a 1:5000 dilution of horseradish peroxidase-conjugated anti-rabbit or anti-mouse antibody (Jackson ImmunoResearch, West Grove, PA). Signals were detected using Millipore Immobilon Western Chemiluminescent HRP Substrate (Merk Darmstadt, Germany). Primary antibodies used in this study are listed in Table S3. The rabbit polyclonal antibody against phospho-Ser478 of mouse PER2 which can also detect phospho-Ser480 of human PER2 is a generous gift from Dr. David Virshup^22^. Protein concentration was determined by the Bradford assay (Bio-Rad) before loading and verified by α-tubulin level. The optical density was determined using the National Institutes of Health ImageJ program.

### Nano LC–MS/MS analysis

In order to identify the sumoylation site(s) of PER2, flag-PER2 which was expressed with and without over-expressed SUMO1(RGG) were purified by SDS-PAGE and followed by in-gel digestion for the mass spectrometric analysis. Briefly, gel slices were cut into 1 mm squares and washed in 50 mM triethylammonium bicarbonate buffer (TEABC) and then TEABC/ACN (25 mM TEABC, 50% acetonitrile). Repeated the washing steps three times. The gel slices were reduced in 20 mM dithiothreitol (DTT) at 56 °C for 1 h, and alkylated in 55 mM iodoacetamide at room temperature for 45 min in dark before trypsin digestion at 37 °C overnight. The enzymatic digestions were quenched through the addition of formic acid (10%) and the tryptic peptides were collected and vacuum-dried prior to mass analysis.

MS data were acquired on an Orbitrap Fusion mass spectrometer equipped with Dionex Ultimate 3000 RSLC system (Thermo Fisher Scientific) and nanoelectrospry ion source (New Objective, Inc., Woburn, MA). Samples in 0.1% formic acid were injected onto a self-packed precolumn (150 µm I.D. × 30 mm, 5 µm, 200 Å) at the flow rate of 10 μl/min. Chromatographic separation was performed on a self-packed reversed phase C18 nano-column (75 μm I.D. × 200 mm, 3 μm, 100 Å) using 0.1% formic acid in water as mobile phase A and 0.1% formic acid in 80% acetonitrile as mobile phase B operated at the flow rate of 300 nl/min. The MS/MS were run in top speed mode with 3 s cycles; while the dynamic exclusion duration was set to 60 s with a 25 ppm tolerance around the selected precursor and its isotopes. Monoisotopic precursor ion selection was enabled and 1+ charge state ions were rejected for MS/MS. The MS/MS analyses were carried out with the collision induced dissociation (CID) mode. Full MS survey scans from m/z 300 to 1600 were acquired at a resolution of 120,000 using EASY-IC as lock mass for internal calibration. The scan range of MS/MS spectra based on CID fragmentation method were from m/z 150 to 1600. The precursor ion isolation was performed with mass selecting quadrupole, and the isolation window was set to m/z 2.0. The automatic gain control (AGC) target values for full MS survey and MS/MS scans were set to 2e6 and 5e4, respectively. The maximum injection time for all spectra acquisition was 200 ms. For CID, the normalization collision energy (NCE) was set to 30%.

For data analysis, all MS/MS spectra were converted as mgf format from experiment RAW file by msConvert then analyzed by Mascot for MS/MS ion search. The search parameters included the error tolerance of precursor ions and the MS/MS fragment ions in spectra were 10 ppm and 0.6 Da and the enzyme was assigned to be trypsin with the miss cleavage number two. The variable post-translational modifications in search parameter were assigned to include the oxidation of methionine, carbamidomethylation of cysteine, and GlyGly tag of lysine as the sumoylation site.

### Statistical analysis

All data are presented as means ± SD. Student’s *t* test was used to identify statistically significant differences. Asterisk (*) indicates statistical significance with *p* value < 0.05, (**) indicates *p* value < 0.01, and (***) indicates *p* value < 0.001.

## Supplementary Information

Supplementary Information.
