# Supplementary material for: Differential effects of SUMO1 and SUMO2 on circadian protein PER2 stability and function
Source: Sci Rep. 2021 Jul 13;11:14431. doi: 10.1038/s41598-021-93933-y (PMC8277905; doi:10.1038/s41598-021-93933-y)

Supplementary Information

Differential Effects of SUMO1 and SUMO2 on Circadian Protein PER2 Stability and Function

Ling-Chih Chen, Yung-Lin Hsieh, Grace Y.T. Tan, Tai-Yun Kuo, Yu-Chi Chou, Pang-Hung Hsu, Wendy W. Hwang-Verslues

Supplementary Figure Legends

Figure S1. SUMO2 conjugation promotes PER2 protein ubiquitination and degradation.

a. IB analysis (left panel, using Invitrogen PA5-34765 anti-SUMO1 antibody) and representative immunofluorescence (IF) images (middle panel, using Sigma SAB1402954 anti-SUMO1 antibody) of SUMO1 in U2OS cells transduced with shCtrl or shSUMO1 lentivirus. A cell was classified by positive staining of DAPI. IB analysis (right panel) of SUMO2 in U2OS cells transduced with shSUMO2 lentivirus. TUBULIN was used as a loading control.

b. IB assay of PER2 and SUMOs using U2OS cells co-transfected with MYC-PER2 and increasing amount of GFP-SUMOs. TUBULIN was used as a loading control. RE: relative expression. Relative MYC-PER2 protein levels from three IB analyses were quantified. Data are means ± SD.

c. Top: Time course assay using cycloheximide (CHX) treated HEK-293T cells co-transfected with MYC-PER2 and FLAG-SUMOs. Cells were treated without or with MG132. The levels of PER2 and SUMOs were determined using IB analysis. TUBULIN was used as a loading control. RE: relative expression. Bottom: Quantification of relative PER2 protein levels from three IB analyses. Data are means ± SD. n.s., non-significant; *, $p < 0.05$ (Student's t-test).

d. Co-IP assay using HEK-293T cells co-transfected with FLAG-PER2 and GFP-SUMO2. Cells were treated without or with proteasome inhibitor MG132. Cell lysates were IP with

anti-FLAG antibody and analyzed by IB to detect PER2-SUMO and -UB conjugates. The experiment was performed twice with similar results.

For S1b, S1c and S1d, the membranes were cut prior to hybridization with antibodies. The original blots and additional experiments with similar results are shown in the supplementary information file.

Figure S2. Lysine residue K736 is a critical site for PER2 SUMOylation

a. Time course assay using CHX treated HEK-293T cells co-transfected with K-to-R single-site mutated MYC-tagged PER2 with or without GFP-tagged SUMO2. The levels of PER2 and SUMO2 were determined using immunoblotting. TUBULIN was used as a loading controls. RE: relative expression.

b. Time course assays using CHX treated HEK-293T cells co-transfected with K-to-R double- or triple-site mutated MYC-tagged PER2 with or without GFP-tagged SUMO2. The levels of PER2 and SUMO2 were determined using immunoblotting. TUBULIN was used as a loading controls. RE: relative expression.

c. Co-IP assay using HEK-293T cells co-transfected with MYC-PER2, GFP-SUMOs and FLAG-SENP1. Cell lysates were IP with anti-MYC antibody. Immunoprecipitates and input lysates were analyzed by IB. Arrow indicates the PER2-SUMO conjugates.

The membranes were cut prior to hybridization with antibodies. The original blots and additional experiments with similar results are shown in the supplementary information file.

Figure S3. Generation of PER2^{K736R} knock-in mutant HEK-293T cells. The WT and K736R PER2 sequences, 5'-mismatched G of PER2 sgRNA, donor template and PCR primers are shown.

Figure S4. PER2 K736-dependent SUMO1 conjugation promotes CK1 phosphorylation of PER2 S662 and CRY1/GAPVD1 interactions required for PER2 nuclear translocation and PER2-mediated transcriptional suppression.

a. Fractionation analysis using HEK-293T cells co-transfected with MYC-PER2^{WT} or -PER2 ^{K736R}. The levels of MYC-PER2 in cytoplasmic, soluble nuclear and chromatin-bound fractions were determined using IB. Tubulin was used as a loading controls for cytoplasmic proteins. Histone H3 was used as loading controls for nuclear proteins. .

b. Co-IP assay using HEK-293T cells co-transfected with FLAG-PER2^{WT} and GFP-SUMO1 treated without or with CK1 inhibitor PF670462. Cell lysates were IP with anti-FLAG antibody and analyzed by IB to detect PER2-SUMO conjugates. Normal IgG was used as an IP control. The inhibition efficiency of CK1 inhibitor was determined using IB analysis for PER2 S662-phosphorylation.

c. Co-IP assay using U2OS cells co-transfected with FLAG-PER2^{WT} or -PER2^{K736R}. Cell lysates were IP with anti-FLAG antibody and analyzed by IB to detect CK1 and S662-phosphorylated PER2.

d, e. The depletion efficiency of RanBP2 protein was determined using IB analysis (**d**). Co-IP assay using HEK-293T cells without or with RanBP2 depletion co-transfected with MYC-PER2 and GFP-SUMO1 (**e**). Cell lysates were IP with anti-MYC antibody and analyzed by IB to detect PER2-SUMO1 conjugates indicated by brackets.

The membranes were cut prior to hybridization with antibodies. The original blots and additional experiments with similar results are shown in the supplementary information file.

Figure S5.

PER2 protein sequence alignment of the region containing human K736 (QKEE motif) between primates and rodent.

Table S1. Primers for site-directed mutagenesis

K87R	5'-GATGGGTTGTGTTCAGATCTTGCCATCATCAGGCTAAA-3'<br>3'-CTACCCAACACAAGTCTAGAACGGTAGTAGTCCGATTT-5'
K736R	5'- CTCGCTGCACACACACAGAGGGAGGAGCAG -3'<br>3'-GAGCGACGTGTGTGTGTCTCCCTCCTCGTC-5'
K1163R	5'-TCTCTGTCCTCCCTCAAAACCGCTTCTAAATTTCGG-3'<br>3'-AGAGACAGGAGGGAGTTTTGGCGAAGATTTAAAGCC-5'

Table S2. Primer sets for qPCR

GADPH	Forward	TGCACCACCAACTGCTTAGC
	Reverse	GGCATGGACTGTGGTCATGAG
PER2	Forward	GGATGCCCGCCAGAGTCCAGAT
	Reverse	TGTCCACTTTCGAAGACTGGTCGC
REV-Erbα	Forward	GACATGACGACCCTGGACTC
	Reverse	GCTGCCATTGGAGTTGTCAC
REV-Erbβ	Forward	GTTACCTGTGCAACACTGGAGG
	Reverse	ACTGCTGGAGTGAAGCTCATCG
RORα	Forward	CACCAGCATCAGGCTTCTTTCC
	Reverse	GTATTGGCAGGTTTCCAGATGCG
RORγ	Forward	CAGCGCTCCAACATCTTCT
	Reverse	CCACATCTCCCACATGGACT

Table S3. Antibodies

Antibody	Catalog number	Dilution	Company
CRY1	SC-101006	1:1000	Santa Cruze
CK1ε	12448	1:1000	Cell signaling
GFP	2555S	1:1000	Cell signaling
GAPex5 (GAPVD1)	A302-116A	1:1000	Bethyl
PER2(H90)	SC-25363	1:1000	Santa Cruze
PER2 (phosphor-S662)	Ab206377	1:1000	Abcam
PER2 (phospho-S480)		1:500	(*1*)
RanBP2	Ab64276	1:1000	Abcam
SUMO1	PA5-34765	1:1000	Invitrogen
SUMO1	SAB1402954	1:200; 1:350	Sigma
SUMO2	PA5-11373	1:1000	Thermo
β-TrCP	4394S	1:1000	Cell signaling
UB	ab7780	1:1000	Abcam
HA.11 epitope Tag	901503	1:1000	BioLegend
FLAG-M2	F3165	1:1000	Sigma
Myc-tag	ab9106	1:1000	Abcam
alpha tubulin	GTX628802	1:10000	GeneTex
Nuclear Matrix Protein p84	GTX70220	1:1000	GeneTex

For Co-IP assays, 1-2 ug per IP was used.
For SUMO1 detection using SAB1402954 anti-SUMO1 antibody, 1:200 dilution was used in IF assays and 1:350 dilution was used in PLA assays.

1. M. Zhou, J. K. Kim, G. W. Eng, D. B. Forger, D. M. Virshup, A Period2 Phosphoswitch Regulates and Temperature Compensates Circadian Period. Mol Cell 60, 77-88 (2015).

Figure S1

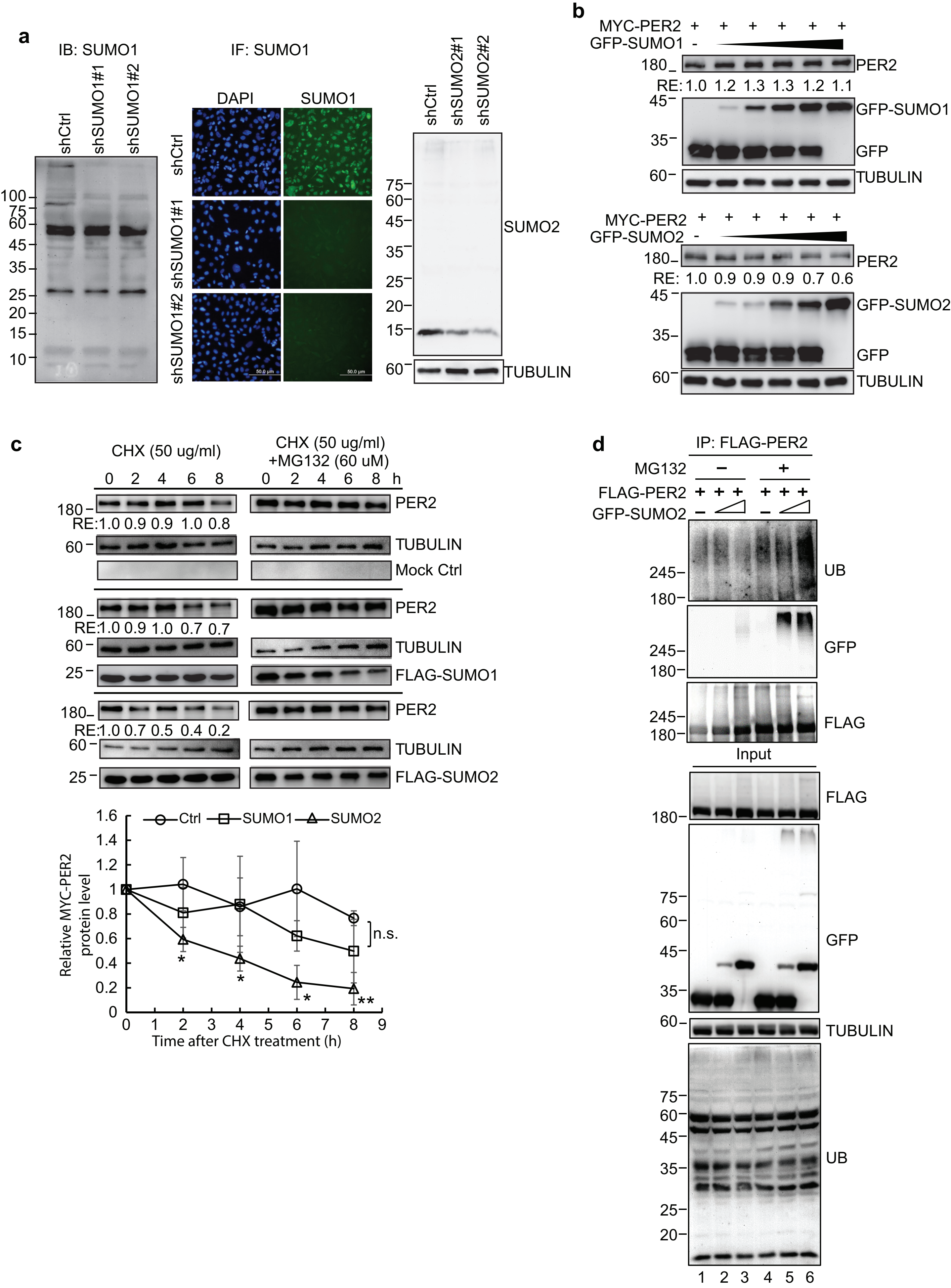

a
IB: SUMO1
shCtrl
shSUMO1#1
shSUMO1#2
IF: SUMO1
DAPI
SUMO1
shCtrl
shSUMO2#1
shSUMO2#2
SUMO2
TUBULIN
b
MYC-PER2
GFP-SUMO1
PER2
RE: 1.0 1.2 1.3 1.3 1.2 1.1
GFP-SUMO1
GFP
TUBULIN
MYC-PER2
GFP-SUMO2
PER2
RE: 1.0 0.9 0.9 0.9 0.7 0.6
GFP-SUMO2
GFP
TUBULIN
c
CHX (50 ug/ml)
CHX (50 ug/ml) +MG132 (60 uM)
0 2 4 6 8 h
PER2
RE:1.0 0.9 0.9 1.0 0.8
TUBULIN
Mock Ctrl
PER2
RE:1.0 0.9 1.0 0.7 0.7
TUBULIN
FLAG-SUMO1
PER2
RE:1.0 0.7 0.5 0.4 0.2
TUBULIN
FLAG-SUMO2
Ctrl
SUMO1
SUMO2
Relative MYC-PER2 protein level
Time after CHX treatment (h)
n.s.
d
IP: FLAG-PER2
MG132
FLAG-PER2
GFP-SUMO2
UB
GFP
FLAG
Input
FLAG
GFP
TUBULIN
UB
1 2 3 4 5 6

Figure S2

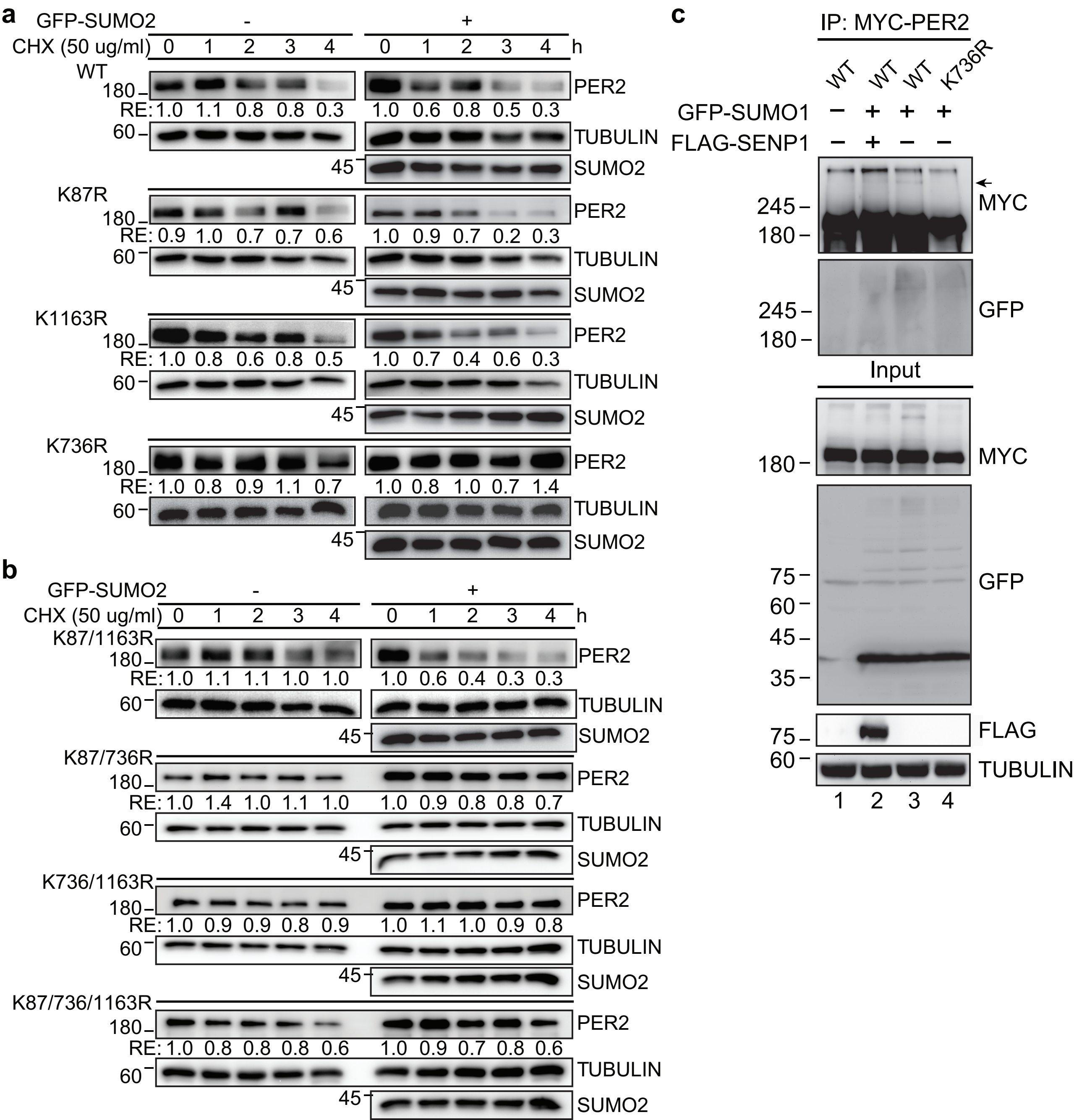

a
GFP-SUMO2 - +
CHX (50 ug/ml) 0 1 2 3 4 0 1 2 3 4 h
WT
180
PER2
RE: 1.0 1.1 0.8 0.8 0.3 1.0 0.6 0.8 0.5 0.3
60
TUBULIN
45
SUMO2
K87R
180
PER2
RE: 0.9 1.0 0.7 0.7 0.6 1.0 0.9 0.7 0.2 0.3
60
TUBULIN
45
SUMO2
K1163R
180
PER2
RE: 1.0 0.8 0.6 0.8 0.5 1.0 0.7 0.4 0.6 0.3
60
TUBULIN
45
SUMO2
K736R
180
PER2
RE: 1.0 0.8 0.9 1.1 0.7 1.0 0.8 1.0 0.7 1.4
60
TUBULIN
45
SUMO2
b
GFP-SUMO2 - +
CHX (50 ug/ml) 0 1 2 3 4 0 1 2 3 4 h
K87/1163R
180
PER2
RE: 1.0 1.1 1.1 1.0 1.0 1.0 0.6 0.4 0.3 0.3
60
TUBULIN
45
SUMO2
K87/736R
180
PER2
RE: 1.0 1.4 1.0 1.1 1.0 1.0 0.9 0.8 0.8 0.7
60
TUBULIN
45
SUMO2
K736/1163R
180
PER2
RE: 1.0 0.9 0.9 0.8 0.9 1.0 1.1 1.0 0.9 0.8
60
TUBULIN
45
SUMO2
K87/736/1163R
180
PER2
RE: 1.0 0.8 0.8 0.8 0.6 1.0 0.9 0.7 0.8 0.6
60
TUBULIN
45
SUMO2
c
IP: MYC-PER2
WT WT WT K736R
GFP-SUMO1 − + + +
FLAG-SENP1 − + − −
245
180
MYC
245
180
GFP
Input
180
MYC
75
60
45
35
GFP
75
FLAG
60
TUBULIN
1 2 3 4

Figure S3

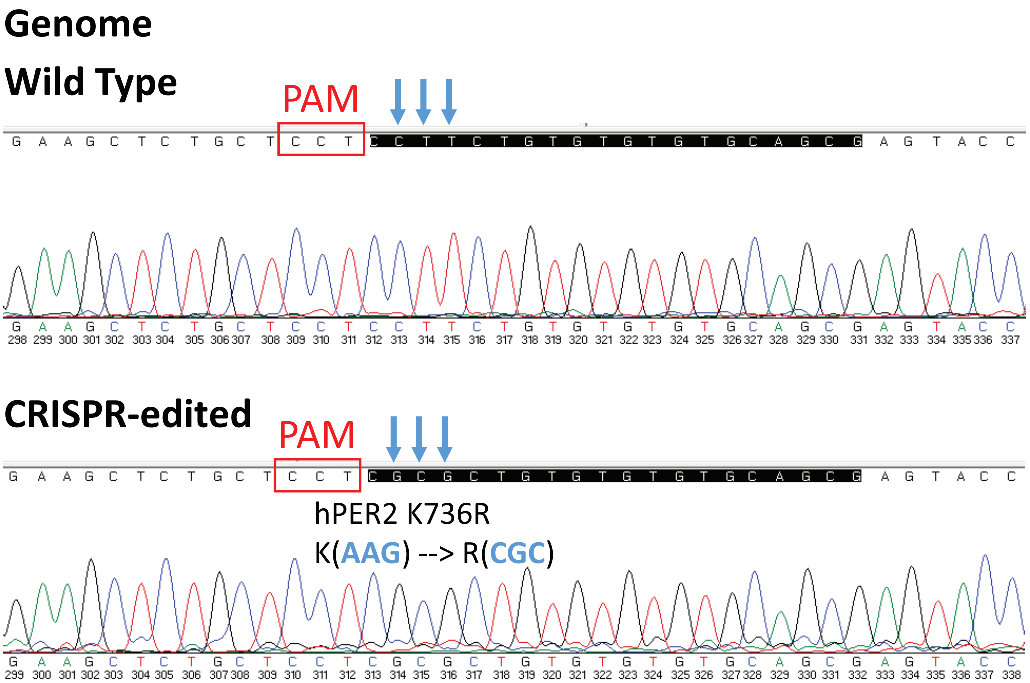

sgRNA:
GGCTGCACACACACAGAAGG **(AGG)**

Donor template (ssDNA):
AAGGAGGTACTCGCTGCACACACACAGCGCGAGGAG
CAGAGCTTCCTGCAGAAGTTCAAA

hPER2-PCR-F
TGTGGTCTCAGCCAAGAGAAGG

hPER2-PCR-R
GGCAAGAAATGTGGACATGGCA

Figure S4

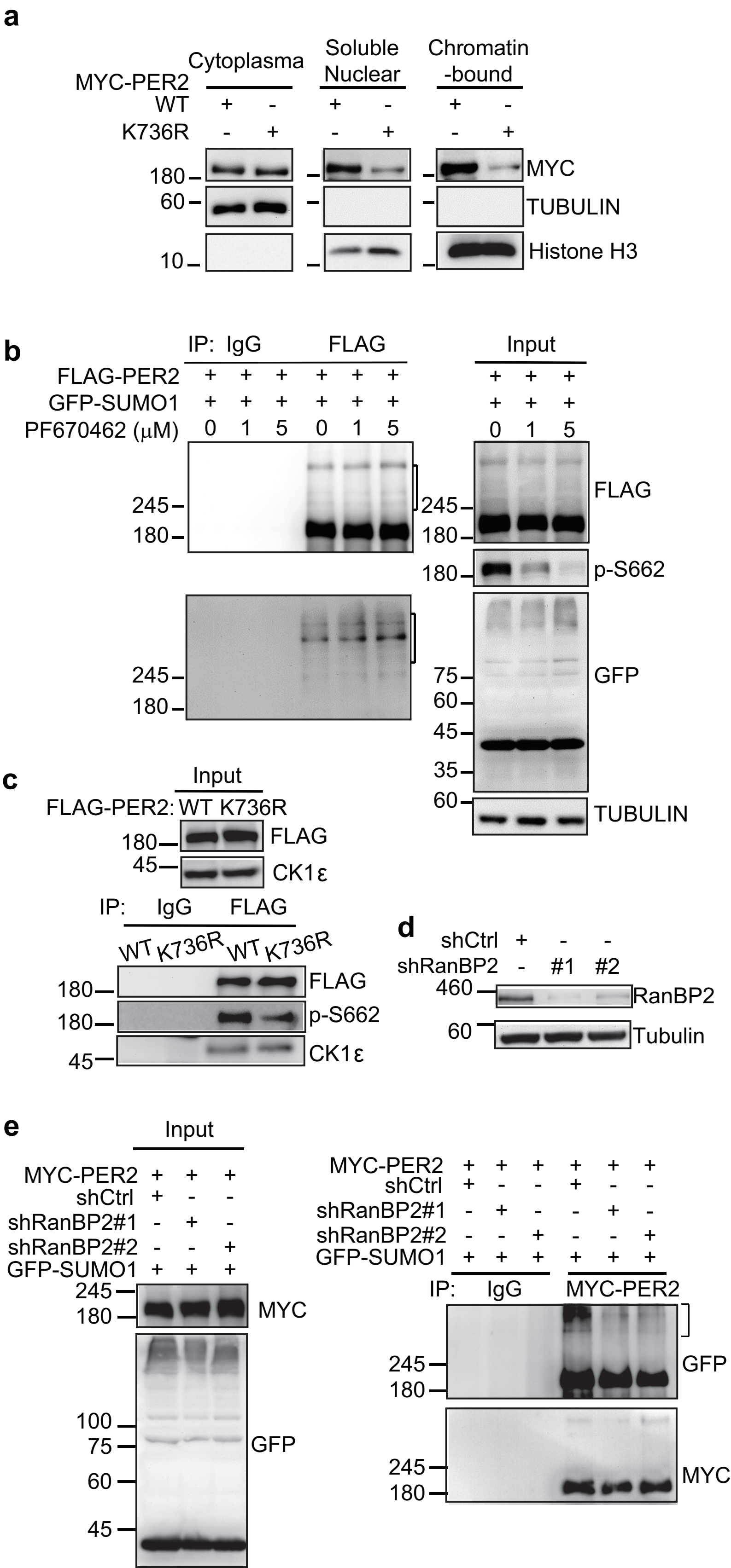

a
MYC-PER2
Cytoplasma
Soluble Nuclear
Chromatin -bound
WT + - + - + -
K736R - + - + - +
180
60
10
MYC
TUBULIN
Histone H3
b
IP: IgG FLAG
Input
FLAG-PER2 + + + + + + + + +
GFP-SUMO1 + + + + + + + + +
PF670462 (μM) 0 1 5 0 1 5 0 1 5
245
180
FLAG
p-S662
GFP
TUBULIN
75
60
45
35
c
Input
FLAG-PER2: WT K736R
FLAG
CK1ε
45
IP: IgG FLAG
WT K736R WT K736R
p-S662
d
shCtrl + - -
shRanBP2 - #1 #2
460
RanBP2
Tubulin
e
MYC-PER2 + + +
shCtrl + - -
shRanBP2#1 - + -
shRanBP2#2 - - +
GFP-SUMO1 + + +
MYC
100
IP: IgG MYC-PER2

Figure S5

	Amino Acid Sequence	NCBI Reference Sequence
Homo sapiens	721 KKLGLTKEVL AAHTQKEEQS 740	NP_073728.1
Macaca fascicularis	721 KKLGLTKEVL AAHTQKEEQS 740	XP_005574848.1
Aotus nancymaae	722 KKLGLTKEVL AAHTQKEEQS 741	XP_021520744.1
Callithrix jacchus	722 KNLGLTKEVL AAHTQKEEQS 741	XP_008998020.1
***Mus musculus** (C57BL/6)*	713 QKLGLTKEVL AAHTQREEQG 732	NP_035196.2
Microtus ochrogaster	713 QKLGLTKEVL AAHTQREEQG 732	XP_013207202.1
Cavia porcellus	713 VLASHTQKEE QSFLQKFREV 732	XP_013013057.1

Figure 1a

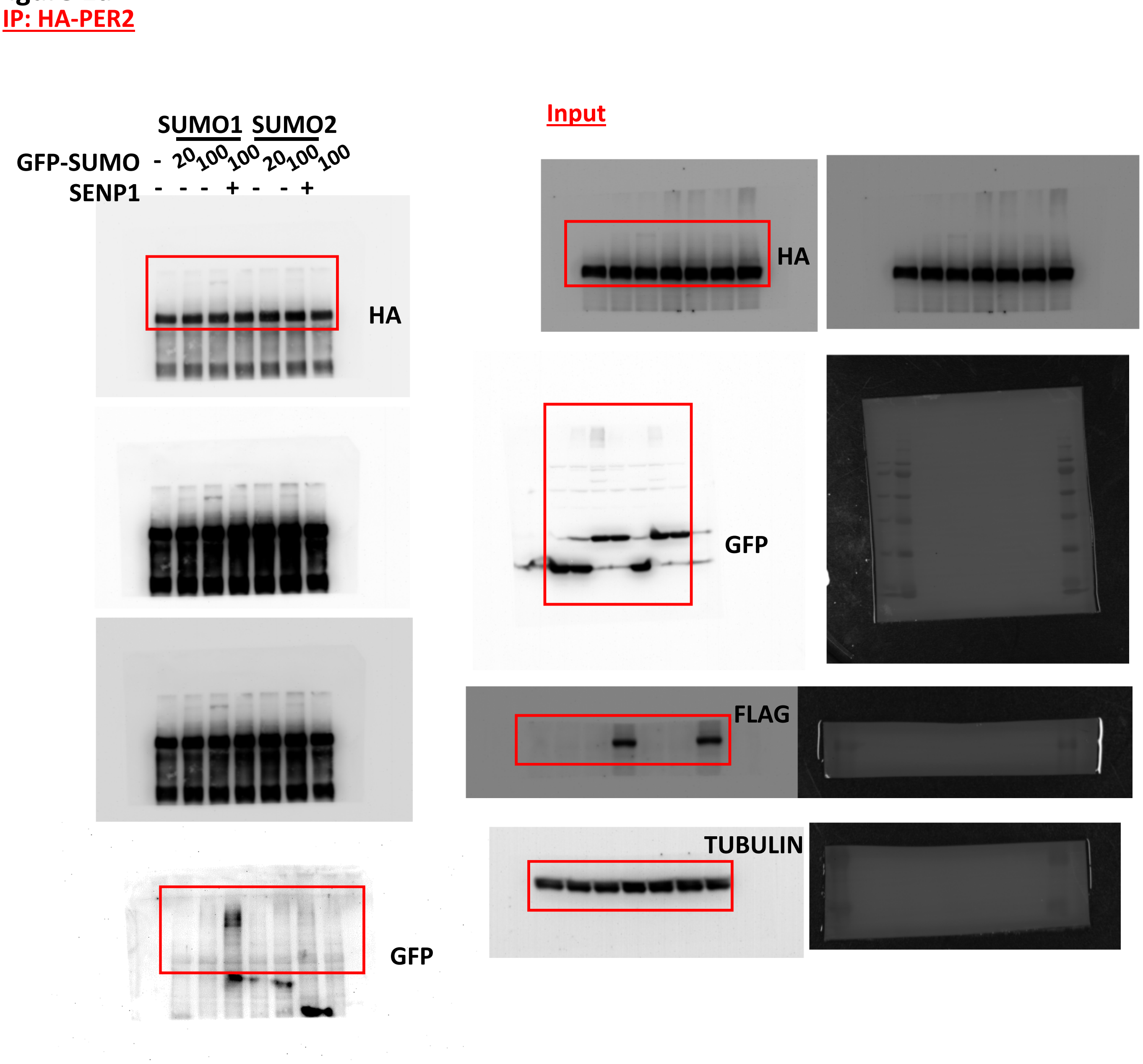
IP: HA-PER2
SUMO1 SUMO2
GFP-SUMO - 20 100 100 20 100 100
SENP1 - - - + - - +
HA
GFP
Input
HA
GFP
FLAG
TUBULIN

Figure 1a (additional experiments)

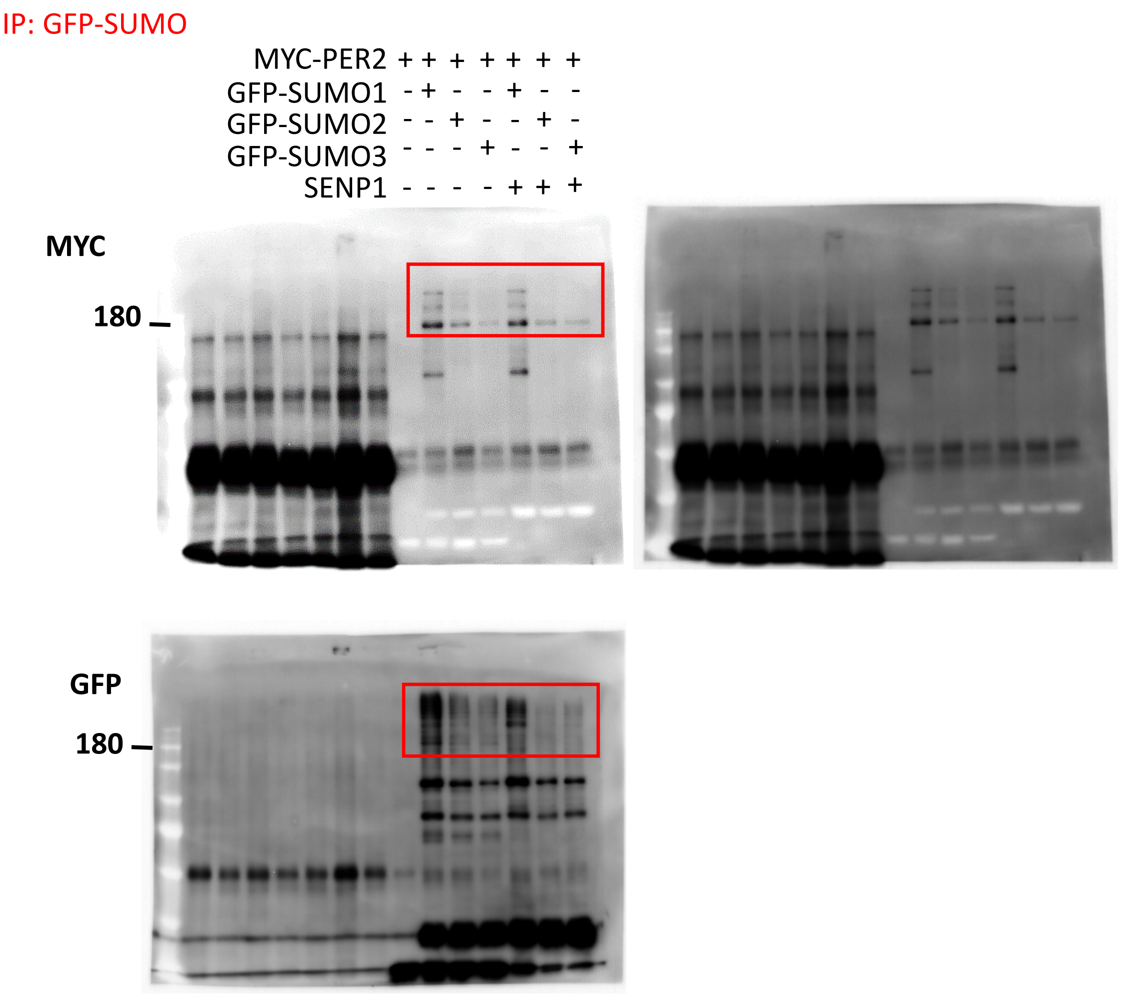

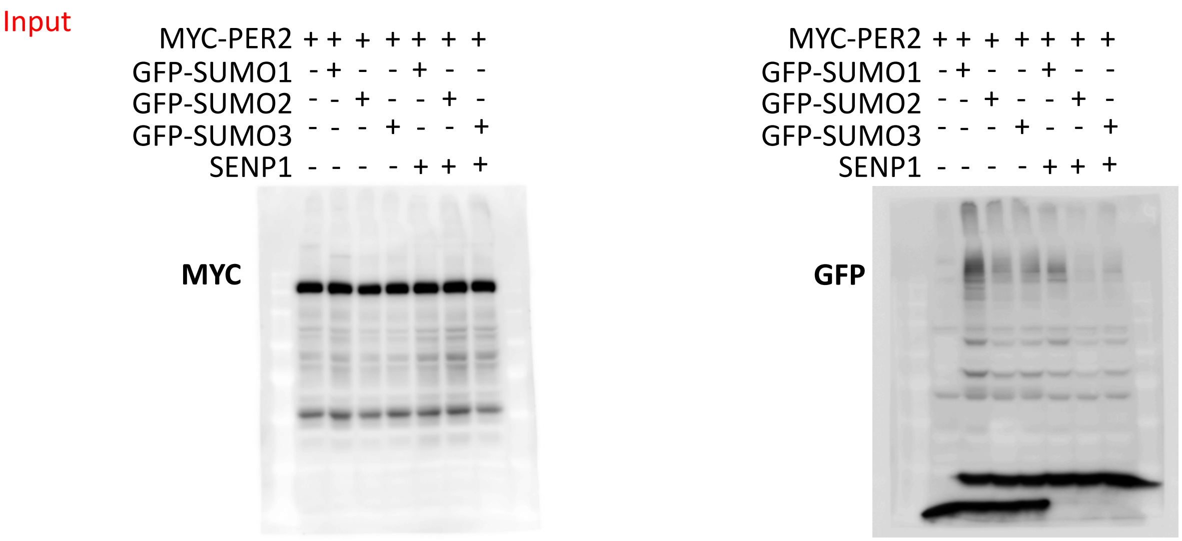

Figure 1b

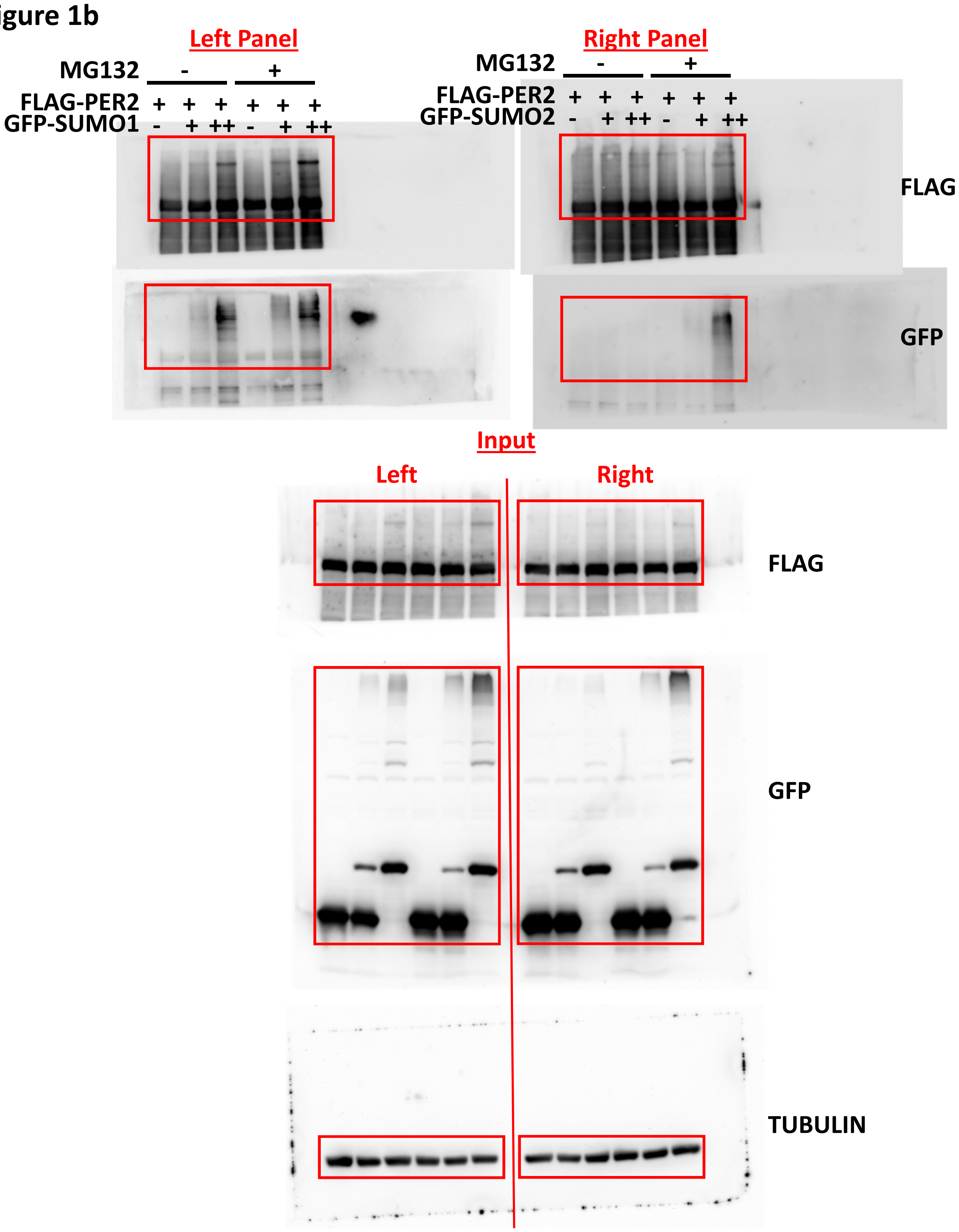
Left Panel
MG132 - +
FLAG-PER2 + + + + + +
GFP-SUMO1 - + ++ - + ++
Right Panel
MG132 - +
FLAG-PER2 + + + + + +
GFP-SUMO2 - + ++ - + ++
FLAG
GFP
Input
Left
Right
FLAG
GFP
TUBULIN

Figure 1b (additional experiments)

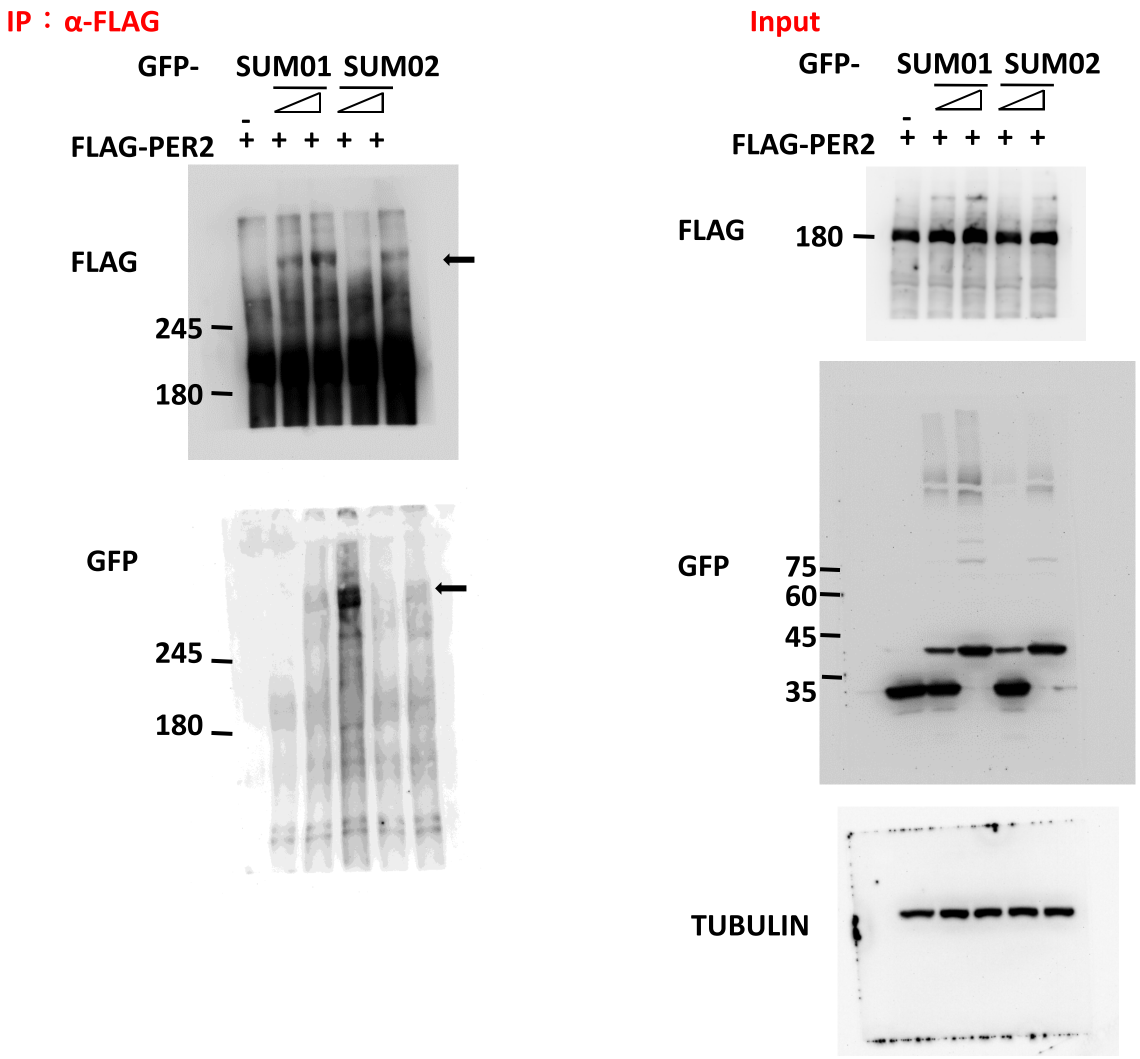

Figure 1c

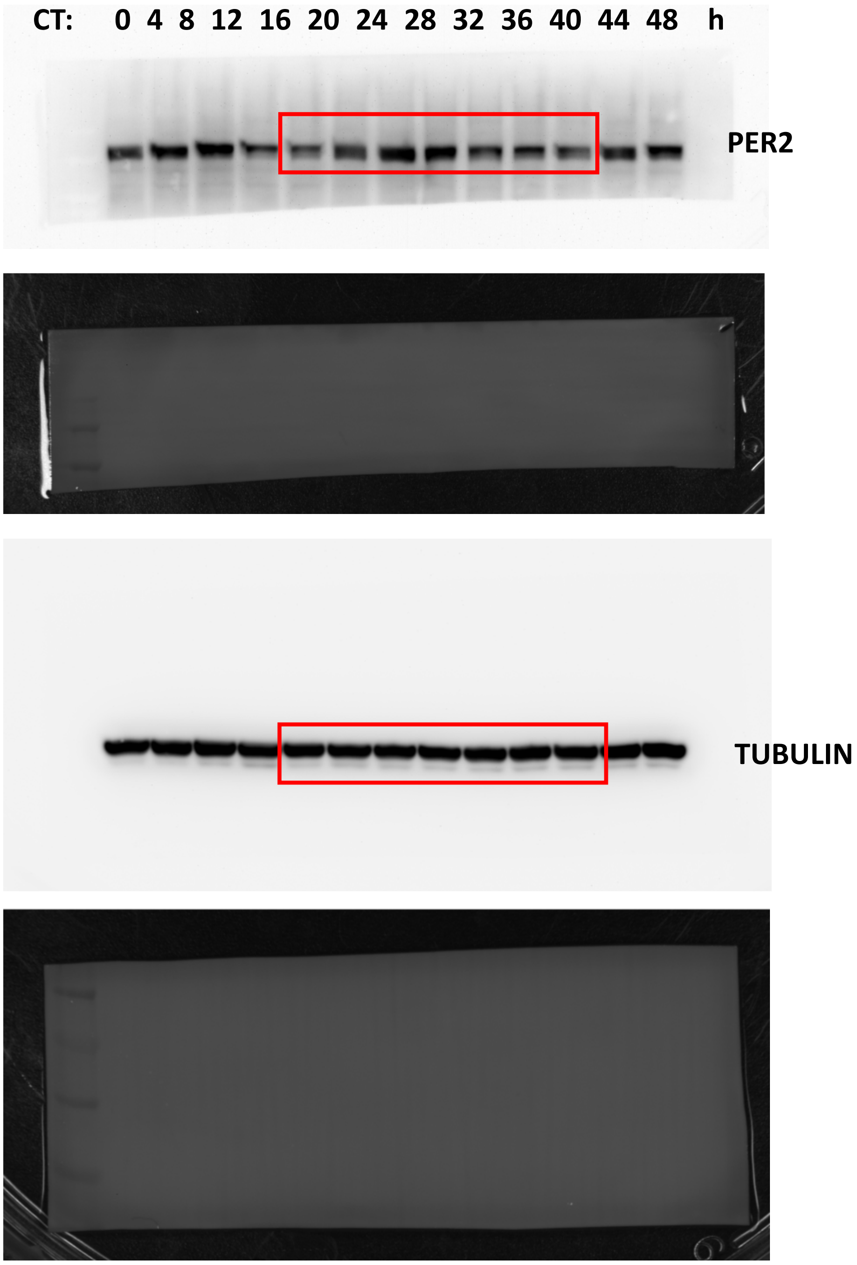

Figure 1c and 3a (blots for quantification)

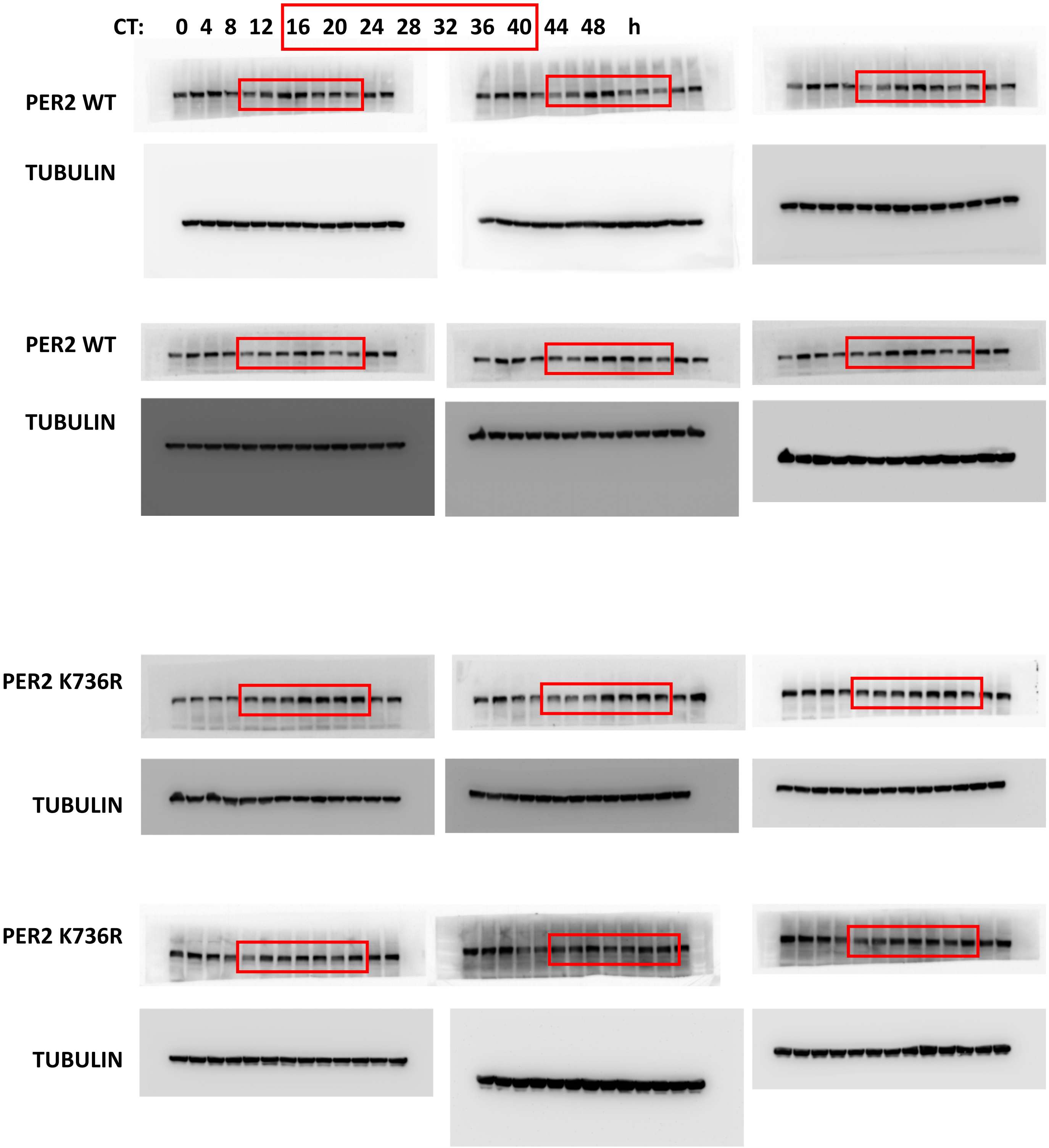

Figure 2a

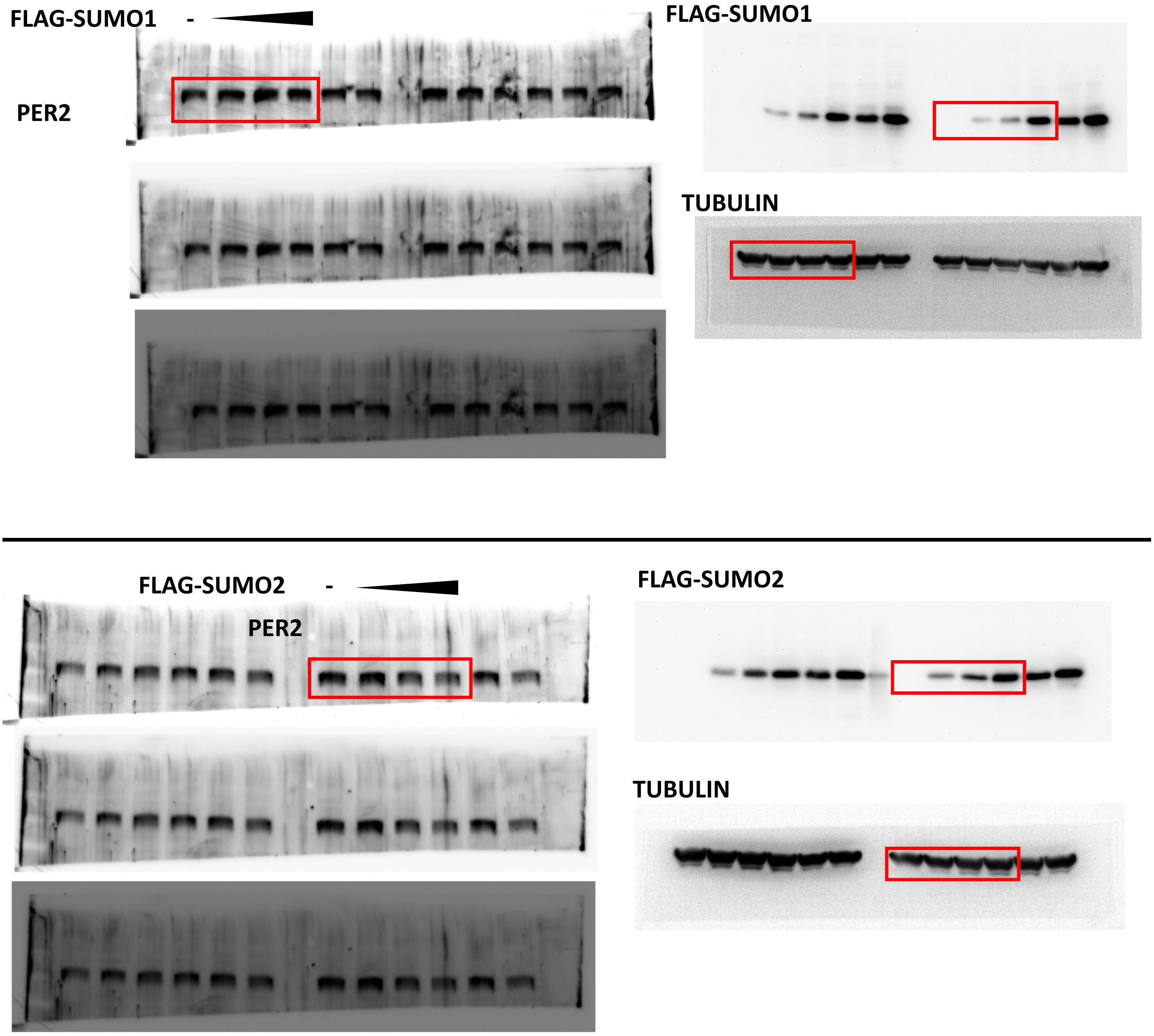
FLAG-SUMO1 -
PER2
FLAG-SUMO1
TUBULIN
FLAG-SUMO2 -
PER2
FLAG-SUMO2
TUBULIN

Figure 2a (blots for quantification)

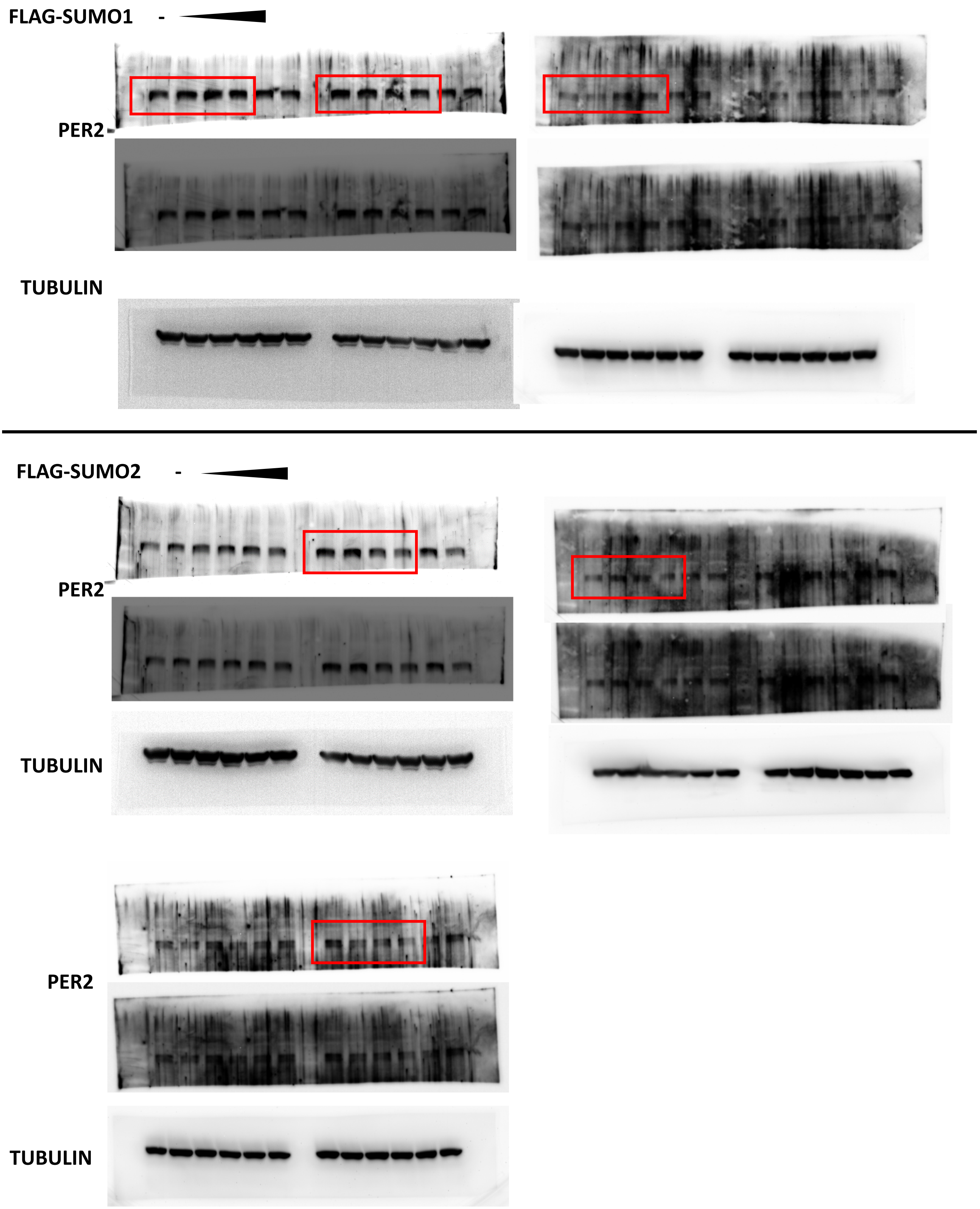

Figure 2b

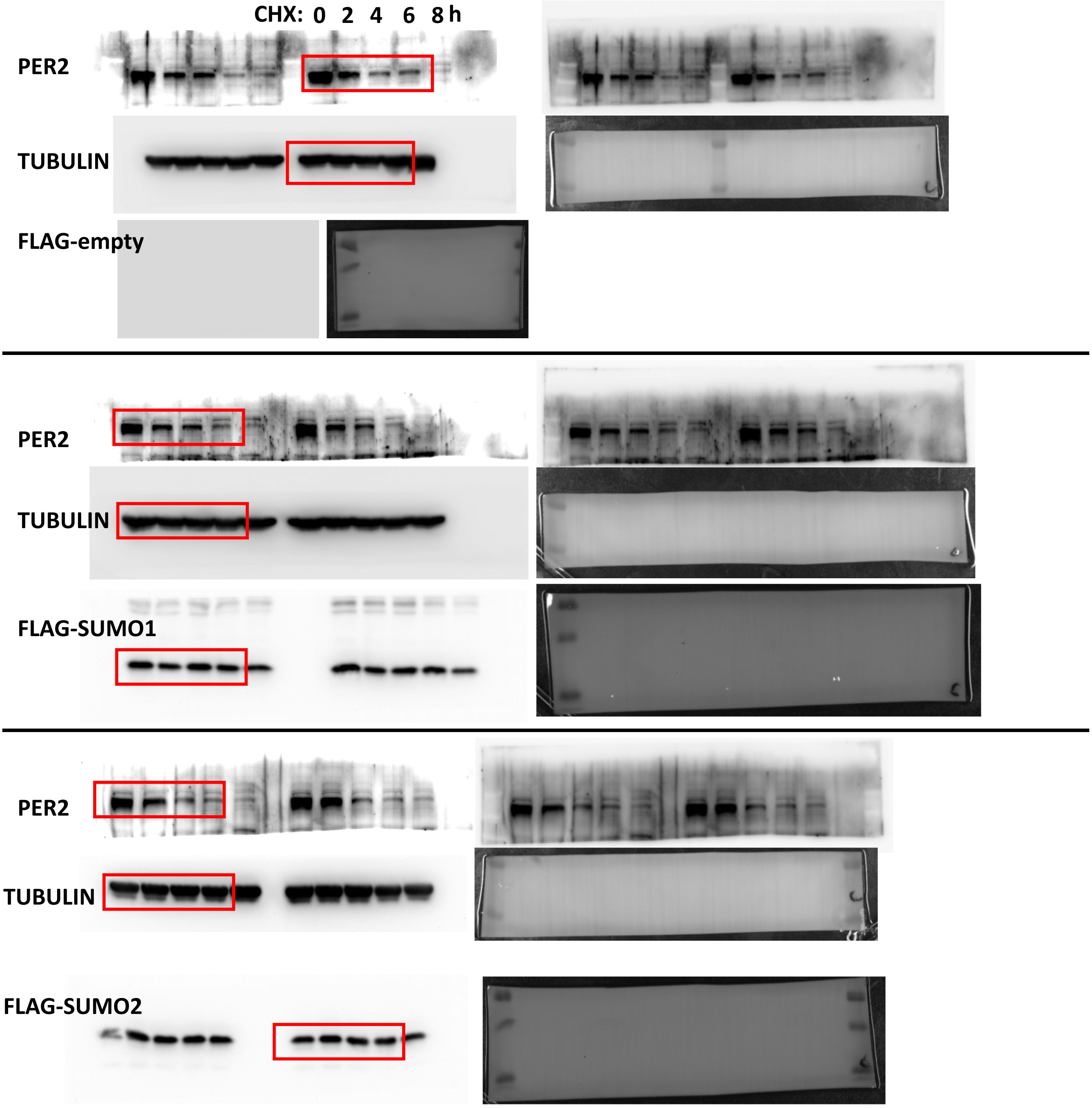
CHX: 0 2 4 6 8 h
PER2
TUBULIN
FLAG-empty
PER2
TUBULIN
FLAG-SUMO1
PER2
TUBULIN
FLAG-SUMO2

Figure 2b (blots for quantification)

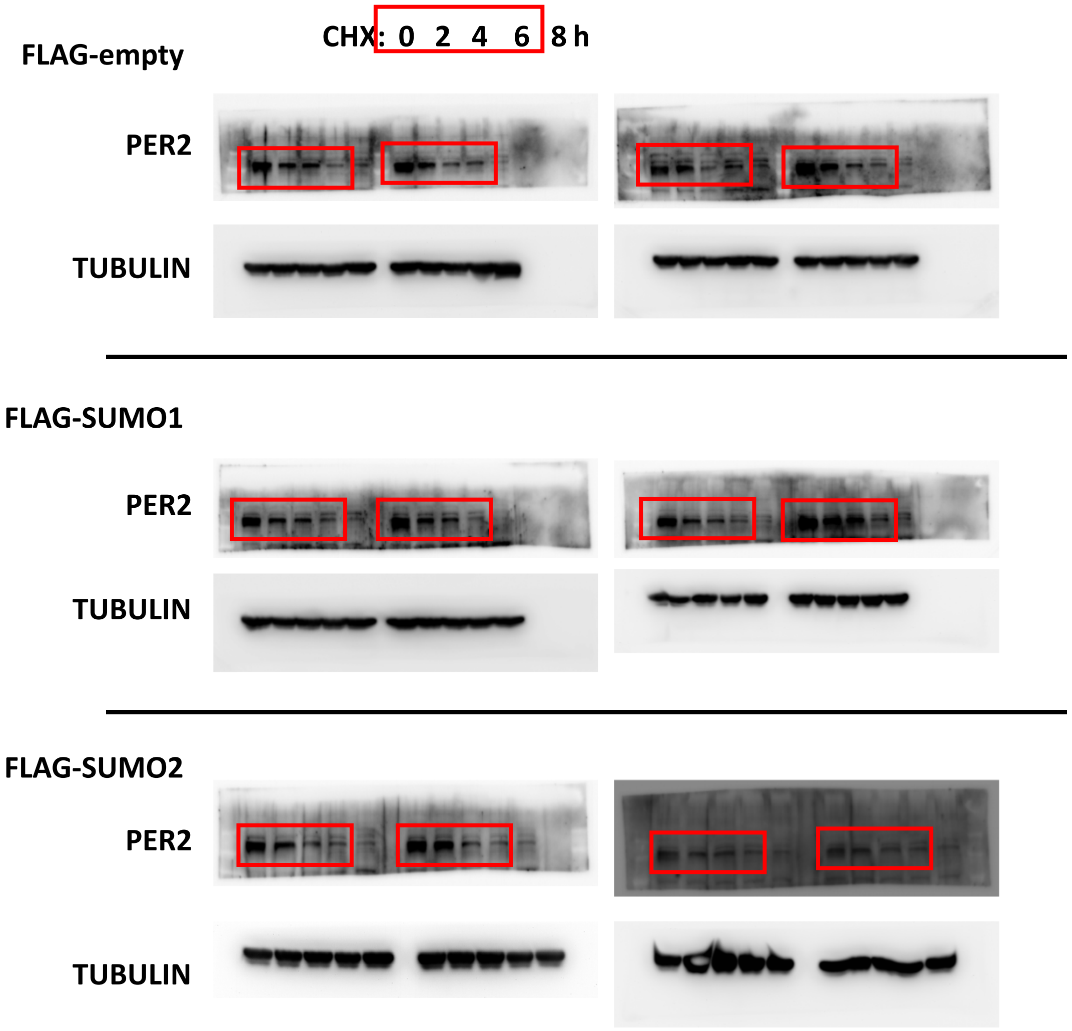

Figure 2c

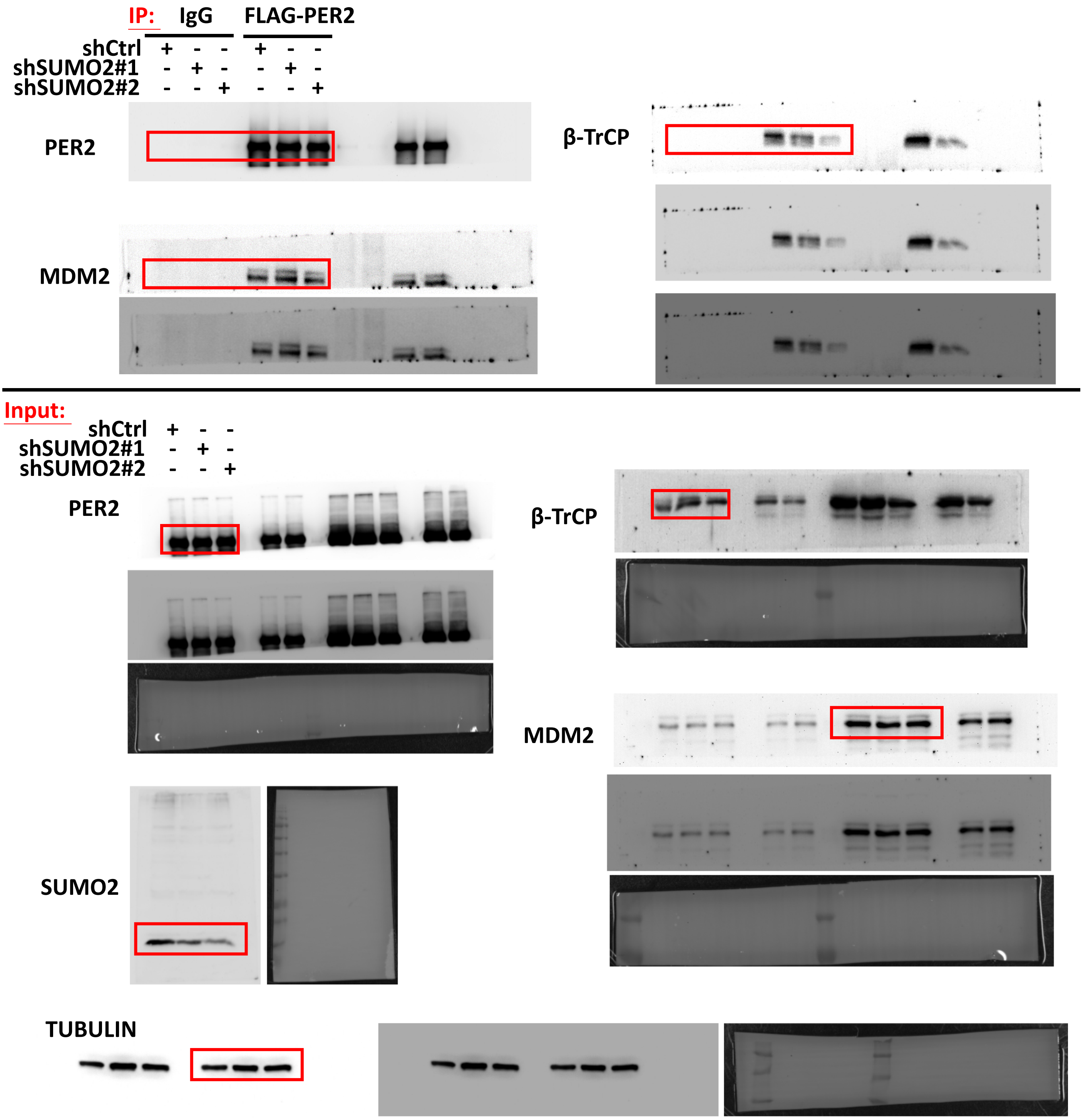
IP:
IgG
FLAG-PER2
shCtrl + - - + - -
shSUMO2#1 - + - - + -
shSUMO2#2 - - + - - +
PER2
β-TrCP
MDM2
Input:
shCtrl + - -
shSUMO2#1 - + -
shSUMO2#2 - - +
PER2
β-TrCP
MDM2
SUMO2
TUBULIN

Figure 2c (additional IP sample)

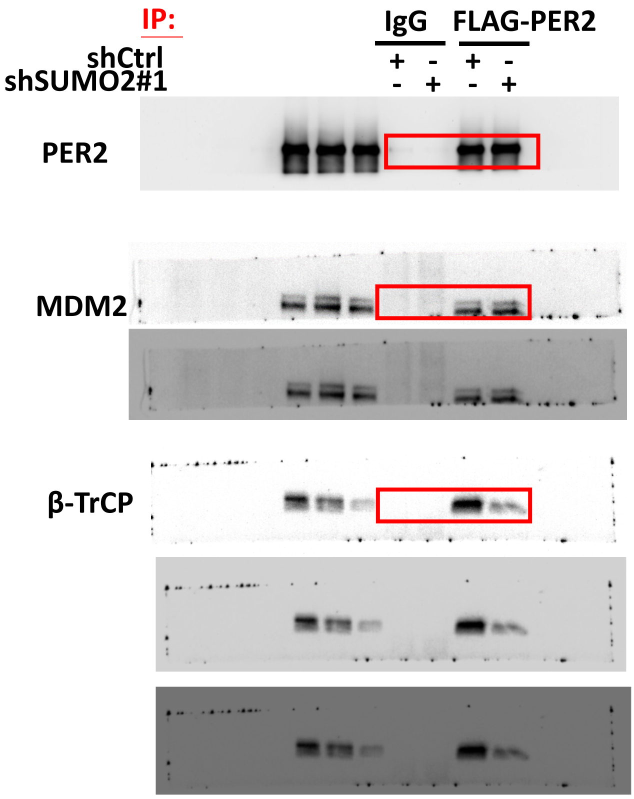

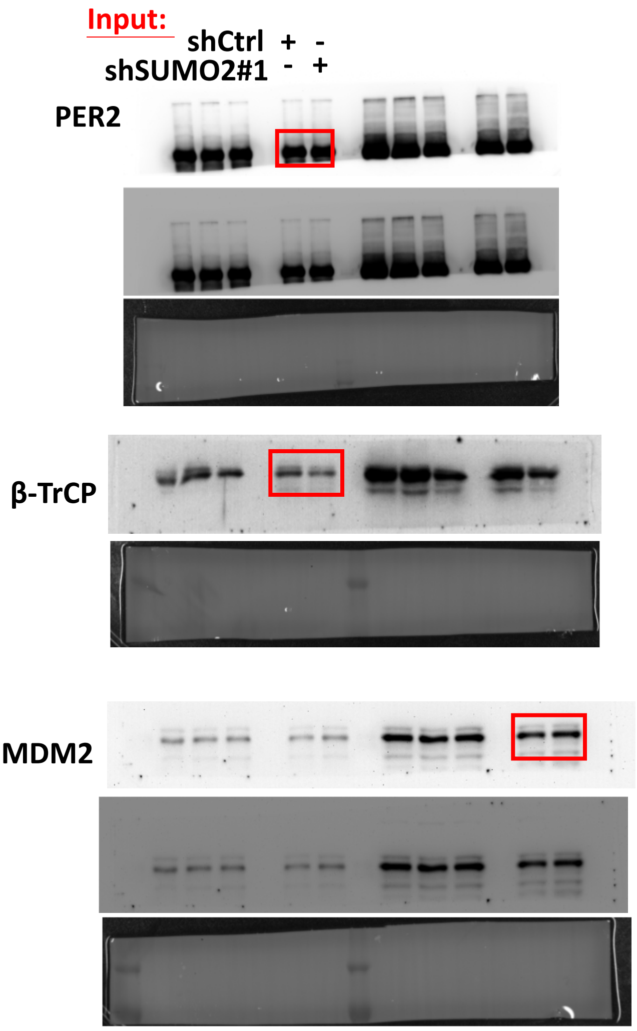

Figure 2f

Denatured IP:

PER2 WT + + -
PER2 K736R - - +
FLAG-SUMO1 - + +

PER2

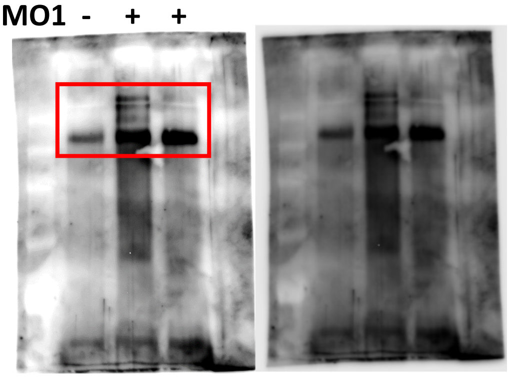

Input:

PER2

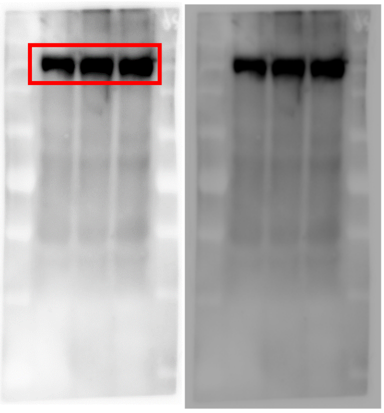

SUMO1

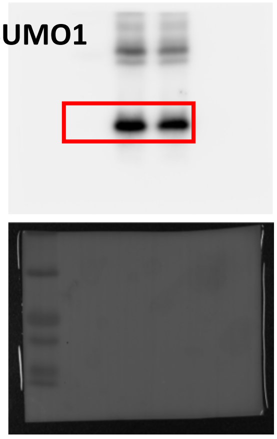

Figure 2g

Denatured IP:

PER2 WT + + -
PER2 K736R - - +
FLAG-SUMO2 - + +

PER2

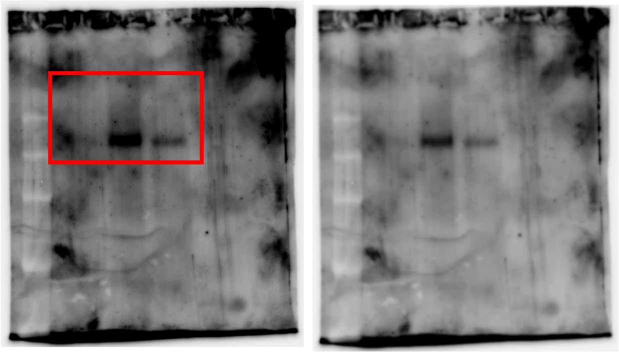

Input:

PER2

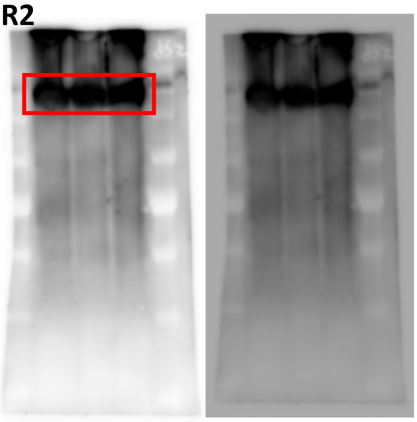

SUMO2

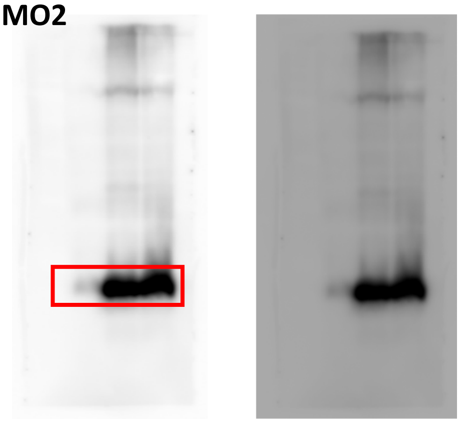

Figure 2h

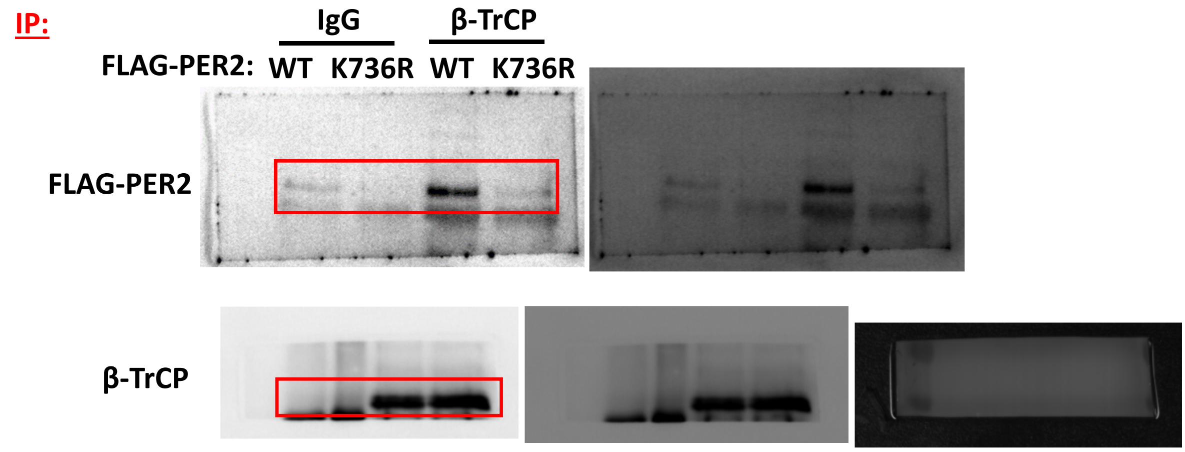
IP:
IgG
β-TrCP
FLAG-PER2:
WT
K736R
WT
K736R
FLAG-PER2
β-TrCP

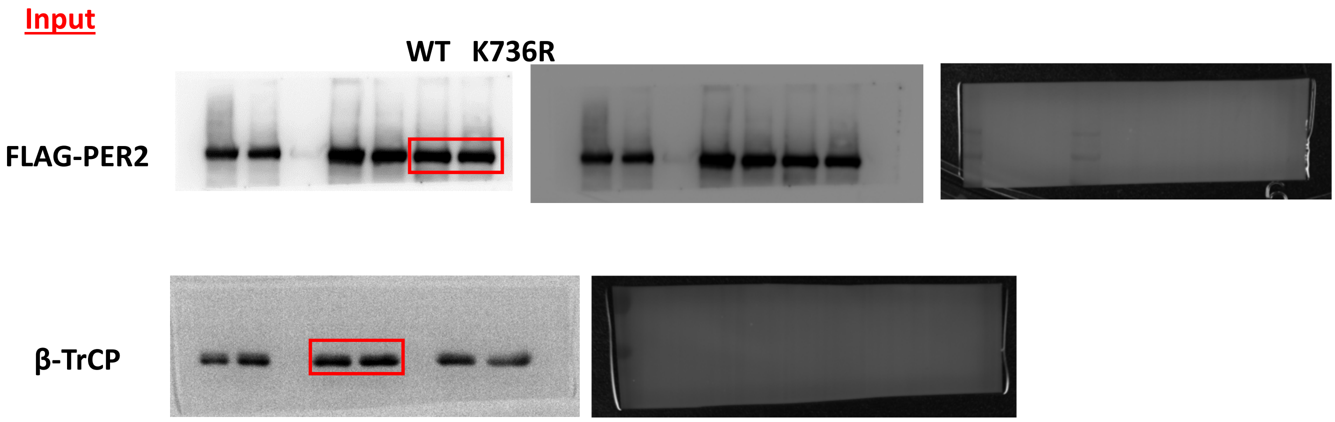
Input
WT
K736R
FLAG-PER2
β-TrCP

Figure 2f (additional experiments)

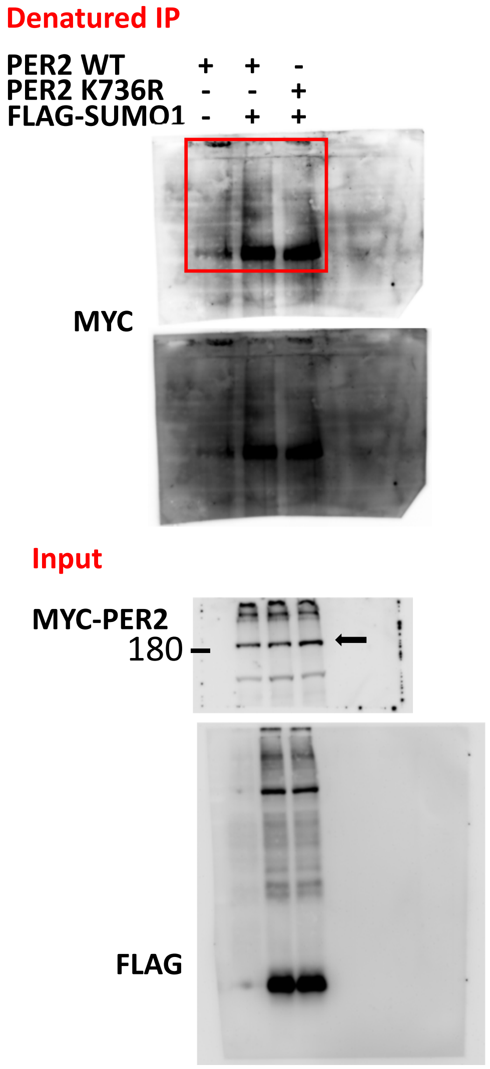

Figure 2h (additional experiments)

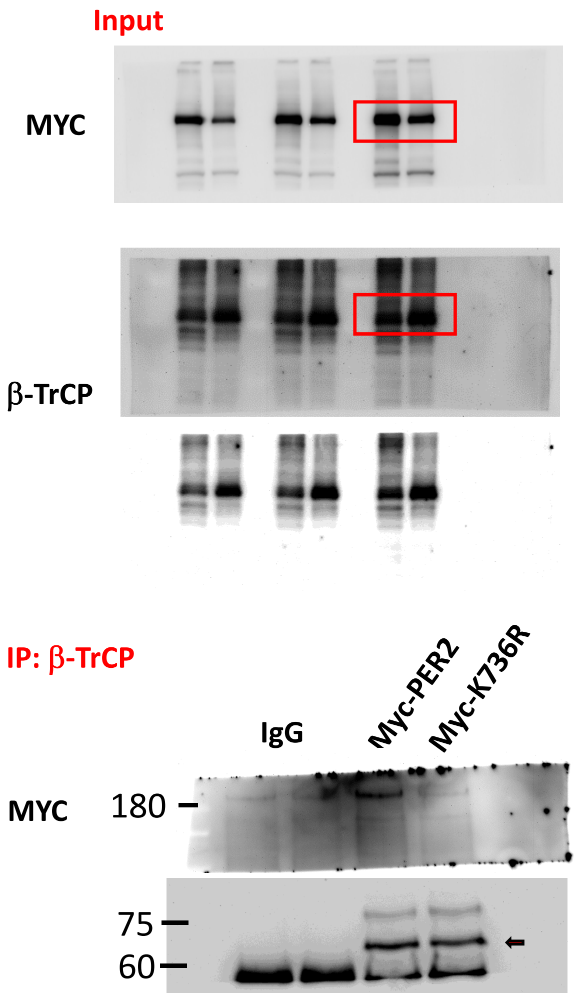

Figure 3a

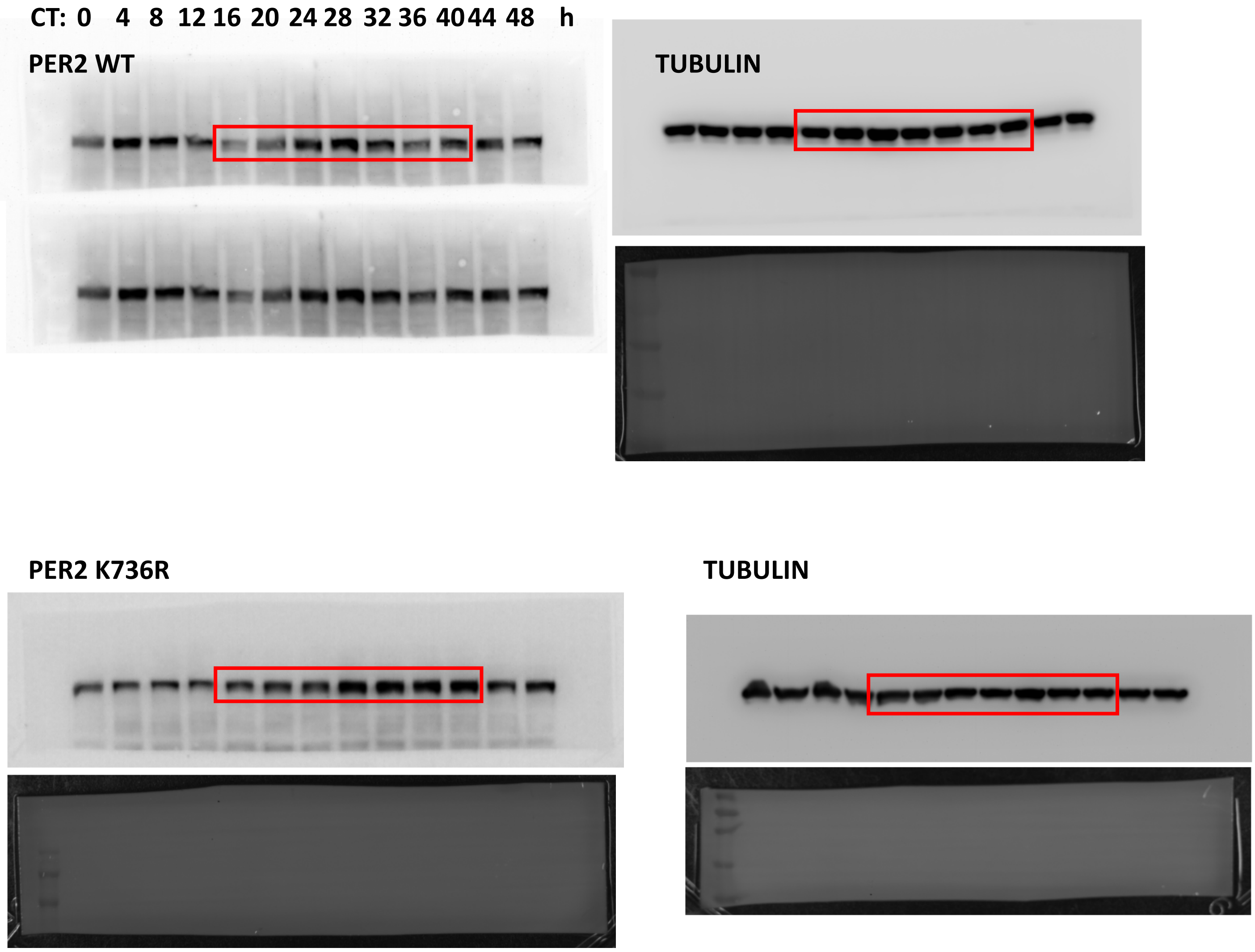

CT: 0 4 8 12 16 20 24 28 32 36 40 44 48 h
PER2 WT
TUBULIN
PER2 K736R
TUBULIN

Figure 4a

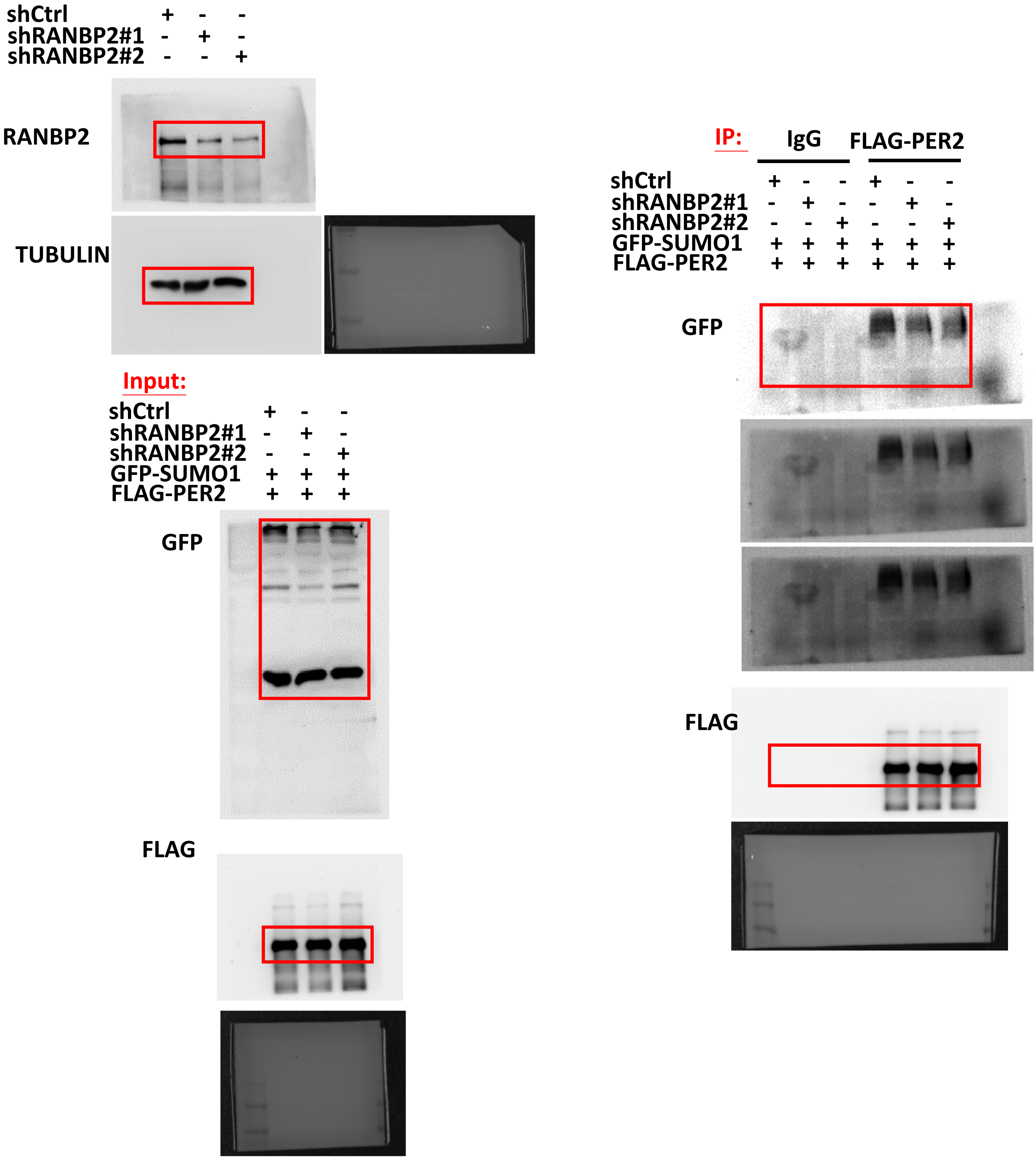

shCtrl + - -
shRANBP2#1 - + -
shRANBP2#2 - - +
RANBP2
TUBULIN
Input:
shCtrl + - -
shRANBP2#1 - + -
shRANBP2#2 - - +
GFP-SUMO1 + + +
FLAG-PER2 + + +
GFP
FLAG
IP:
IgG
FLAG-PER2
shCtrl + - - + - -
shRANBP2#1 - + - - + -
shRANBP2#2 - - + - - +
GFP-SUMO1 + + + + + +
FLAG-PER2 + + + + + +
GFP
FLAG

Figure 4b

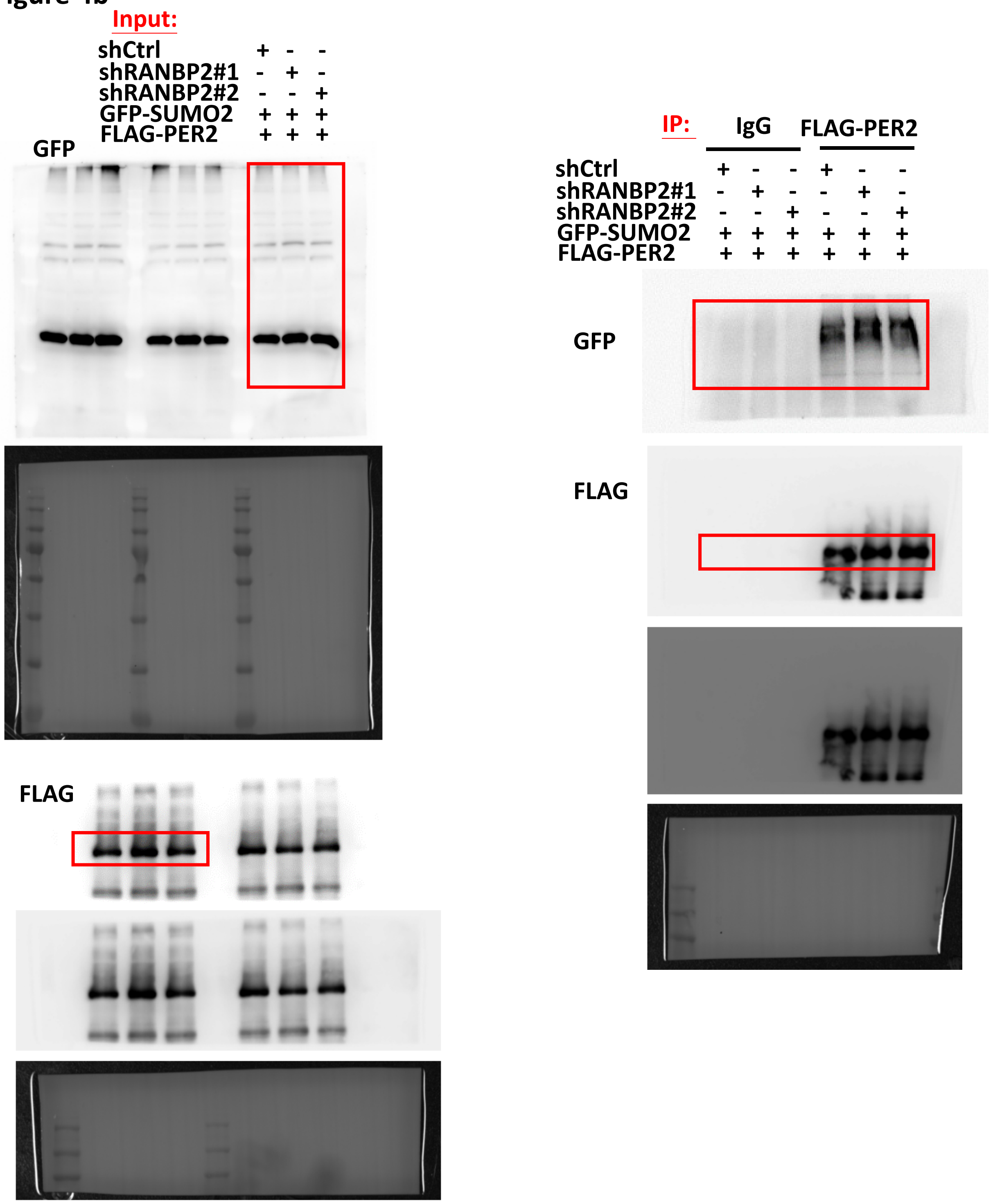
Input:
shCtrl + - -
shRANBP2#1 - + -
shRANBP2#2 - - +
GFP-SUMO2 + + +
FLAG-PER2 + + +
GFP
FLAG
IP: IgG FLAG-PER2
shCtrl + - - + - -
shRANBP2#1 - + - - + -
shRANBP2#2 - - + - - +
GFP-SUMO2 + + + + + +
FLAG-PER2 + + + + + +
GFP
FLAG

Figure 4a, 4b (additional experiments)

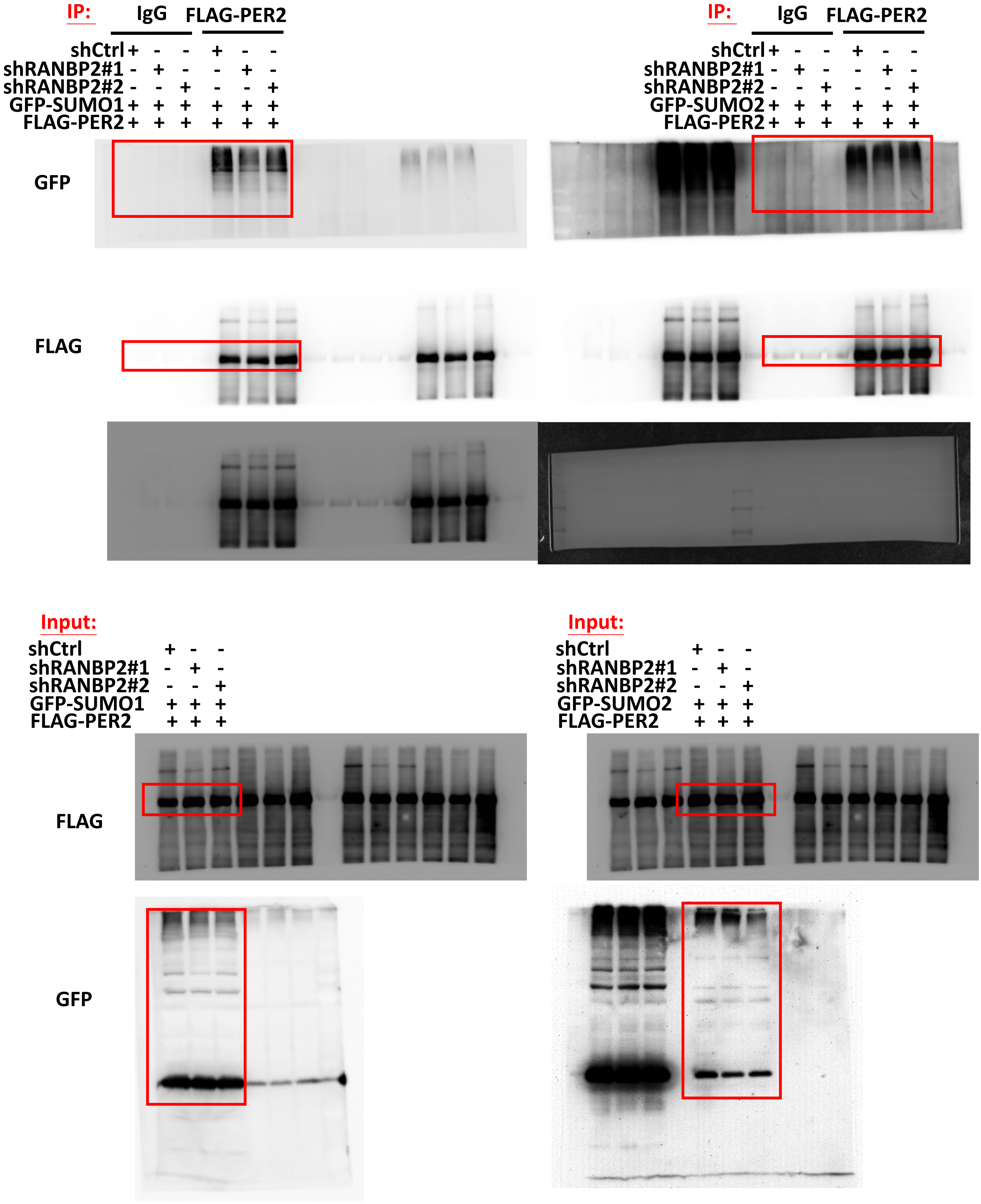

Figure 4c

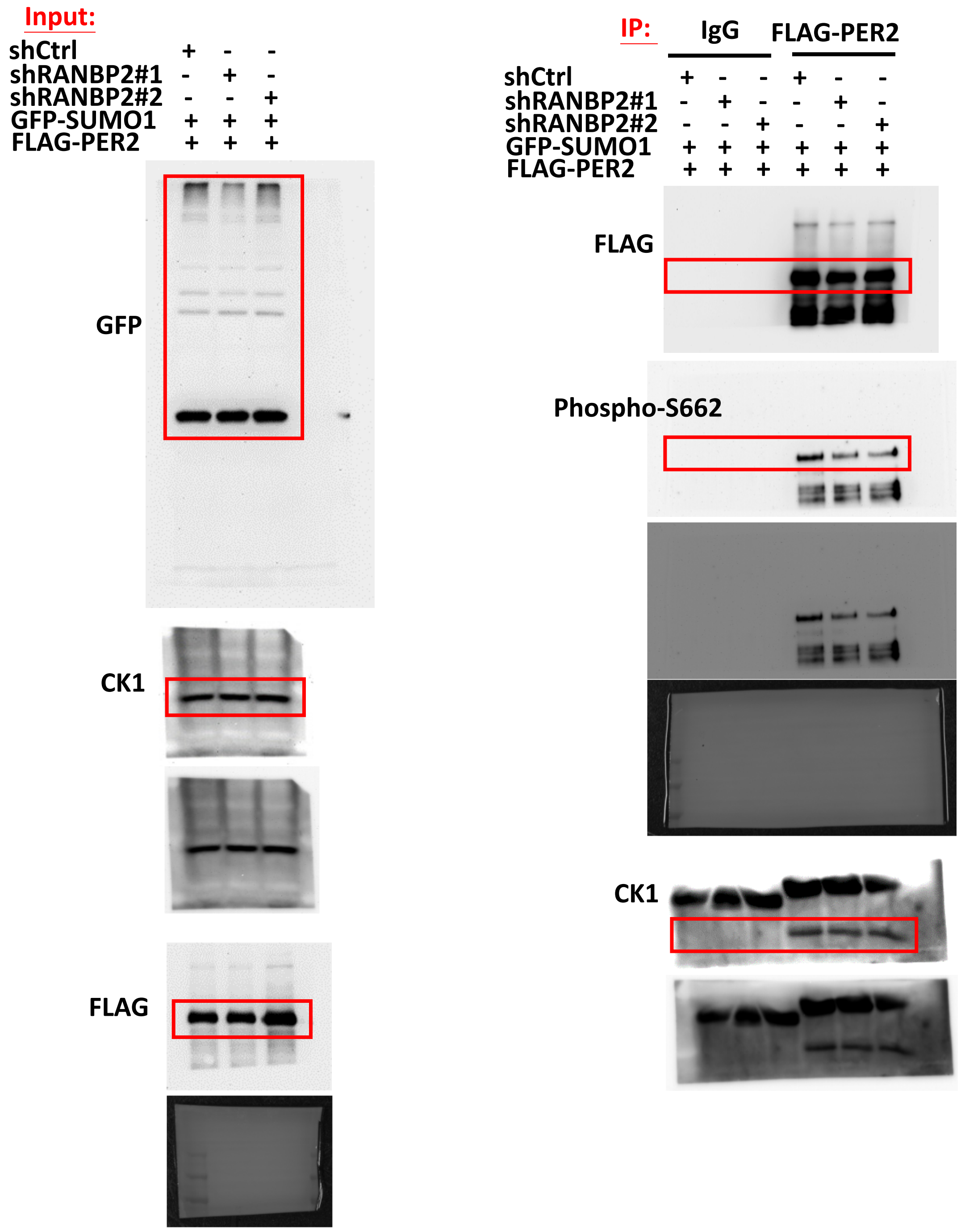
Input:
shCtrl + - -
shRANBP2#1 - + -
shRANBP2#2 - - +
GFP-SUMO1 + + +
FLAG-PER2 + + +
GFP
CK1
FLAG
IP:
IgG
FLAG-PER2
shCtrl + - - + - -
shRANBP2#1 - + - - + -
shRANBP2#2 - - + - - +
GFP-SUMO1 + + + + + +
FLAG-PER2 + + + + + +
FLAG
Phospho-S662
CK1

Figure 4c (blots for quantification)

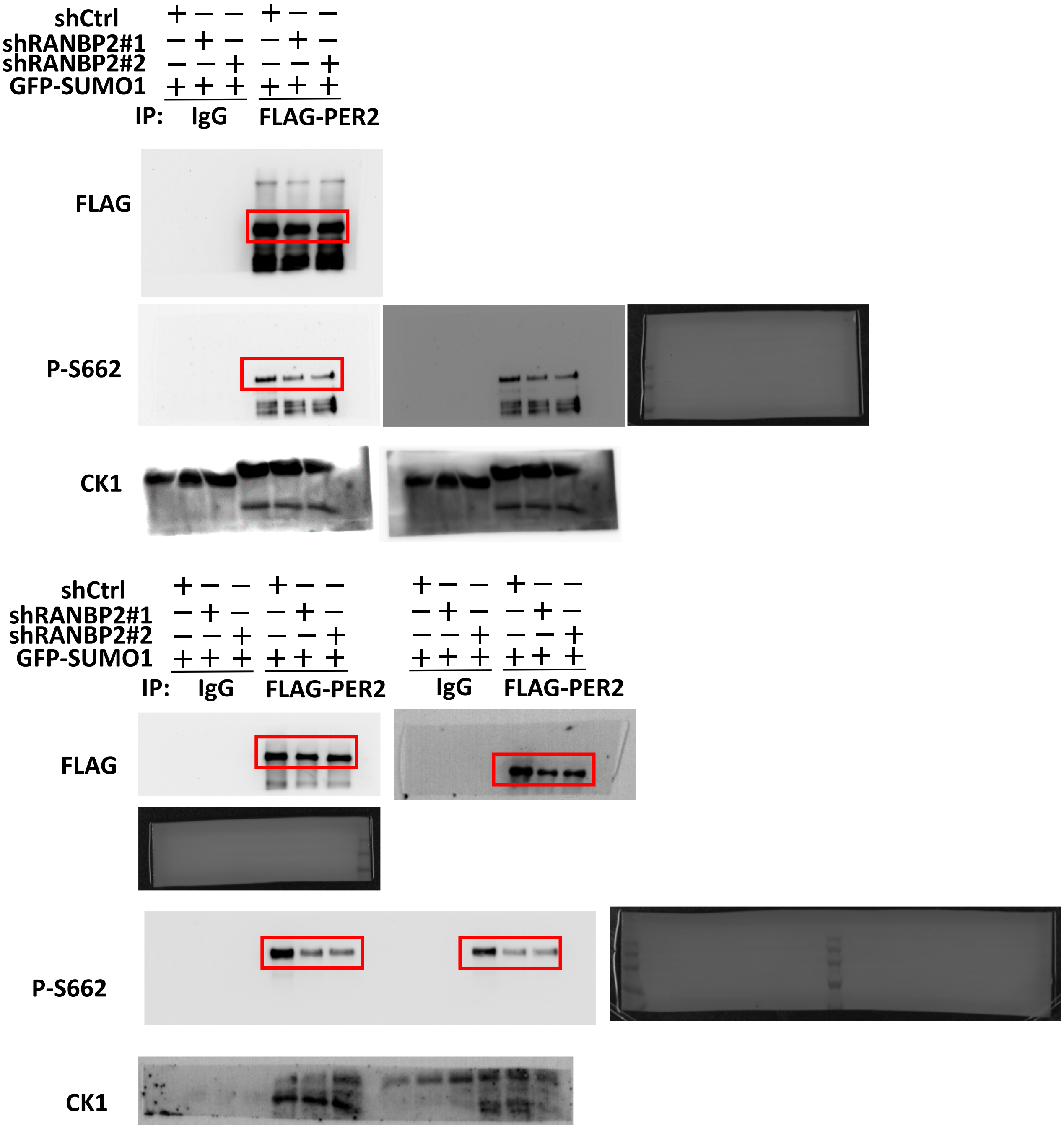

Figure 4d

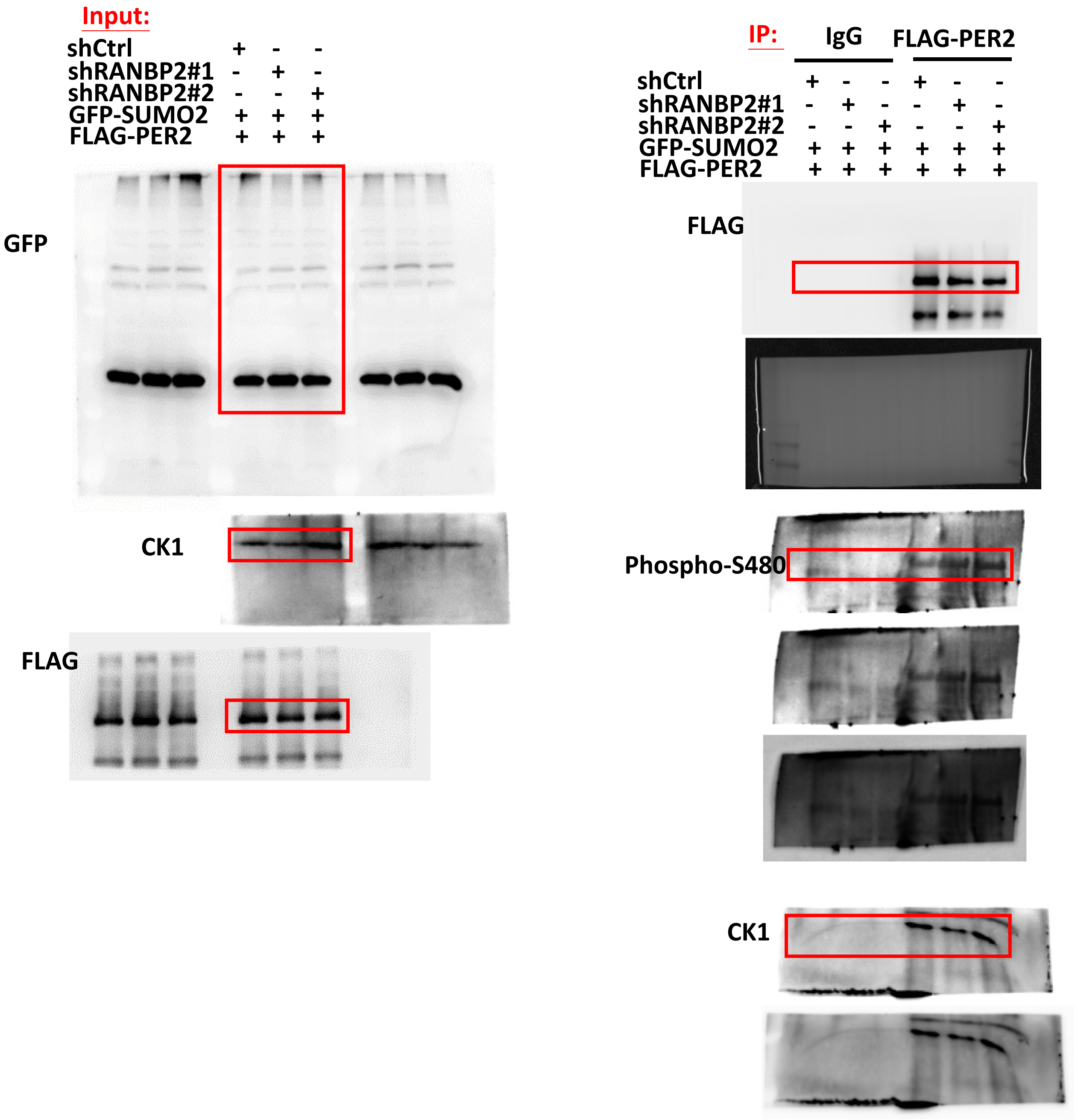

Input:
shCtrl + - -
shRANBP2#1 - + -
shRANBP2#2 - - +
GFP-SUMO2 + + +
FLAG-PER2 + + +
GFP
CK1
FLAG
IP:
IgG
FLAG-PER2
shCtrl + - - + - -
shRANBP2#1 - + - - + -
shRANBP2#2 - - + - - +
GFP-SUMO2 + + + + + +
FLAG-PER2 + + + + + +
FLAG
Phospho-S480
CK1

Figure 4d (blots for quantification)

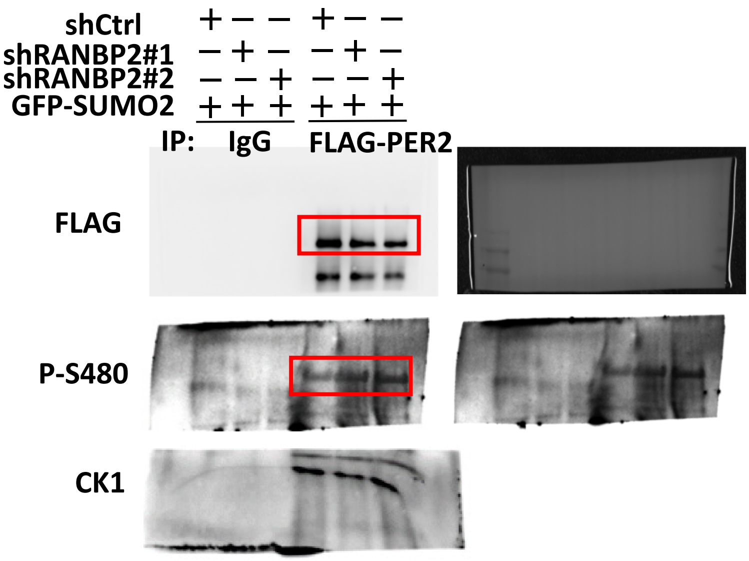

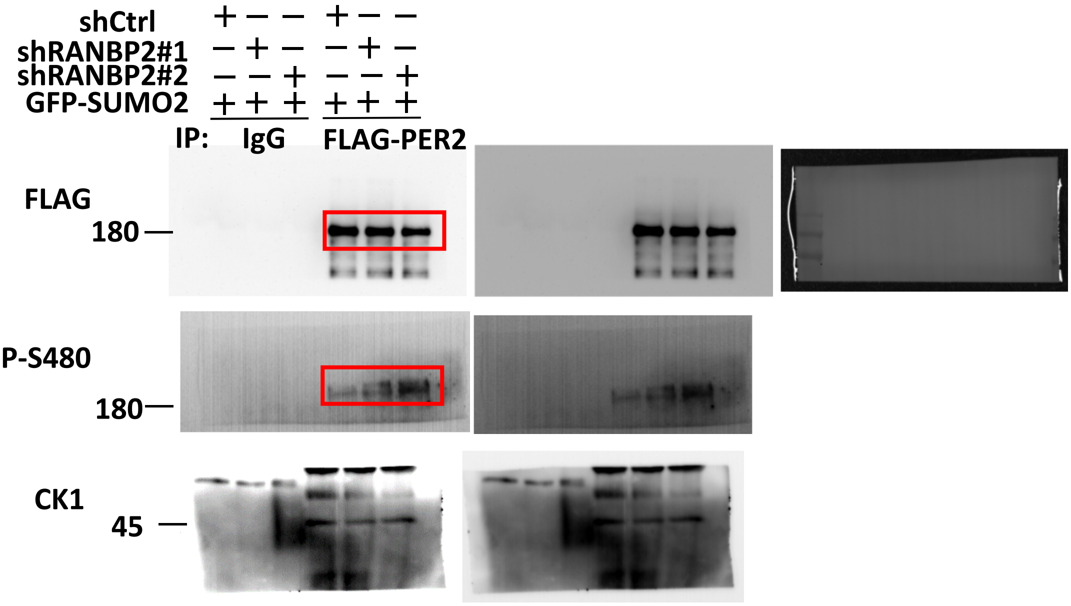

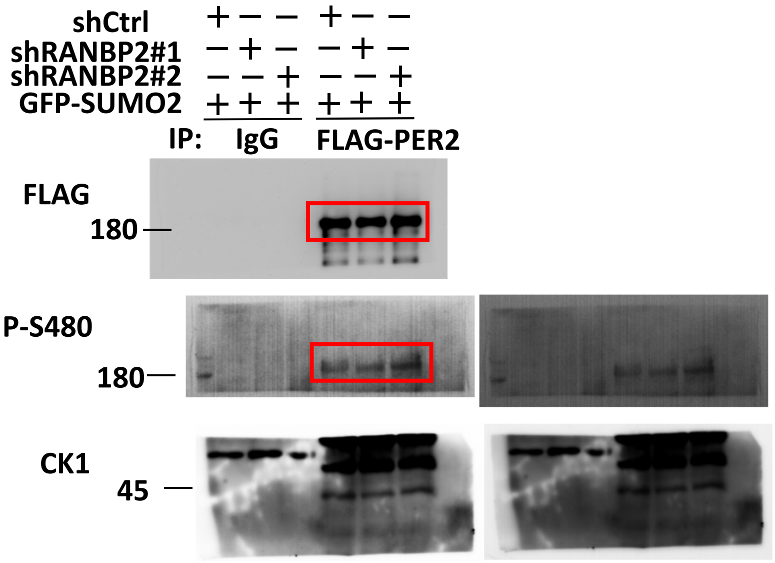

Figure 4e

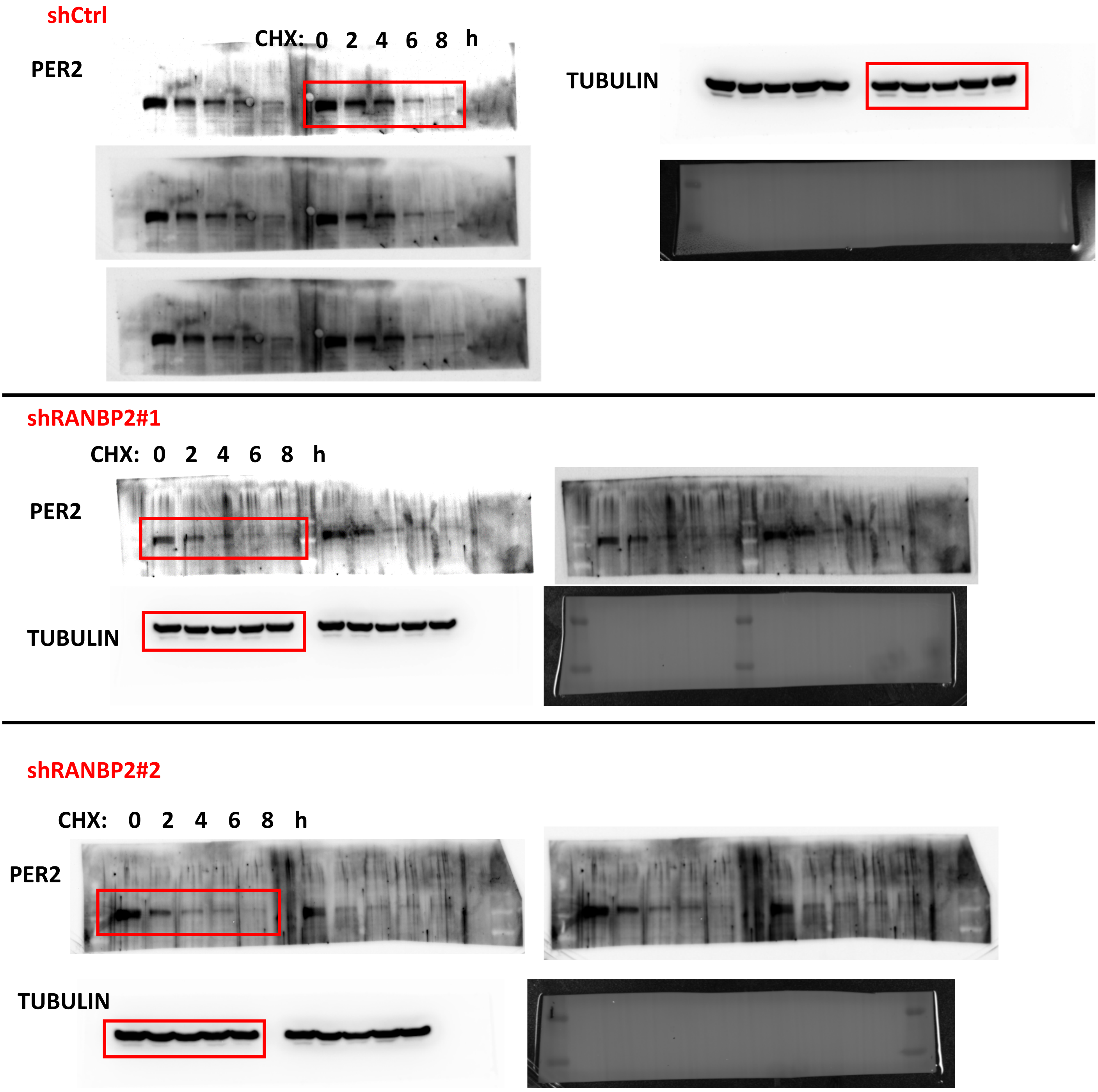
shCtrl
CHX: 0 2 4 6 8 h
PER2
TUBULIN
shRANBP2#1
CHX: 0 2 4 6 8 h
PER2
TUBULIN
shRANBP2#2
CHX: 0 2 4 6 8 h
PER2
TUBULIN

Figure 4e (blots for quantification)

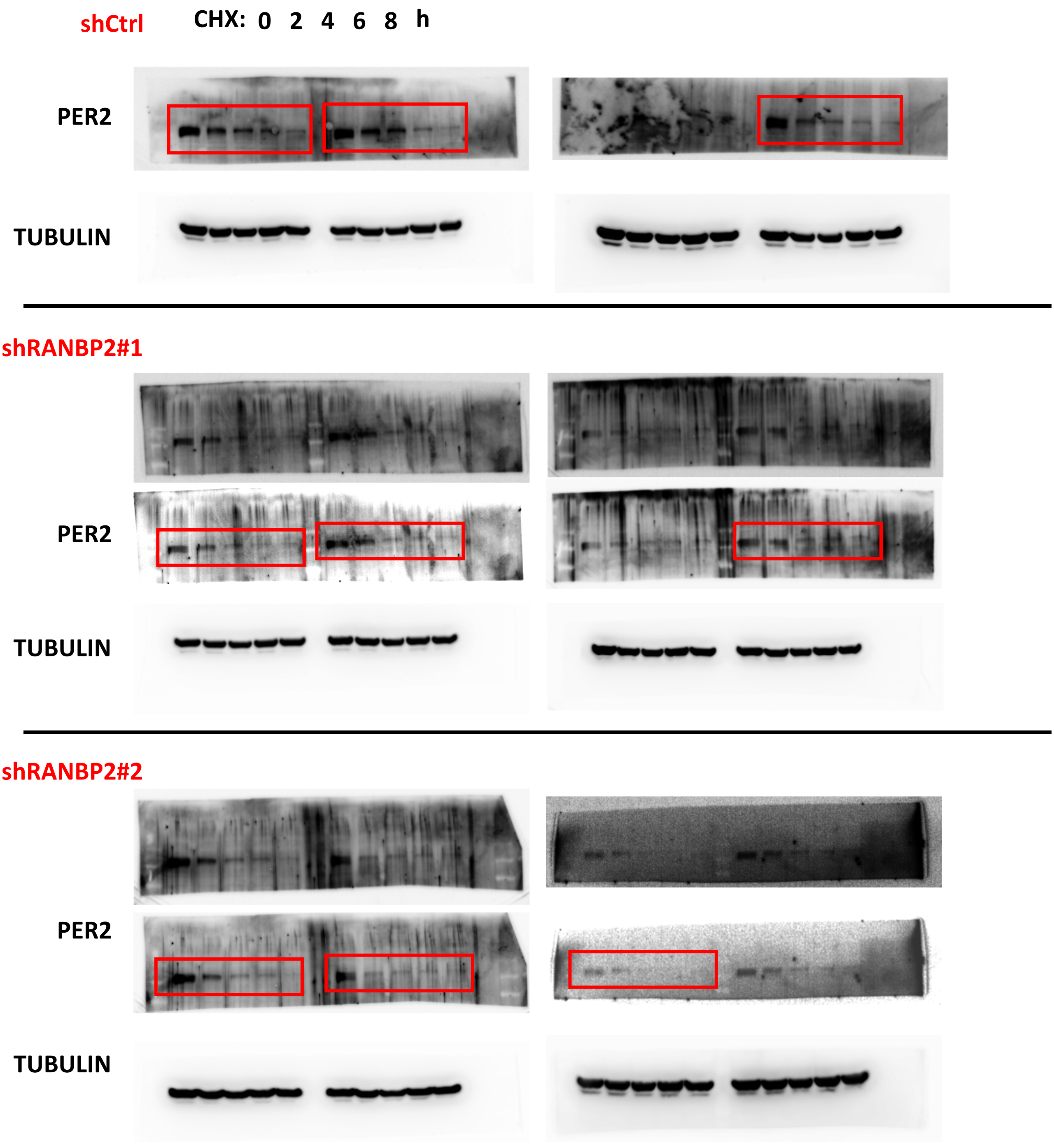

Figure S1a

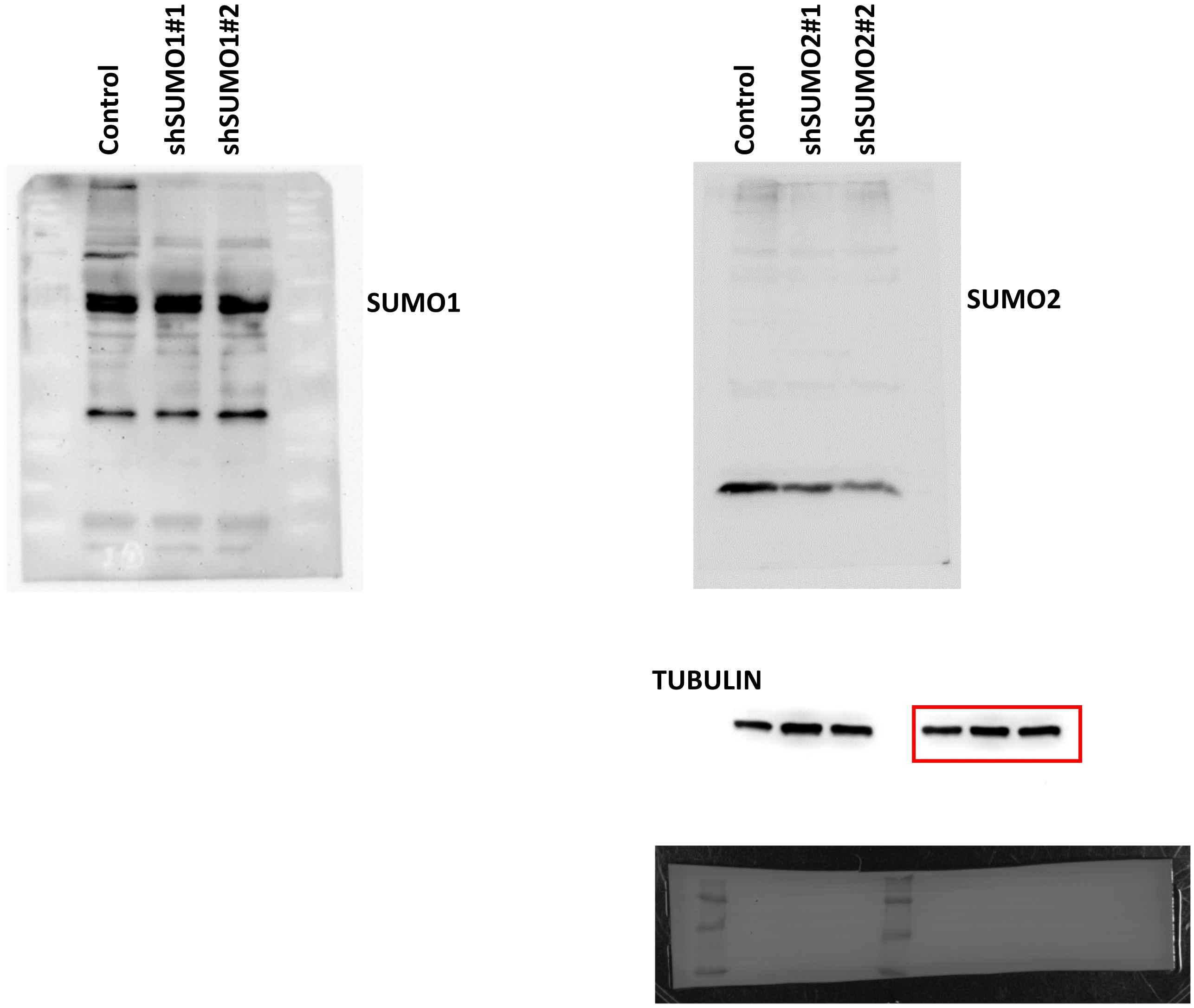

Control
shSUMO1#1
shSUMO1#2
SUMO1
Control
shSUMO2#1
shSUMO2#2
SUMO2
TUBULIN

Figure S1b

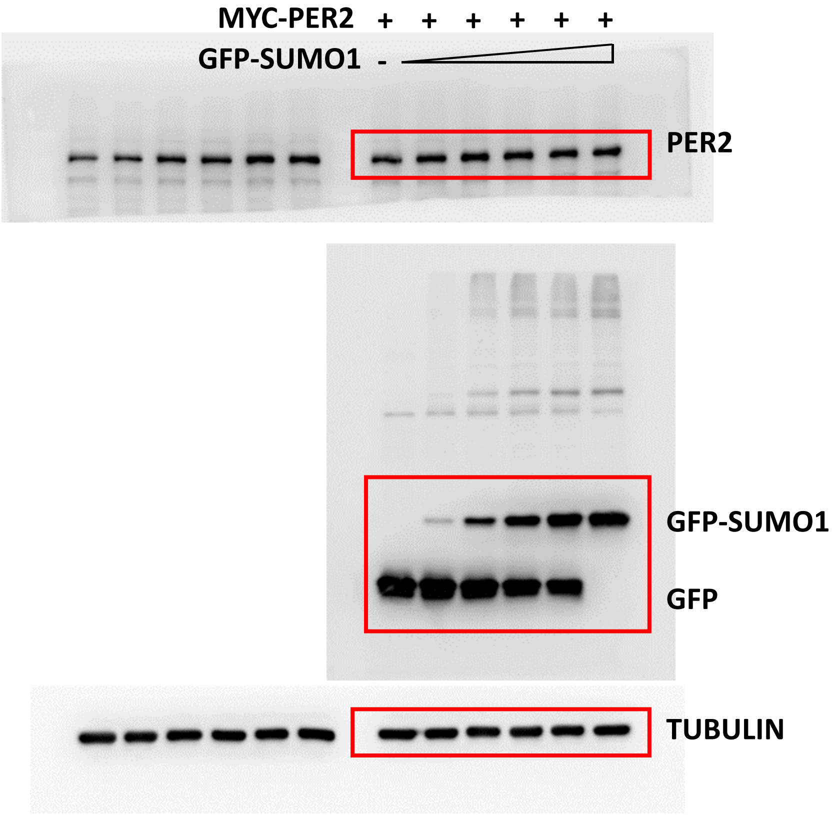
MYC-PER2 + + + + + +
GFP-SUMO1 -
PER2
GFP-SUMO1
GFP
TUBULIN

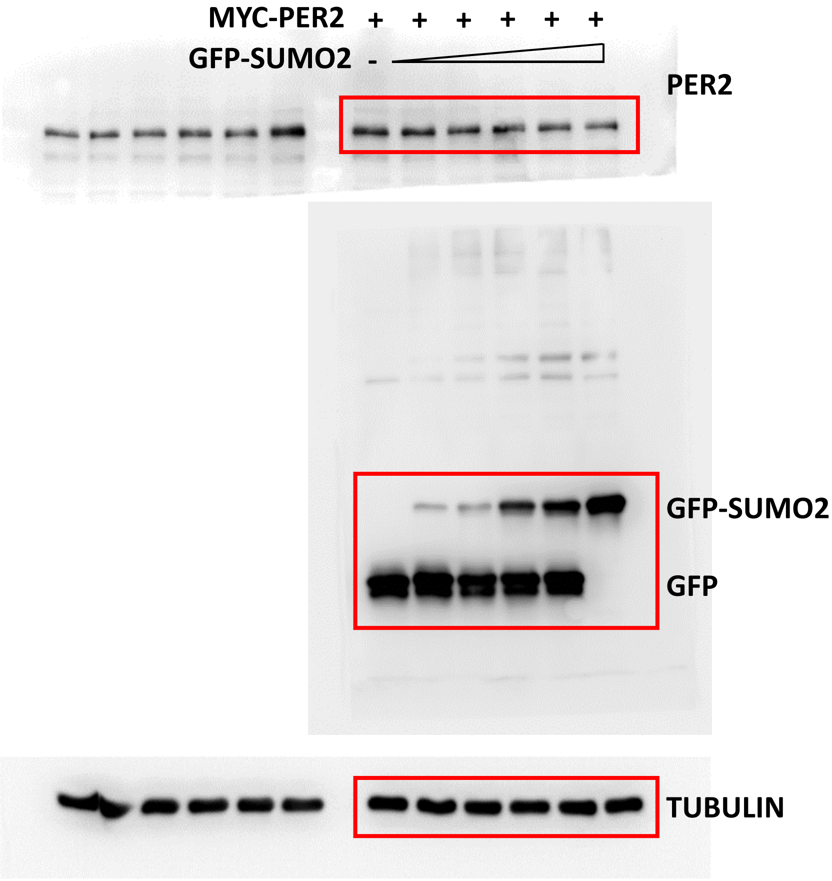
MYC-PER2 + + + + + +
GFP-SUMO2 -
PER2
GFP-SUMO2
GFP
TUBULIN

Figure S1b(replicate experiments)

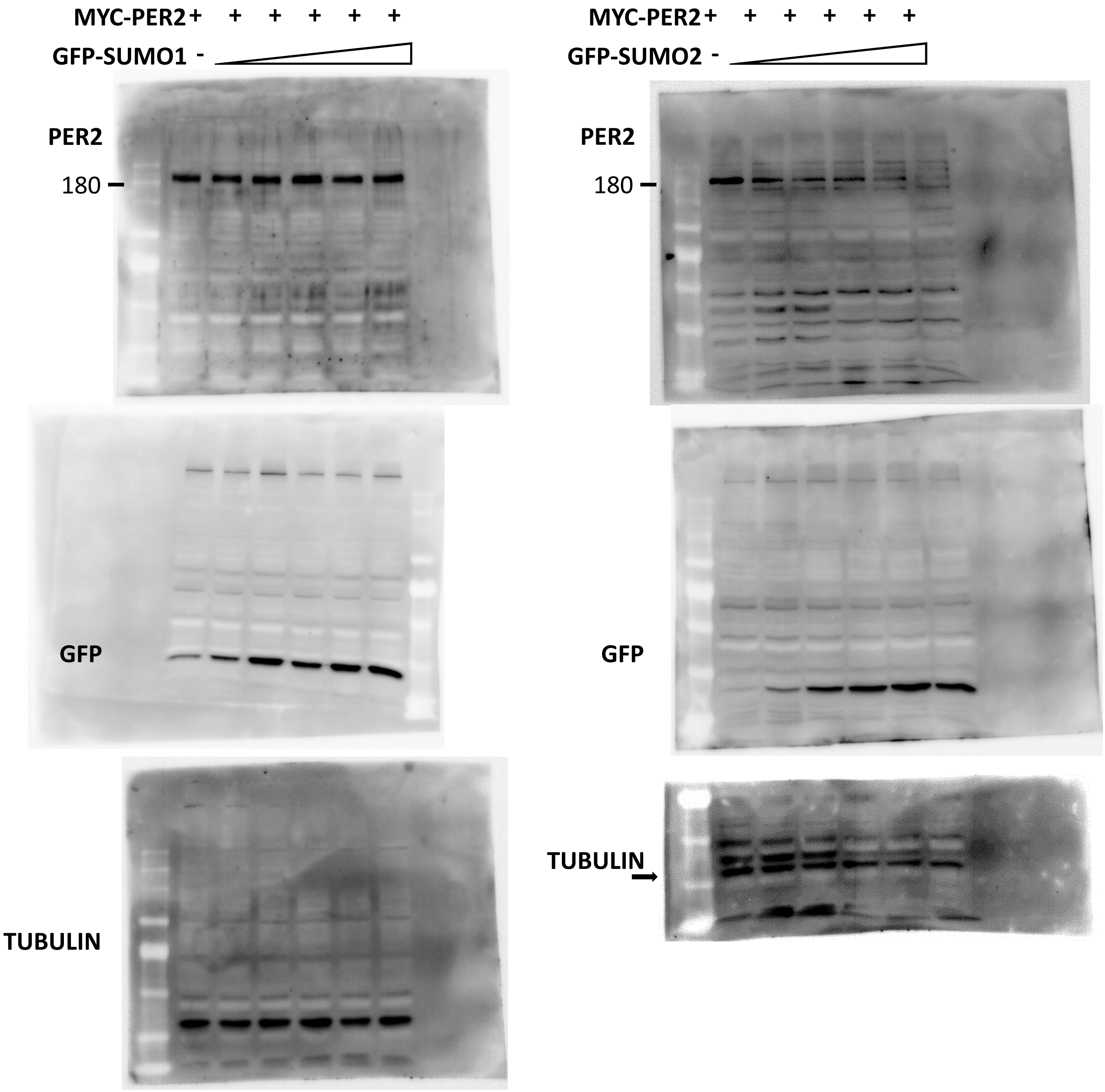

Figure S1c

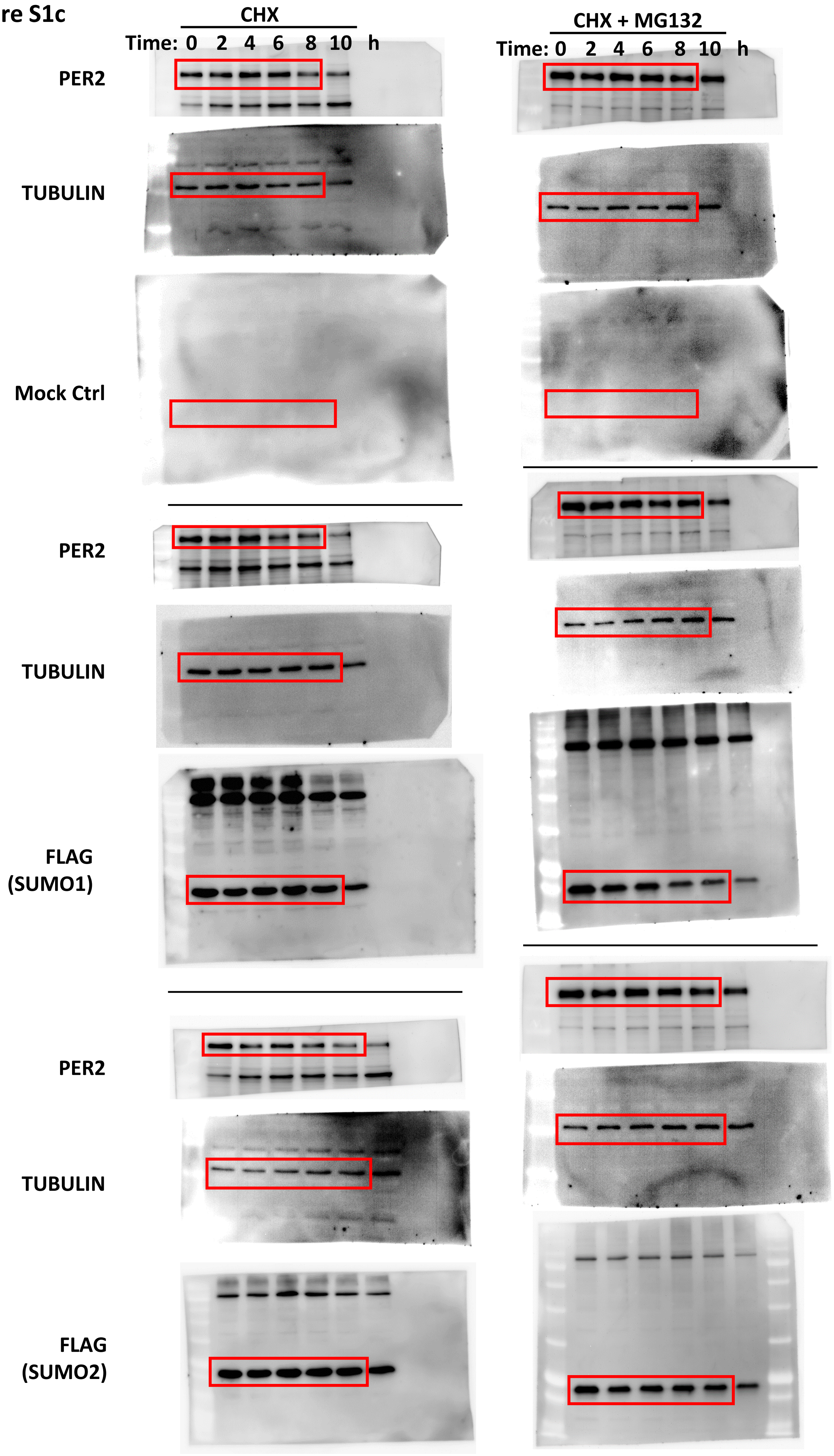

CHX
Time: 0 2 4 6 8 10 h
CHX + MG132
Time: 0 2 4 6 8 10 h
PER2
TUBULIN
Mock Ctrl
PER2
TUBULIN
FLAG
(SUMO1)
PER2
TUBULIN
FLAG
(SUMO2)

Figure S1c (blots for quantification)

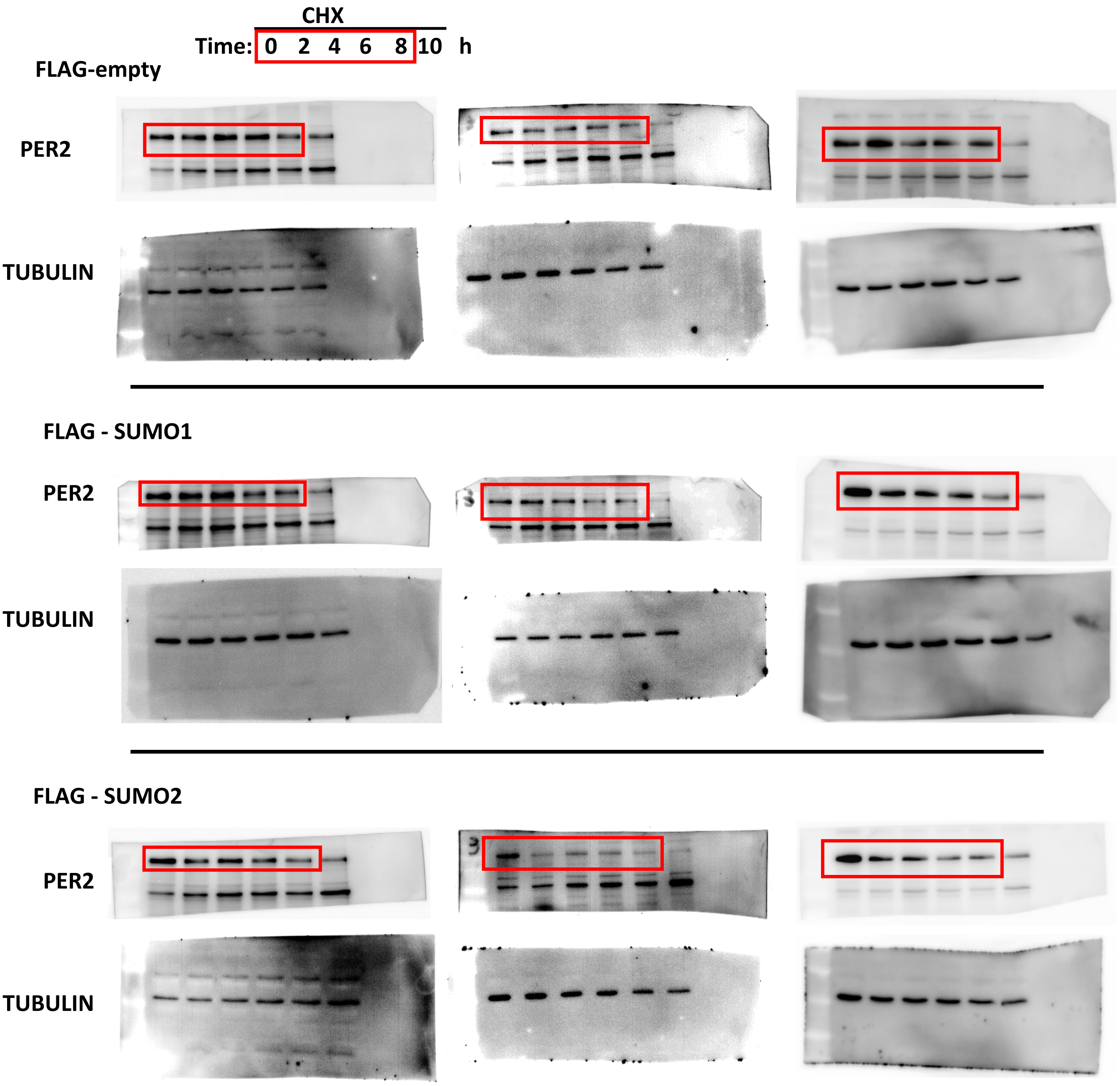

Figure S1d

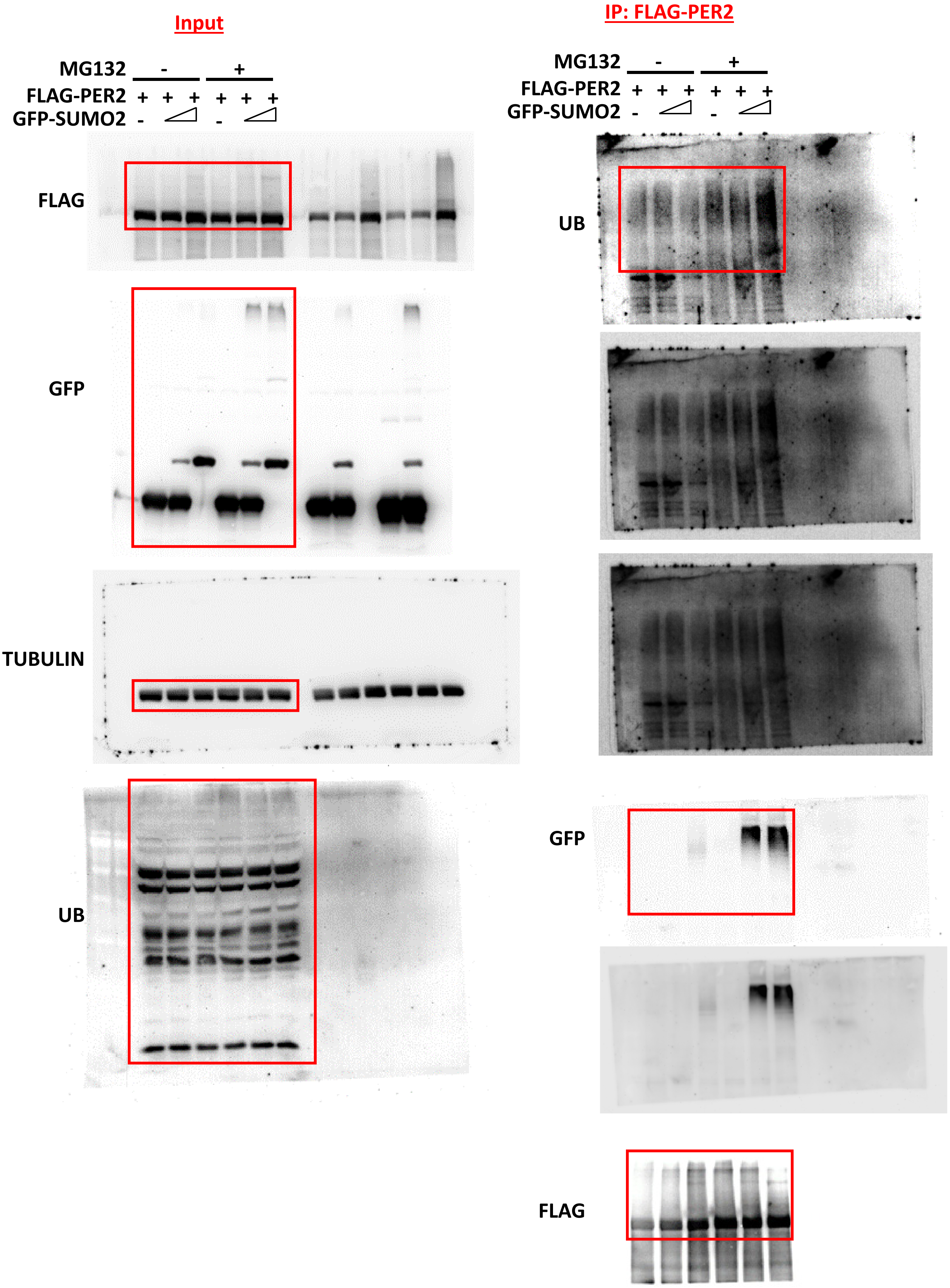

Input
MG132 - +
FLAG-PER2 + + + + + +
GFP-SUMO2 - -
FLAG
GFP
TUBULIN
UB
IP: FLAG-PER2
MG132 - +
FLAG-PER2 + + + + + +
GFP-SUMO2 - -
UB
GFP
FLAG

Figure S1d (additional experiments)

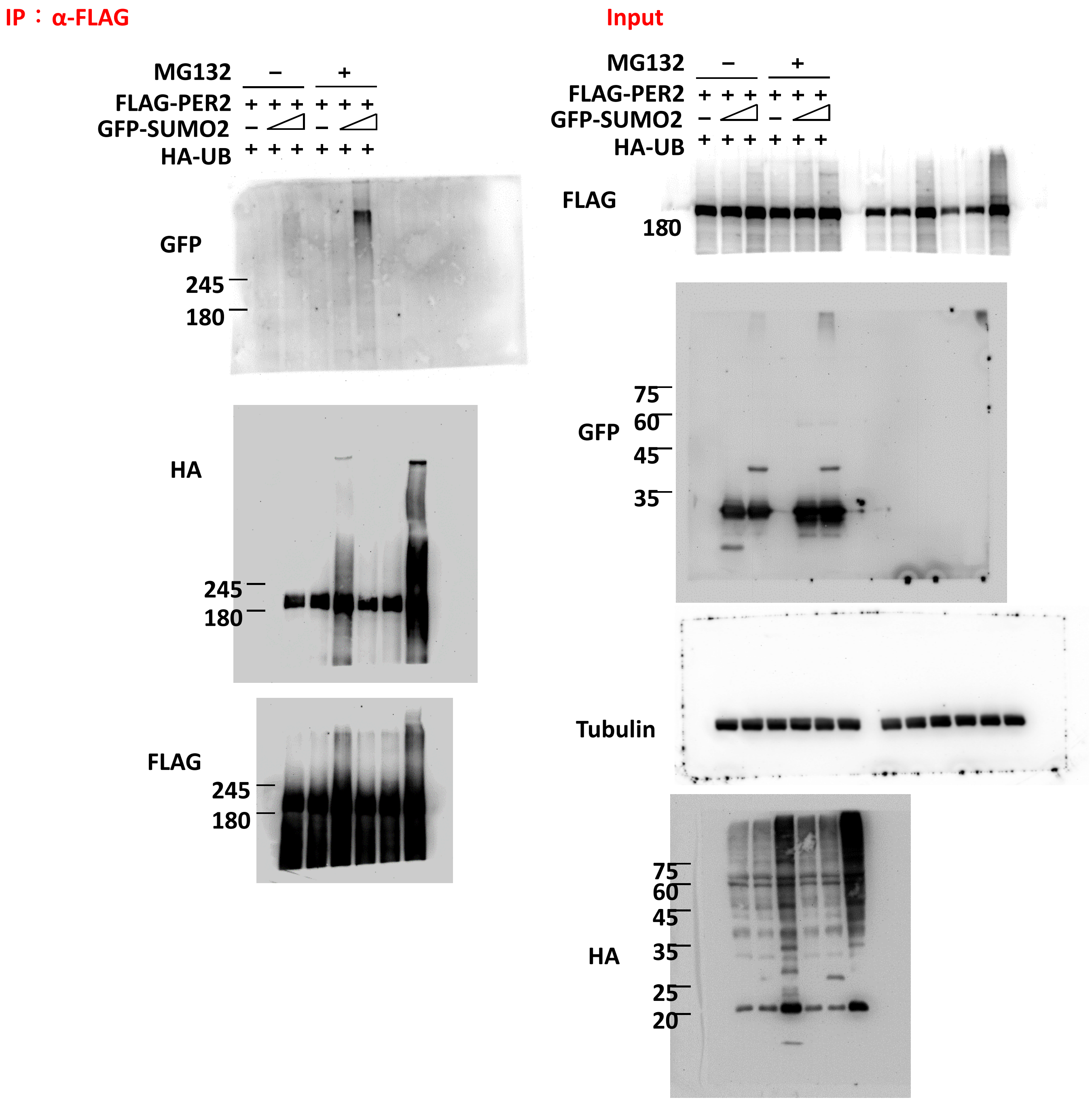

Figure S2a

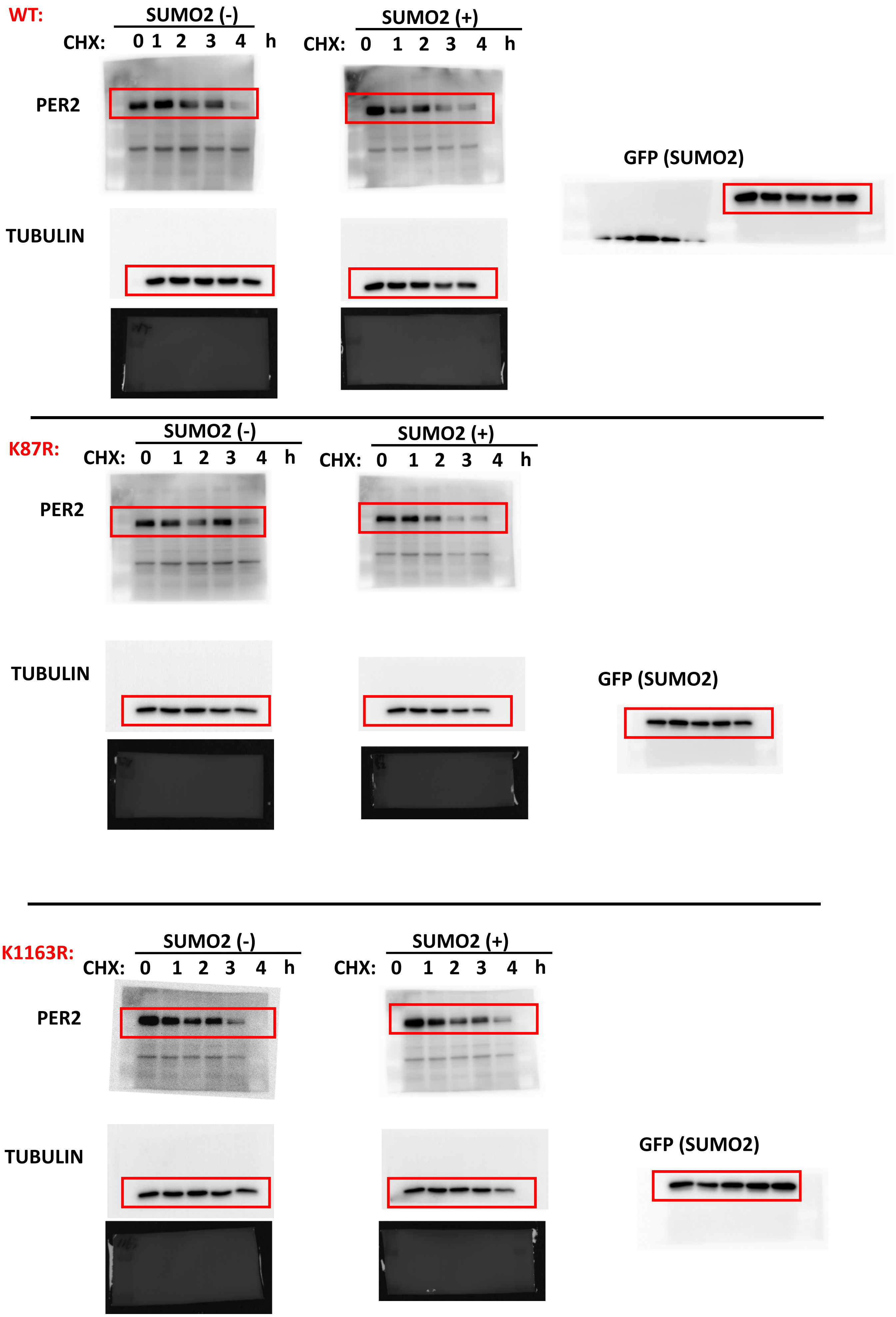
WT:
SUMO2 (-)
CHX: 0 1 2 3 4 h
SUMO2 (+)
CHX: 0 1 2 3 4 h
PER2
TUBULIN
GFP (SUMO2)
K87R:
SUMO2 (-)
CHX: 0 1 2 3 4 h
SUMO2 (+)
CHX: 0 1 2 3 4 h
PER2
TUBULIN
GFP (SUMO2)
K1163R:
SUMO2 (-)
CHX: 0 1 2 3 4 h
SUMO2 (+)
CHX: 0 1 2 3 4 h
PER2
TUBULIN
GFP (SUMO2)

Figure S2a

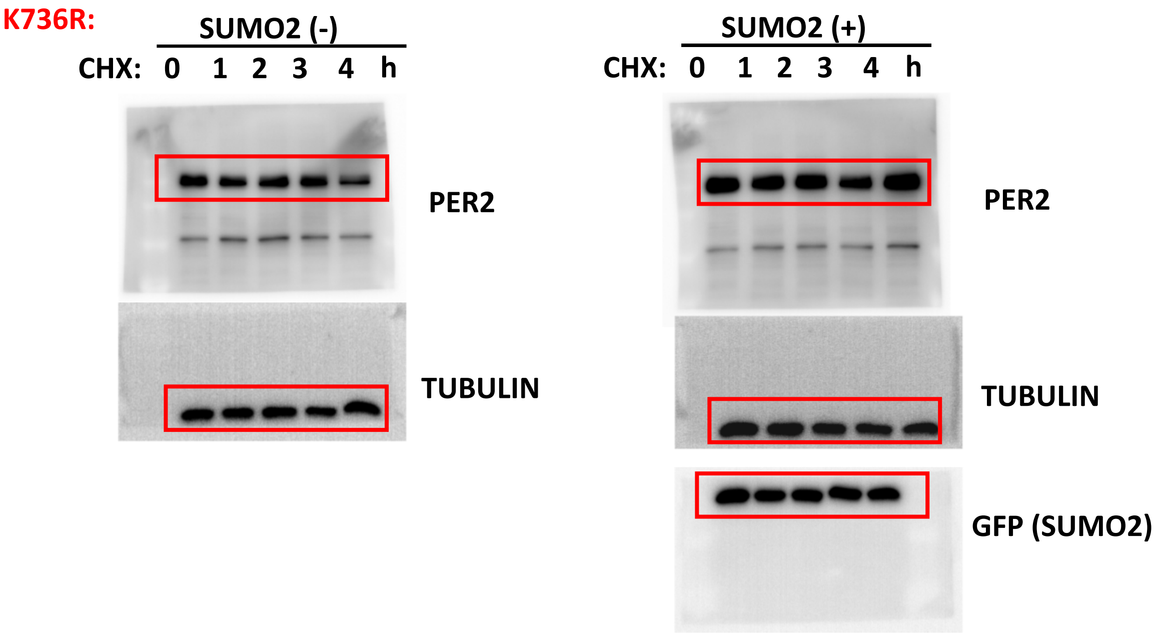

K736R:
SUMO2 (-)
CHX: 0 1 2 3 4 h
PER2
TUBULIN
SUMO2 (+)
CHX: 0 1 2 3 4 h
PER2
TUBULIN
GFP (SUMO2)

Figure S2a (additional experiments)

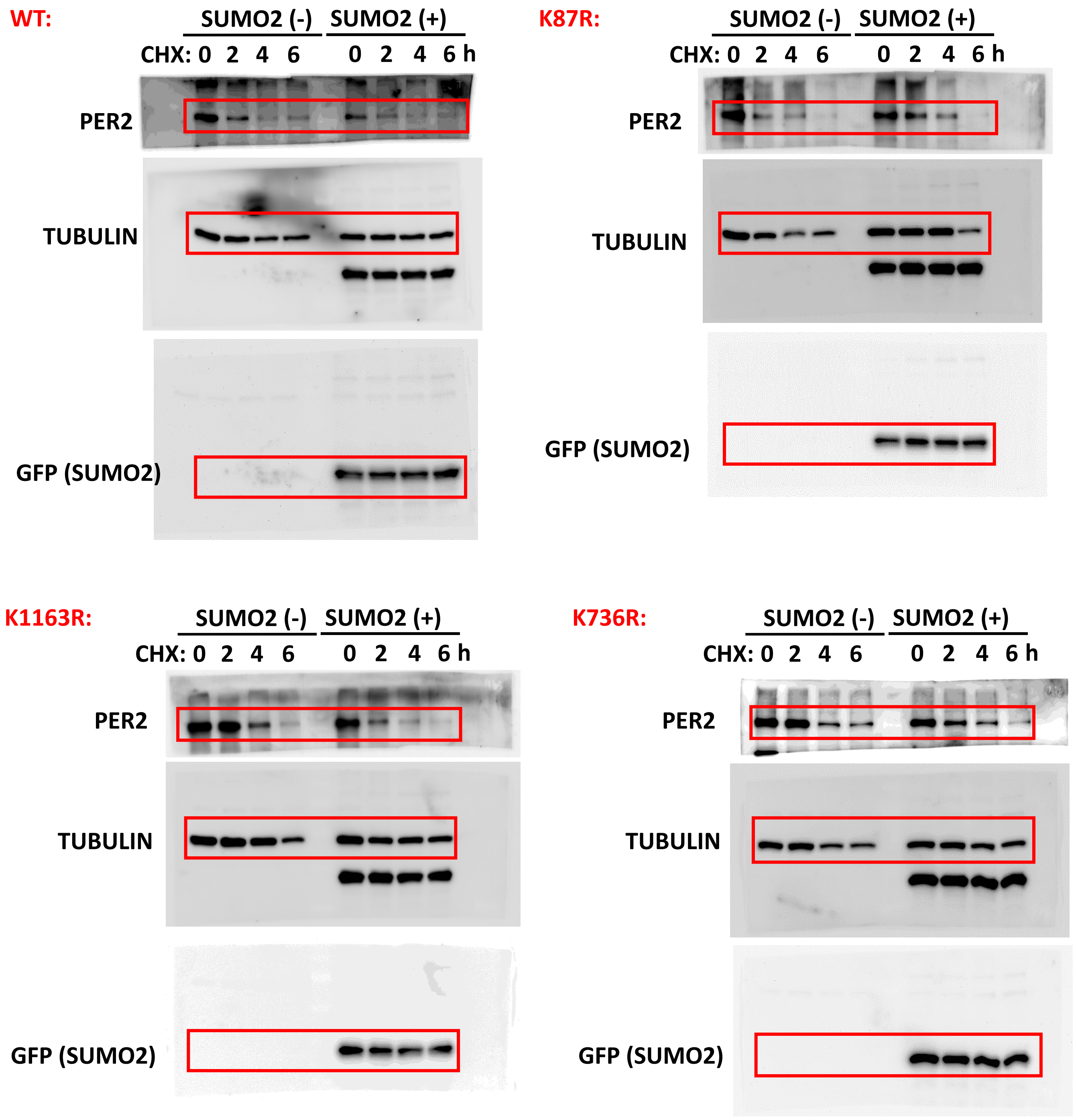

Figure S2b

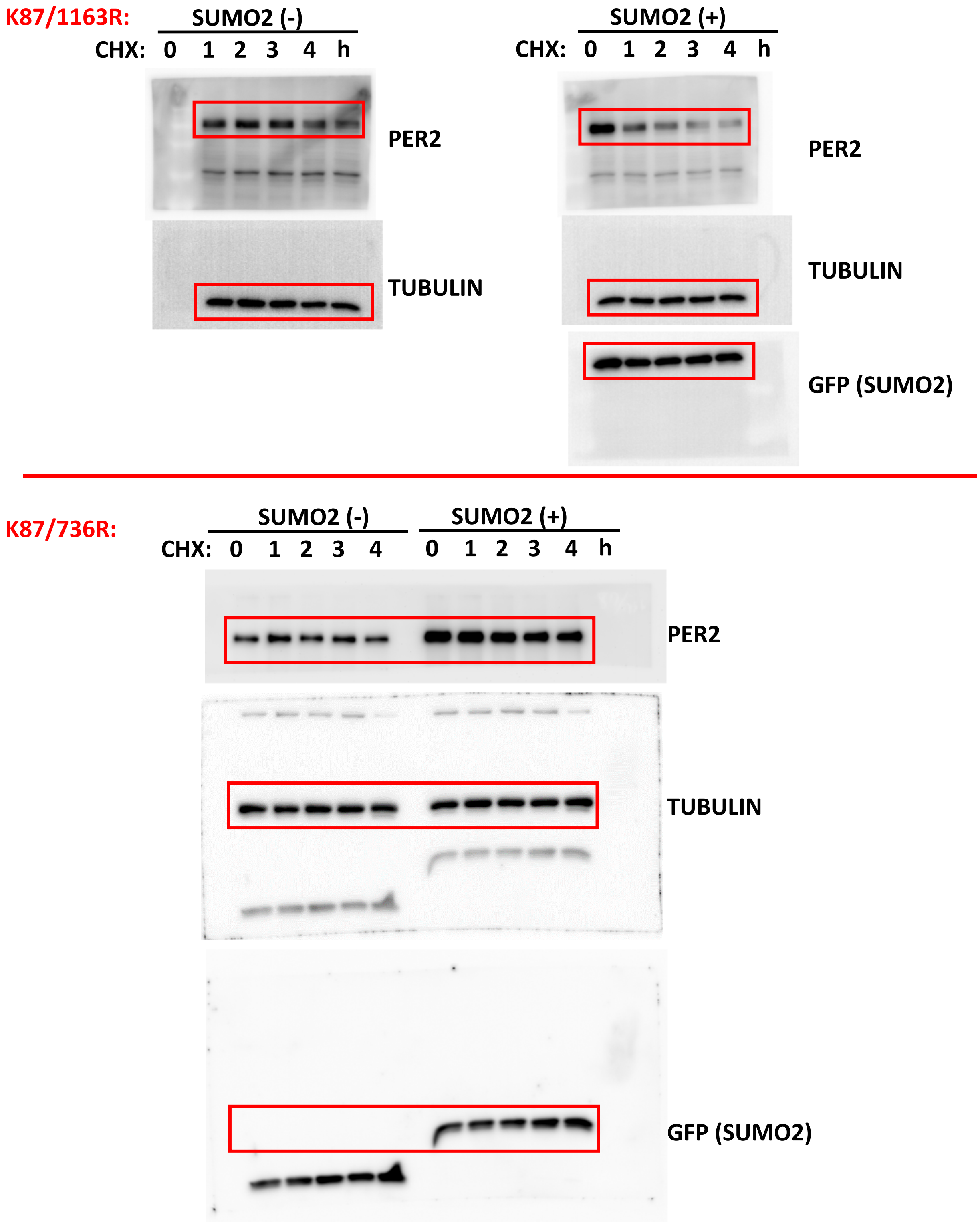
K87/1163R:
SUMO2 (-)
CHX: 0 1 2 3 4 h
SUMO2 (+)
CHX: 0 1 2 3 4 h
PER2
TUBULIN
PER2
TUBULIN
GFP (SUMO2)
K87/736R:
SUMO2 (-)
SUMO2 (+)
CHX: 0 1 2 3 4 0 1 2 3 4 h
PER2
TUBULIN
GFP (SUMO2)

Figure S2b

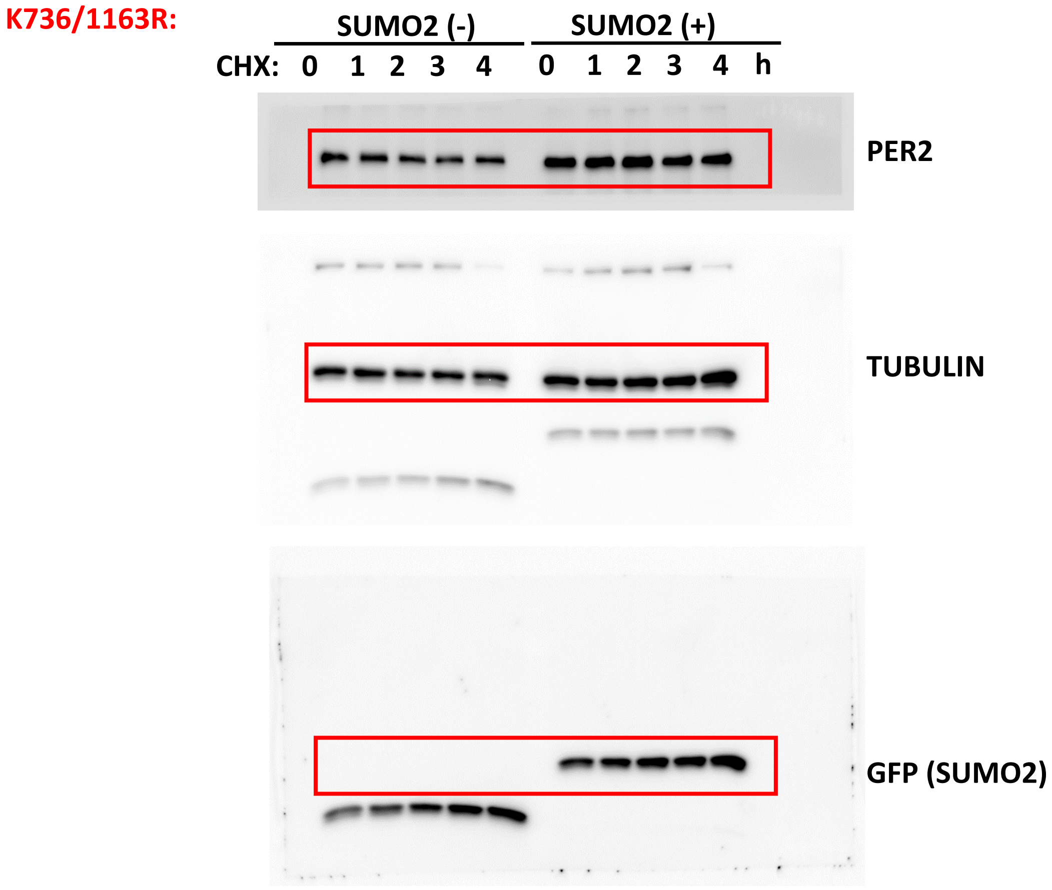
K736/1163R:
SUMO2 (-)
SUMO2 (+)
CHX: 0 1 2 3 4 0 1 2 3 4 h
PER2
TUBULIN
GFP (SUMO2)

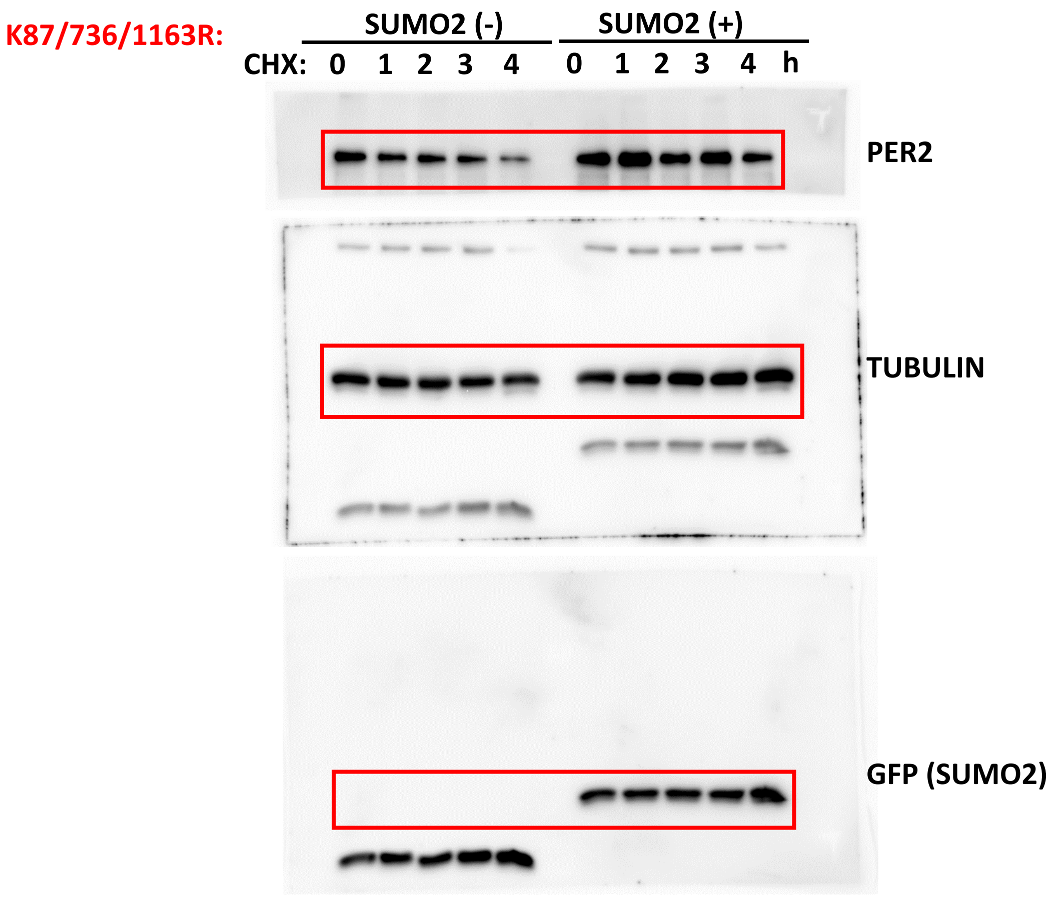
K87/736/1163R:
SUMO2 (-)
SUMO2 (+)
CHX: 0 1 2 3 4 0 1 2 3 4 h
PER2
TUBULIN
GFP (SUMO2)

Figure S2b (additional experiments)

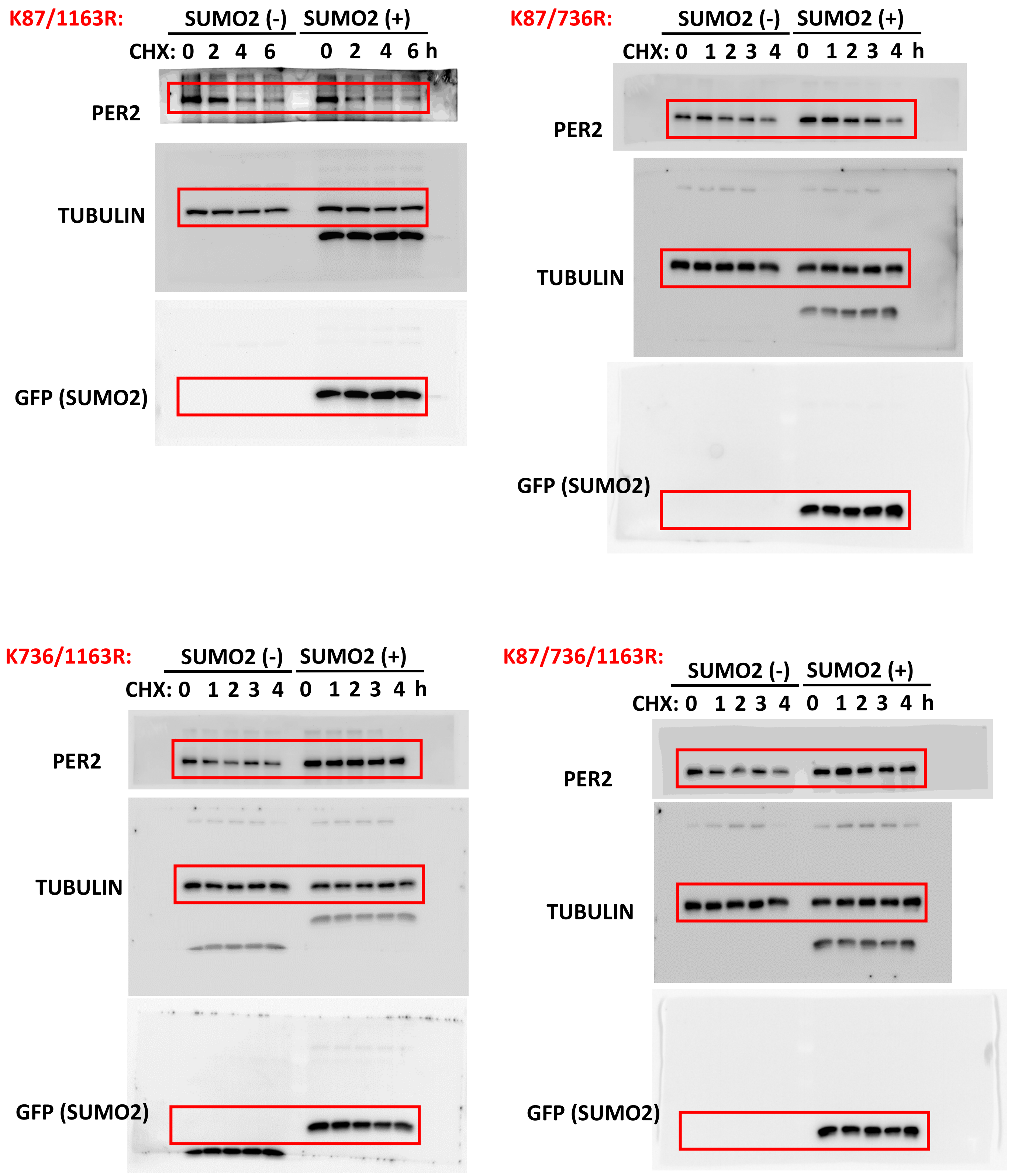

Figure S2c

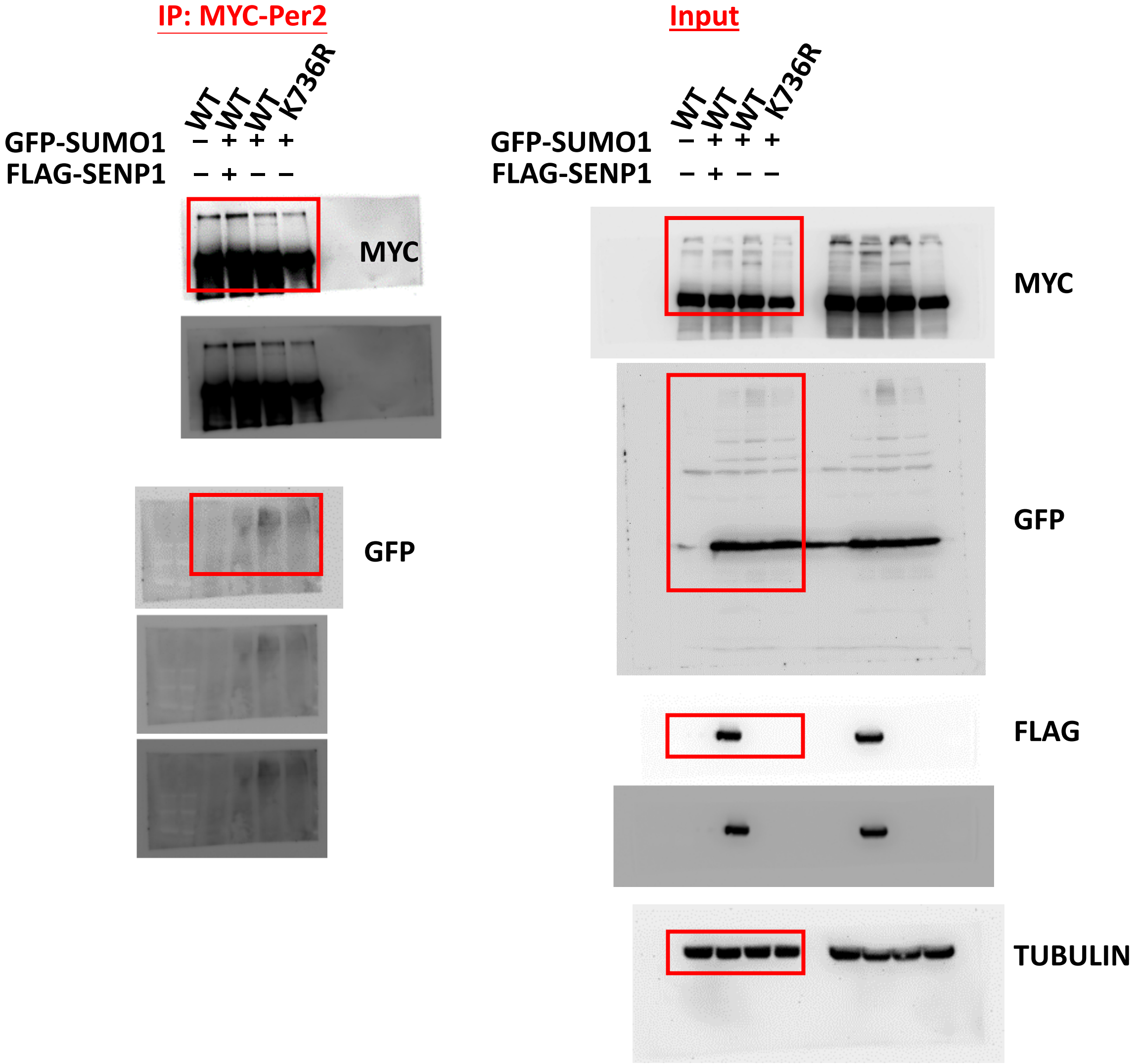
IP: MYC-Per2
Input
WT
WT
WT
K736R
GFP-SUMO1
FLAG-SENP1
MYC
GFP
FLAG
TUBULIN

Figure S4a

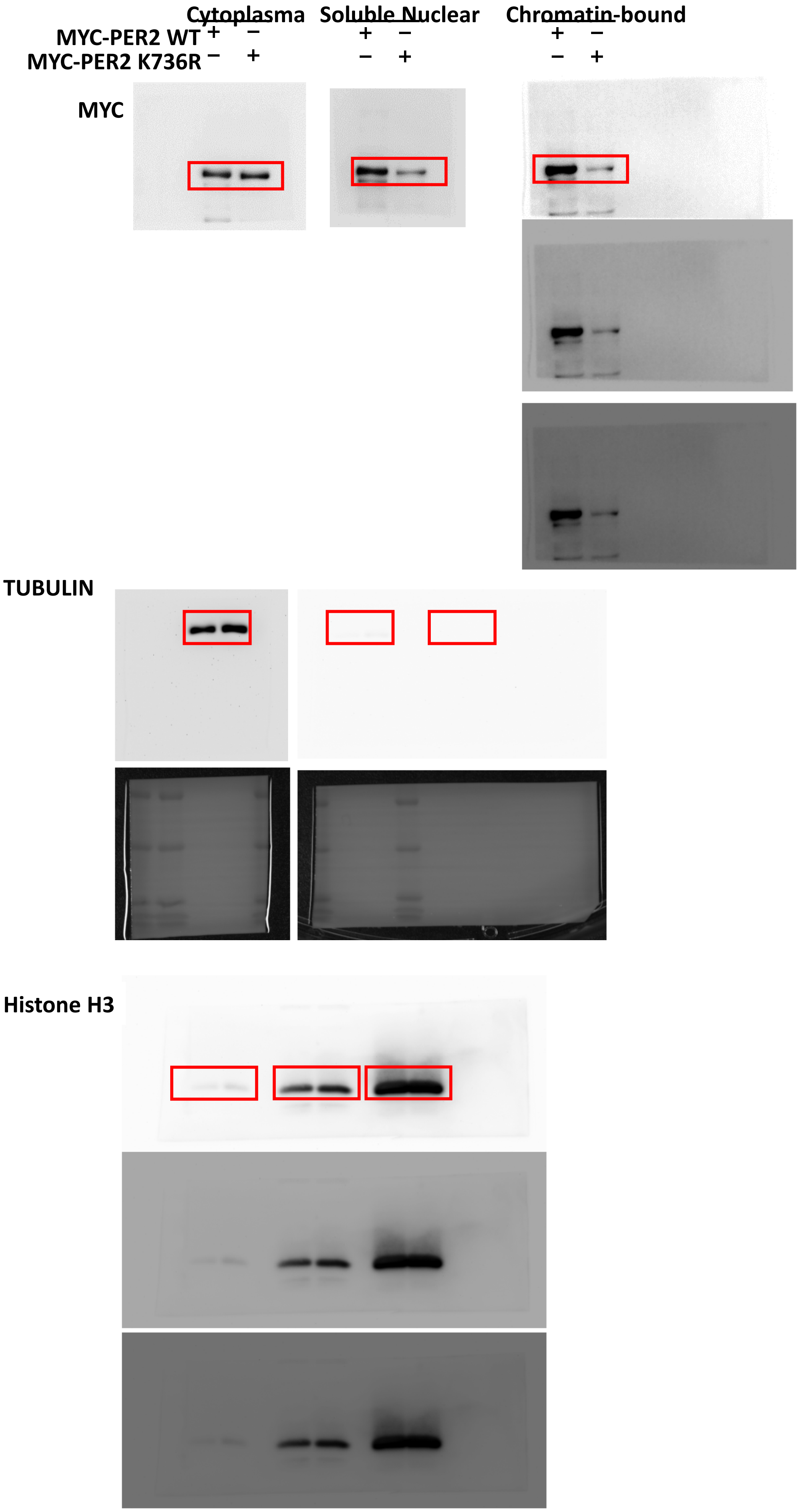
Cytoplasma
Soluble Nuclear
Chromatin-bound
MYC-PER2 WT + − + − + −
MYC-PER2 K736R − + − + − +
MYC
TUBULIN
Histone H3

Figure S4b

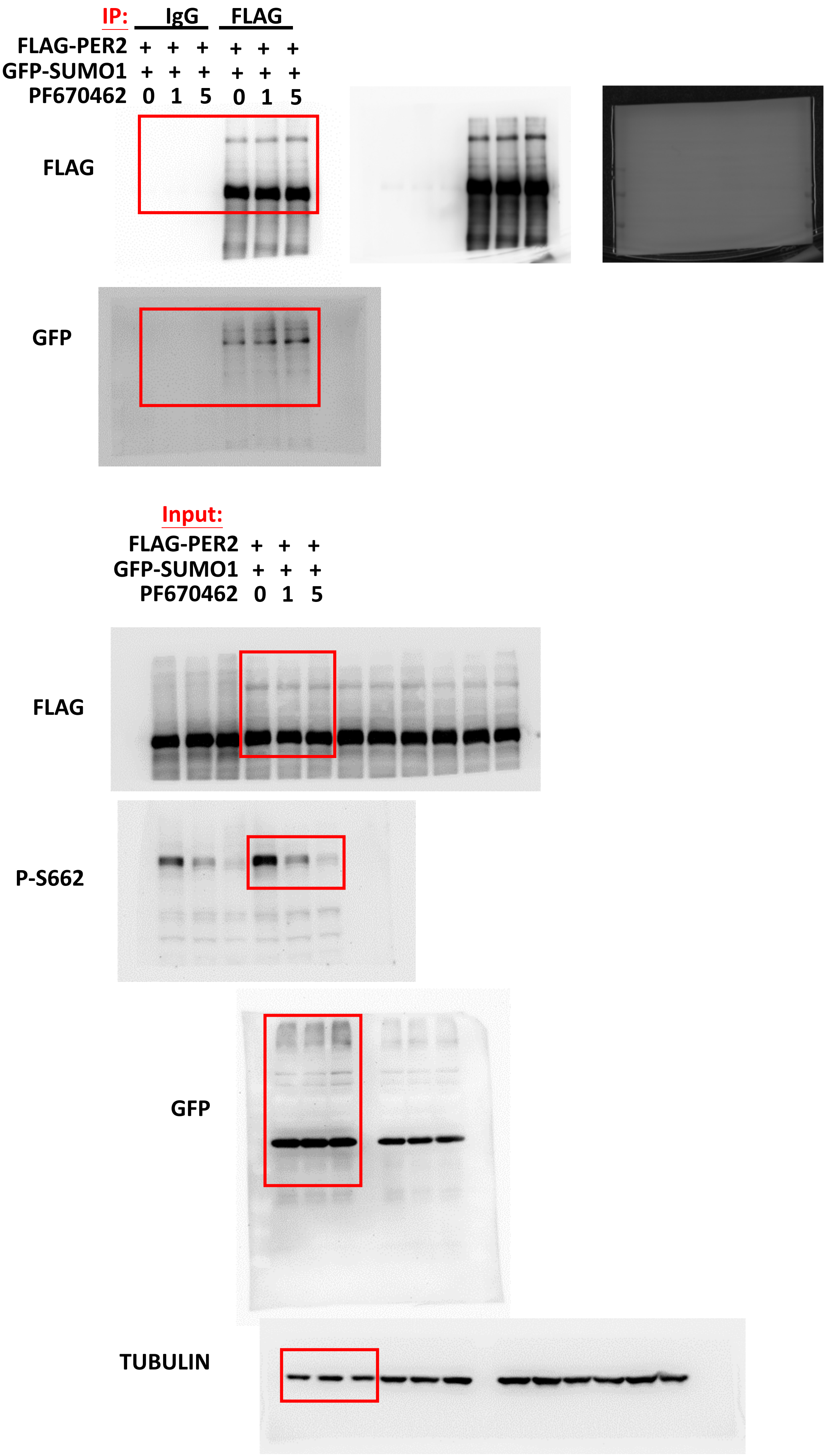

IP: IgG FLAG
FLAG-PER2 + + + + + +
GFP-SUMO1 + + + + + +
PF670462 0 1 5 0 1 5
FLAG
GFP
Input:
FLAG-PER2 + + +
GFP-SUMO1 + + +
PF670462 0 1 5
FLAG
P-S662
GFP
TUBULIN

Figure S4b (additional experiments)

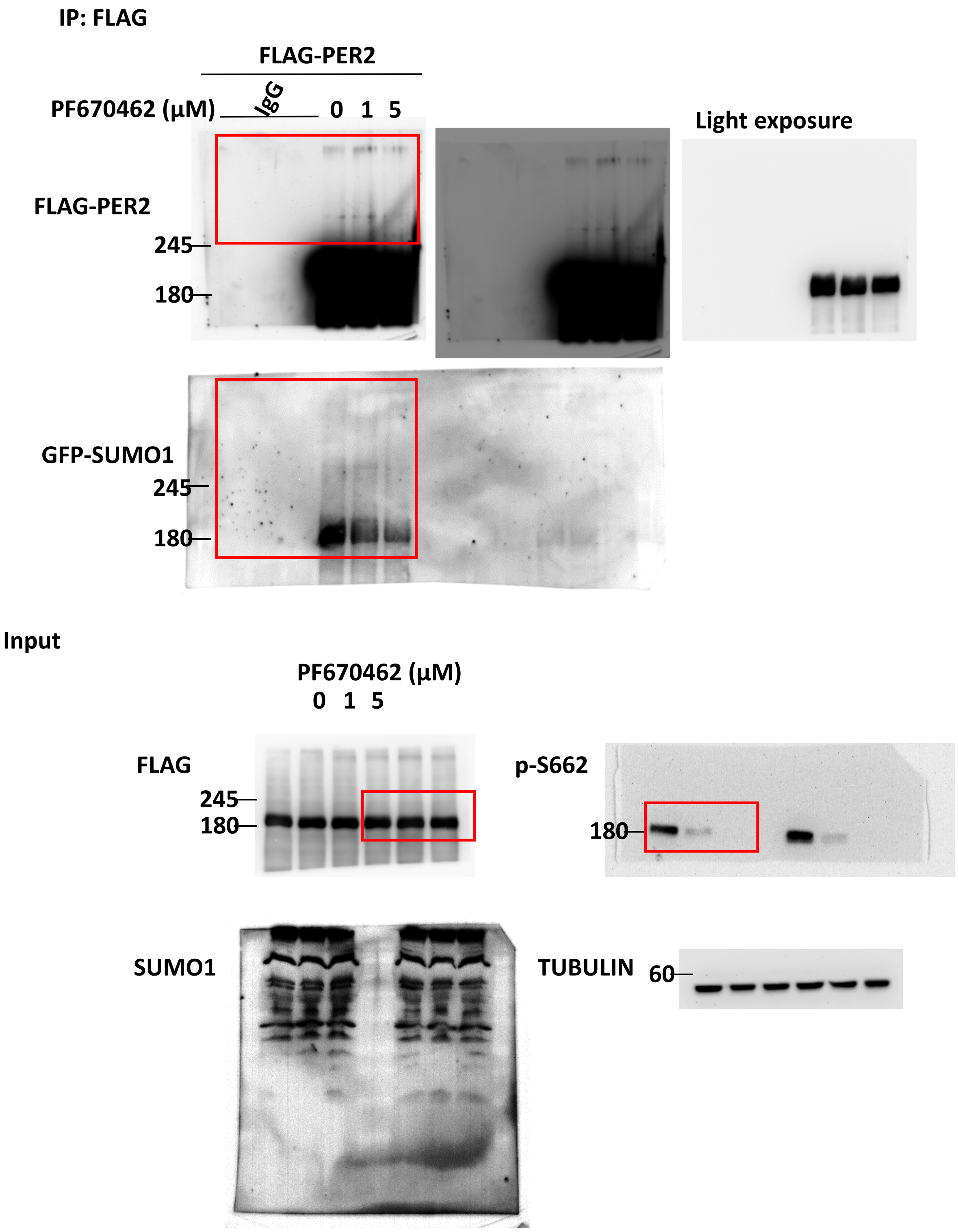

Figure S4c

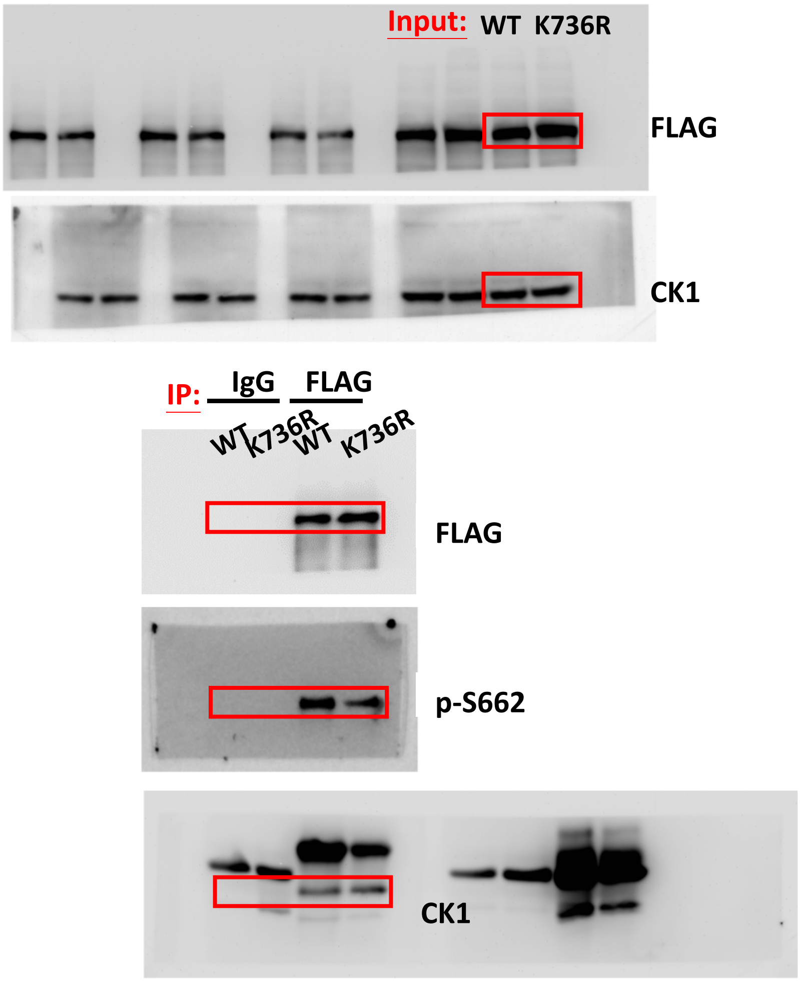
Input:
WT
K736R
FLAG
CK1
IP:
IgG
FLAG
WT
K736R
WT
K736R
FLAG
p-S662
CK1

Figure S4c (additional experiments)

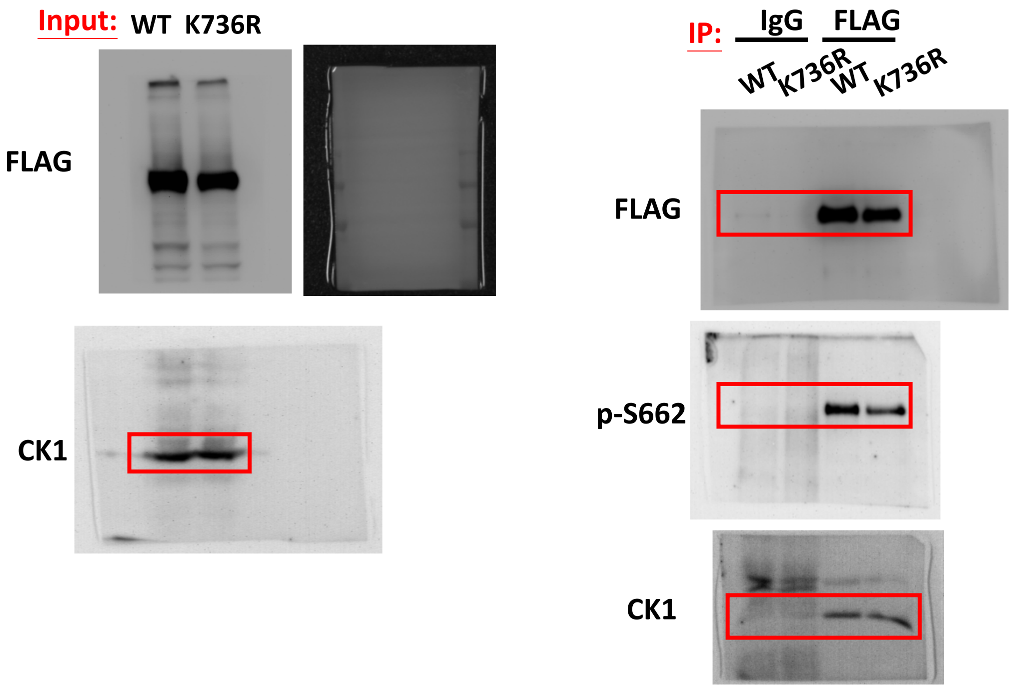

Figure S4d

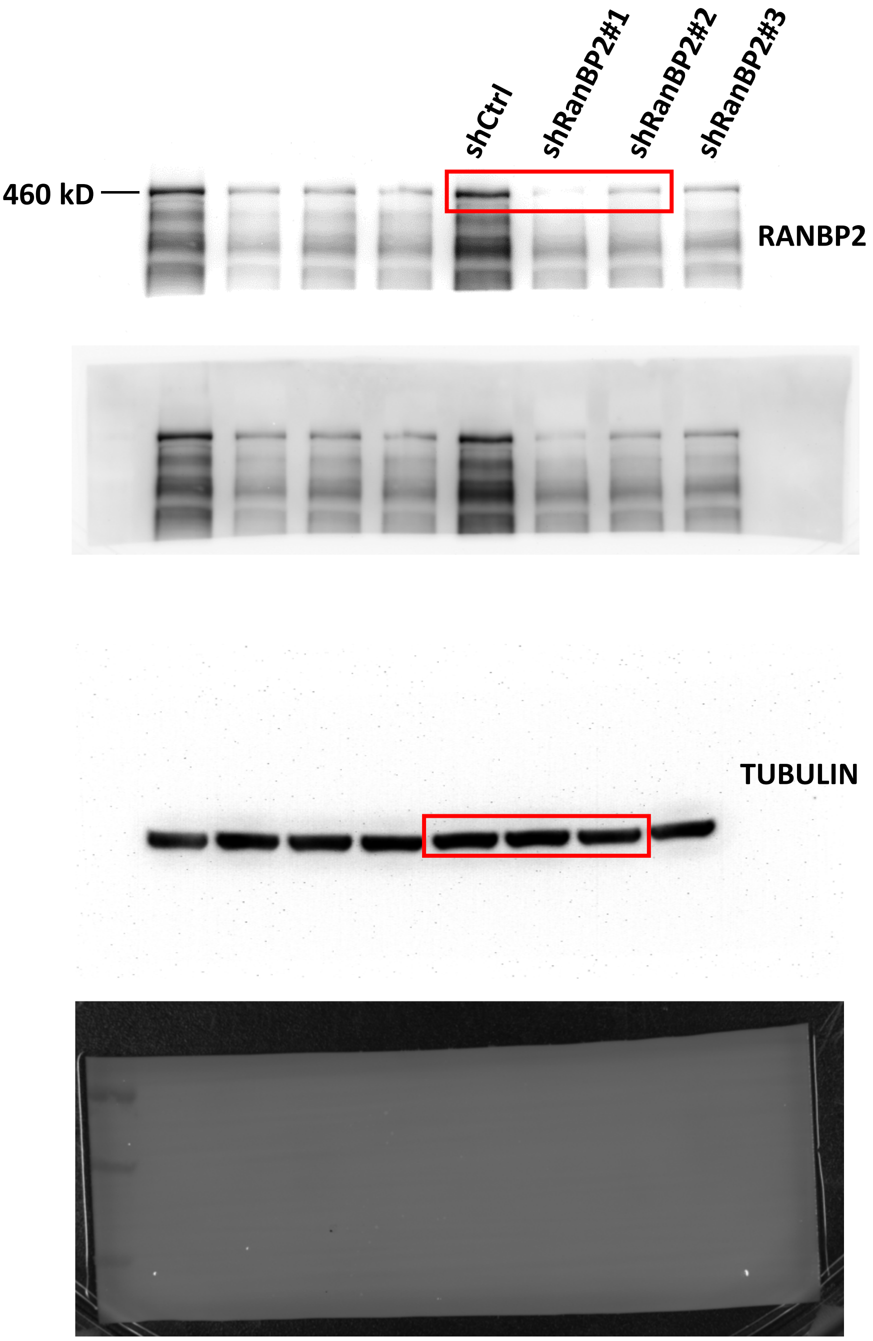

shCtrl
shRanBP2#1
shRanBP2#2
shRanBP2#3
460 kD
RANBP2
TUBULIN

Figure S4e

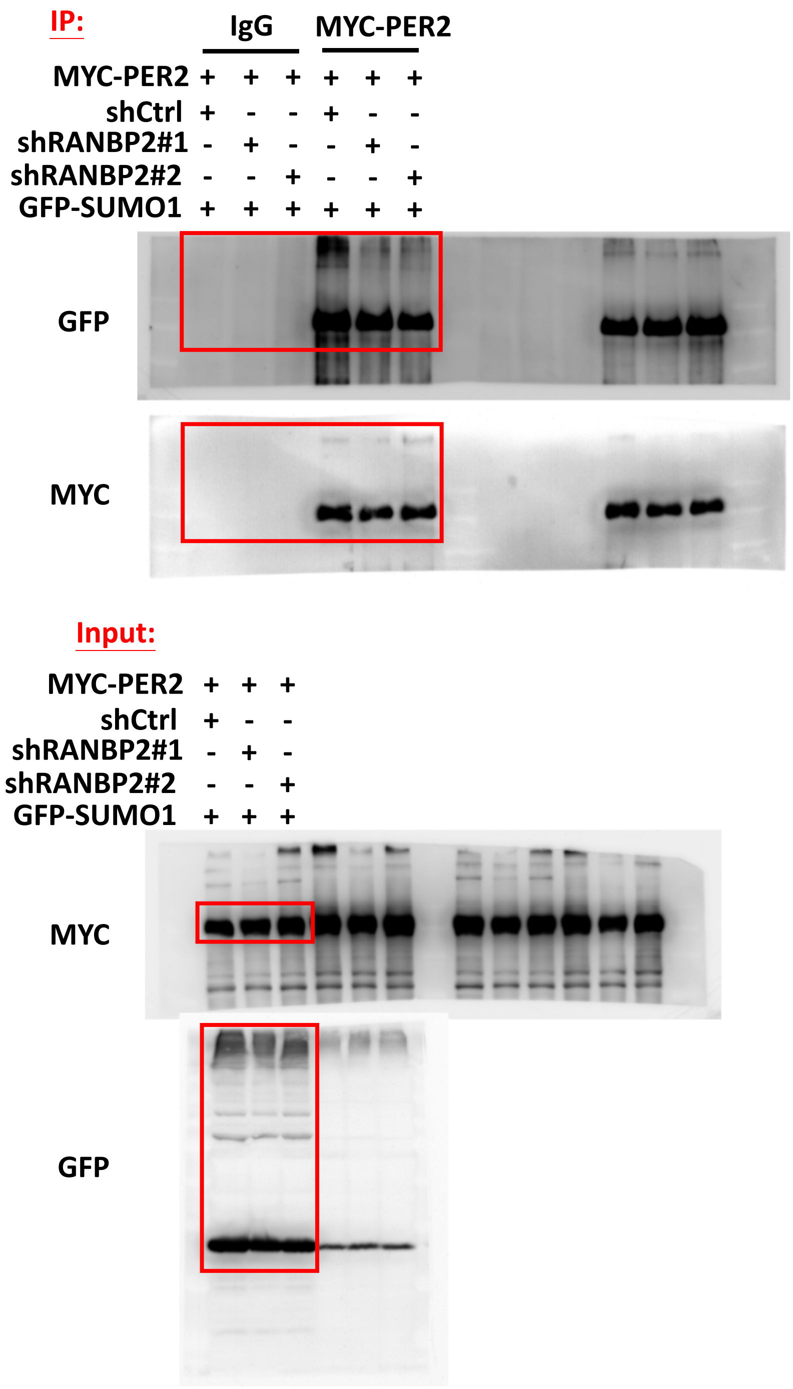

IP:
IgG
MYC-PER2
MYC-PER2 + + + + + +
shCtrl + - - + - -
shRANBP2#1 - + - - + -
shRANBP2#2 - - + - - +
GFP-SUMO1 + + + + + +
GFP
MYC
Input:
MYC-PER2 + + +
shCtrl + - -
shRANBP2#1 - + -
shRANBP2#2 - - +
GFP-SUMO1 + + +
MYC
GFP

Figure S4e (additional experiments)

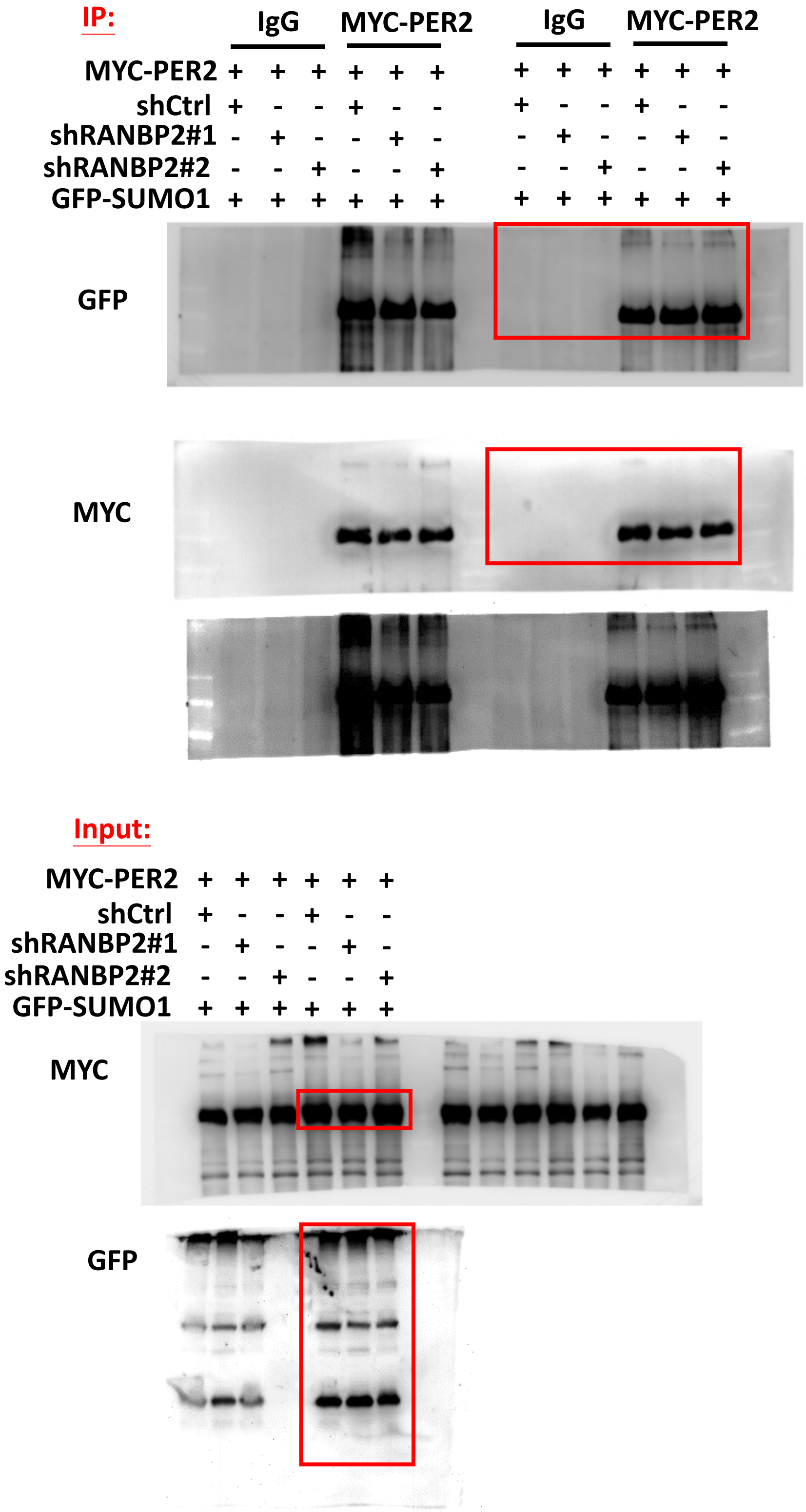

Supplement: Supplementary file 1 — Supplementary Information. [file 41598_2021_93933_MOESM1_ESM.pdf]
